# Tuning Circular Dichroism and Circularly Polarised Luminescence in Single Crystals of a Perylene Diimide Macrocycle

**DOI:** 10.1002/anie.202520567

**Published:** 2026-02-03

**Authors:** Denis Hartmann, Samuel E. Penty, Artemijs Krimovs, Robert Pal, Tiberiu‐M. Gianga, Giuliano Siligardi, Timothy A. Barendt

**Affiliations:** ^1^ University of Birmingham School of Chemistry Edgbaston Campus Birmingham B15 2TT UK; ^2^ University of Durham Department of Chemistry South Road Durham DH1 3LE UK; ^3^ Diamond Light Source Harwell Science & Innovation Campus Didcot OX11 0DE UK

**Keywords:** Chiroptical materials, Macrocycle, Perylene diimides, Single crystals, Supramolecular chemistry

## Abstract

Chiral materials that manipulate circularly polarised light have burgeoning applications across optoelectronics, sensing and information encoding, yet the functionality of organic molecular materials is often limited by their relatively low dissymmetry factors (*g*_abs/lum_ < 10^−^^2^), including towards the near infrared (*λ* > 700 nm). An effective strategy to amplifying *g*_abs/lum_ is to optimise the chiral arrangement of chromophores, with single crystals providing intrinsic molecular ordering. Herein, we quantify the circular dichroism and circularly polarised luminescence of single crystals of a chiral L‐valinol bis‐perylene diimide macrocycle by Mueller–Matrix polarimetry and circularly polarised luminescence microscopy, as required for the analysis of such anisotropic materials. Through this, we see that organic crystals are valuable for understanding how supramolecular structure can be used to modify the sign, strength and energy of the chiroptical signal. Indeed, by tuning the macrocycle's π–π stacking interactions, our materials deliver strong chiroptical properties (*g*_abs/lum _> 10^−^^2^), including circularly polarised luminescence into the near infrared (*λ* = 780 nm).

## Graphical abstract

The circular dichroism (CD) and circularly polarised (CPL) of a bis‐perylene diimide macrocycle are measured in single crystals. These chiroptical properties are tuned by the macrocycle's π–π stacking interactions in the solid‐state.

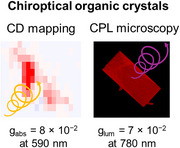

## Introduction

Chiral materials possessing circular dichroism (CD) and circularly polarised luminescence (CPL) are of increasing importance for advanced optoelectronics, sensing and computing.^[^ ^1^, ^2^, ^3^ ^]^ In this context, organic materials^[^ ^4^ ^]^ provide a number of potential benefits including synthetic tuneability,^[^ ^5^ ^]^ solution processability^[^ ^6^ ^]^ and biocompatibility.^[^ ^7^ ^]^ A key performance metric for chiroptical materials is their dissymmetry factor, *g*,^[^ ^8^ ^]^ which quantifies the degree of circularly polarised light that is absorbed (*g*_abs_) or emitted (*g*_lum_). However, the low dissymmetry factors of molecular materials composed of chiral organic chromophores present a significant barrier to practical applications (*g*_abs/lum_ < 10^−^^2^).^[^ ^9^ ^]^ Furthermore, chiroptical materials that interact with low energy circularly polarised light, towards the near infrared (*λ* > 700 nm), are desirable for security,^[^ ^10^, ^11^ ^]^ communication^[^ ^12^ ^]^ and bioimaging^[^ ^13^, ^14^, ^15^ ^]^ technologies, the latter due to deeper tissue penetration.^[^ ^16^, ^17^ ^]^ Notably, the spectral window *λ* = 700–850 nm is fully compatible with conventional photodetectors, yet remains significantly underexplored, in part because most current organic chiroptical materials absorb/emit at higher energy, *λ* < 600 nm.^[^ ^18^, ^19^ ^]^

Alongside the chromophore's covalent structure,^[^ ^20^ ^]^ supramolecular chemistry provides an effective strategy for tuning bandgaps^[^ ^21^, ^22^ ^]^ and boosting dissymmetry factors by directing the chiral ordering of (macro)molecular building blocks.^[^ ^23^, ^24^ ^]^ This includes in solid‐state materials, specifically thin film^[^ ^25^ ^]^ and, for small molecules,^[^ ^26^ ^]^ single crystal materials. The latter are of particular interest since X‐ray diffraction (XRD) can be used to characterise the molecular structure and supramolecular assembly of organic chromophores. This is of fundamental importance because connections between (chiral) structure/arrangement and (chir)optical properties are critical to enabling the design of functional chiral materials. Crystallinity in general has also been connected to improvements in stability,^[^ ^27^ ^]^ charge transport^[^ ^28^, ^29^, ^30^, ^31^, ^32^, ^33^ ^]^ and luminescence.^[^ ^34^, ^35^, ^36^, ^37^, ^38^, ^39^, ^40^ ^]^ However, there are only a handful of organic crystalline materials^[^ ^41^, ^42^, ^43^, ^44^, ^45^, ^46^, ^47^, ^48^, ^49^, ^50^, ^51^, ^52^ ^]^ that have been investigated for their chiroptical properties, with the lowest energy CD and CPL currently at *λ* = 550^[^ ^51^ ^]^ and 700 nm^[^ ^46^ ^]^ respectively (Figure S1‐1).

Such quantitative analyses are rare due to the challenges associated with handling single crystals (e.g., size, morphology) and, perhaps most importantly, their intrinsic anisotropy. When measuring CD (and circular birefringence [CB]), the anisotropy of crystalline materials can give rise to strong linear dichroism (LD) (and linear birefringence [LB]),^[^ ^53^ ^]^ in comparison to isotropic solutions.^[^ ^54^, ^55^, ^56^ ^]^ This is problematic since a significant LD, and indeed LB and CB, can substantially affect the spectral shape, magnitude, and sign of the CD signal measured with benchtop instruments. Mueller–Matrix polarimetry (MMP) may be employed to measure CD in anisotropic materials since this technique enables the individual CD/CB/LD/LB contributions to be decomposed to assess chiroptical homogeneity.^[^ ^55^, ^57^ ^]^ Therefore, MMP is an essential tool for measuring CD spectra in the visible region and then selecting an appropriate wavelength for mapping CD across a material.^[^ ^58^ ^]^ However, to the best of our knowledge, such studies are yet to be undertaken with single crystal materials. For CPL measurements on single crystals, difficulties arise from reflections that depolarise the emitted circularly polarised light, reducing the accuracy of the detected CPL. This is addressed using CPL‐laser scanning confocal microscopy (CPL‐LSCM),^[^ ^45^, ^59^ ^]^ and imaging single crystals of suitable size (i.e., an order of magnitude larger than the microscope resolution) and orientation (perpendicular to the optical axis). However, CPL‐microscopy measurements of solid‐state materials are rare.^[^ ^45^, ^60^ ^]^

**Figure 1:**
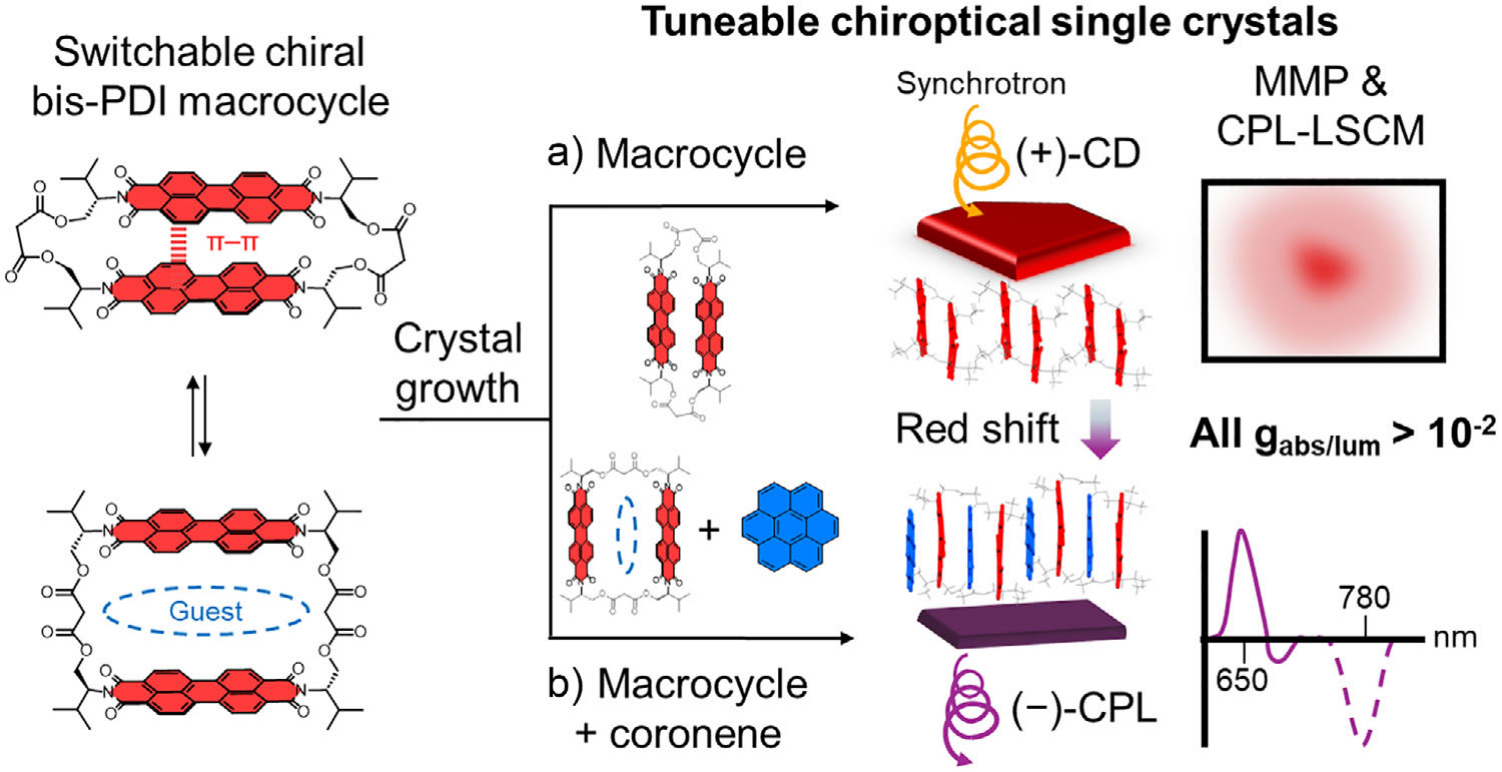
A switchable chiral bis‐perylene diimide macrocycle affords tuneable chiroptical single crystal materials. In a) macrocycle‐only single crystals, the circular dichroism (CD) and circularly polarised luminescence (CPL) are quantified by Mueller–Matrix polarimetry (MMP) and CPL‐laser scanning confocal microscopy (CPL‐LSCM); and in b) macrocycle–coronene co‐crystals, guest binding affords redshifted chiroptical properties, including CPL in the near‐infrared.

Perylene diimides (PDIs) are a class of organic dyes with great promise as chiroptical materials^[^ ^61^ ^]^ due to their pronounced photophysical properties and potential for chiral self‐assembly.^[^ ^62^ ^]^ There has been a growing interest in using PDI‐based macrocycles^[^ ^63^ ^]^ as (chiral)^[^ ^64^, ^65^ ^]^ building blocks for (chiral)^[^ ^45^ ^]^ materials because of their increased dimensionality, shape persistence and supramolecular chemistry.^[^ ^66^, ^67^ ^]^ Indeed, we have recently developed a bis‐PDI macrocycle that exhibits switchable (chir)optical properties in solution, due to the population of distinct (chiral) conformations that afford intramolecular π–π stacking or aromatic guest binding via intermolecular π–π interactions (Figure 1, left).^[^ ^68^ ^]^

Herein, we examine our chiral bis‐PDI macrocycle as a building block for solid‐state chiroptical materials, quantifying its chiroptical properties in single crystals using MMP and CPL spectroscopy and microscopy, to avoid artefacts from linear polarisation (Figure 1a). These studies are important in highlighting the design features of chiral organic molecules and their supramolecular assemblies that can be used to optimise and tune their chiroptical properties. Notably, bis‐PDI macrocycle single crystals outperform thin films in terms of dissymmetry factor (10^−^^2 ^versus 10^−^^3^) due to improved homogeneity of the π–π dimer and its long‐range ordering in a chiral environment. Furthermore, host–guest macrocycle–coronene co‐crystals reveal how intermolecular charge transfer interactions modify the supramolecular structure, providing a valuable route to increasing *λ* whilst maintaining high *g* (Figure 1b). These materials deliver far‐visible CD (*λ* = 640 nm) and near‐infrared CPL (780 nm), both with *g* > 10^−^^2^.

## Results and Discussion

### Macrocycle Materials

**Figure 2:**
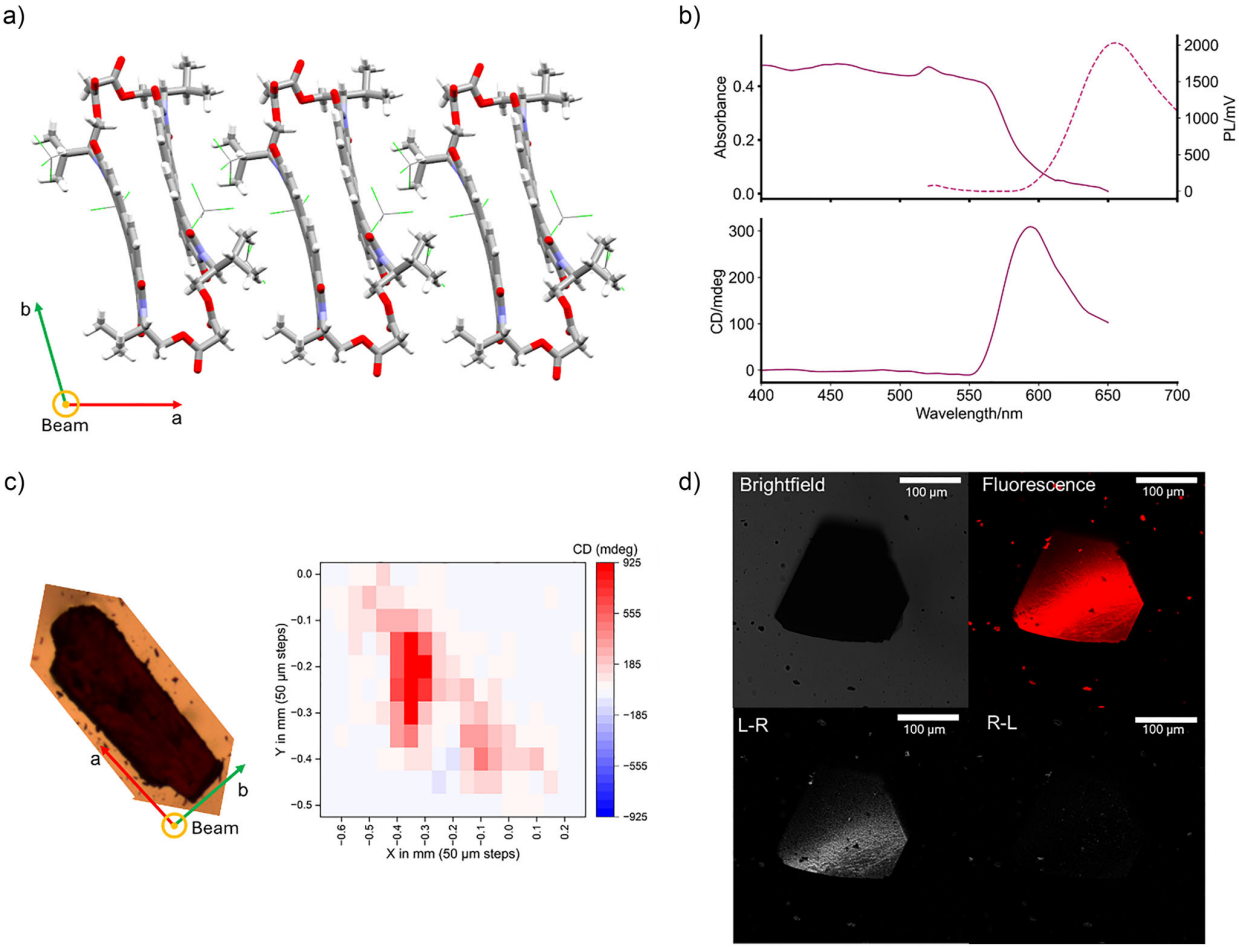
(Chir)optical properties of L‐valinol macrocycle single crystals. a) XRD single crystal structure as viewed along the short (c) axis of the crystal, which is the optical axis for MMP and CPL‐LSCM measurements. b) Absorbance (solid line), photoluminescence (PL, dashed line, *λ*_ex_ = 458 nm) and MMP CD spectra of a macrocycle single crystal (smoothed). c) Microscope image and its MMP CD map (*λ* = 590 nm) of a macrocycle single crystal (16 elements MM and full Analytic Inversion calculation reported in Figures S3–12, S13, respectively). d) (CPL‐)LSCM images of a macrocycle single crystal (*λ*_ex_ = 458 nm, *λ*_em_ > 570 nm), with the left‐ (L − R) and right‐handed (R − L) CPL images showing the enantioselective differential chiral contrast (bottom).

The L‐valinol‐based bis‐PDI macrocycle (Figure 1) readily crystallises into flat, trapezoidal plates up to 0.5 × 0.5 mm in size (Figure 2c, left).^[^ ^68^ ^]^ Whilst growth is facilitated along the crystal's *a* and *b* axes by hydrogen bonding (long axes), growth in the *c* axis (short axis) is hindered due to an absence of intermolecular interactions, with the macrocycles separated by a layer of CHCl_3_ solvent molecules (Figure 2a). These axes were identified by comparison of crystal morphology to the single crystal X‐ray diffraction data (Figure S1–3). The molecule crystallised in triclinic space group *P*1, confirming a chiral crystal lattice. Barring translation, this chiral space group is characterised by an absence of symmetry elements and is known to give rise to chiroptical properties.^[^ ^46^, ^69^, ^70^, ^71^ ^]^ The L‐valinol bis‐PDI macrocycle is chiral (molecular symmetry = *C*_1_)^[^ ^72^ ^]^ and adopts a “closed” conformation due to co‐facial intramolecular π–π stacking (*d*_π–π_ = 3.3 Å). This π–π dimer structure is consistent with excitonic coupling between the two PDI units (predominantly H‐type),^[^ ^62^, ^73^ ^]^ which is important since chromophore coupling in chiral materials may amplify dissymmetry factors.^[^ ^9^ ^]^

We measured the absorbance and CD of three individual macrocycle single crystals (**1**–**3**) by MMP (Figure 2b,c), using the highly collimated B23 beam at Diamond Light Source.^[^ ^58^ ^]^ MMP at B23 provides a 50 µM spatial resolution and a high photon flux and, aside from single crystals, has been employed to measure CD in a range of chiral solid‐state materials.^[^ ^74^, ^75^, ^76^, ^77^, ^78^, ^79^ ^]^ For our MMP measurements of single crystals, we placed the macrocycle crystals on a quartz slide using an inert, fluorinated oil, Fomblin Y, for ease of handling and stability. The Fomblin background was measured by MMP (Figures S3–2 and S3–3) and always subtracted from that of the crystals. Due to their consistent habit, the crystal plates were consistently oriented flat on the slide and hence MMP spectra were always collected in the direction of the short axis (Figure 2a). Full details on MMP data collection and processing are available in the Supporting Information (Section 3).

MMP was first performed on single crystals **1** and **2** (Figure S3–4,7), measuring spectra at multiple different locations on **1** (Figure S3–4,5). The absorbance spectra at these points were consistent and showed an absorption edge at *λ* = ∼ 600 nm (bandgap = ∼ 2 eV, Figure 2b and S3–8), which agrees with that obtained from a bulk measurement (Kubelka–Munk transformation of the reflectance spectrum, Figure S2–1). MMP data was only analysed within the absorption window of the crystals. The macrocycle crystals possess a (+) CD signal at the absorption edge^[^ ^46^ ^]^ (Figure 2b) with a high dissymmetry factor, *g*_abs_ up to 8 × 10^−^^2^ at *λ* = 590 nm. We note that whilst differential light scattering through a periodic lattice may distort the CD,^[^ ^80^ ^]^ this effect is negligible in the B23 MMP instrument since the sample is positioned significantly further from the detector (40 cm) in comparison to that in a conventional benchtop CD spectropolarimeter (∼ 5 cm).^[^ ^81^ ^]^

The absence of a bisignate Cotton effect in the CD spectrum of crystals **1** and **2** is consistent with the co‐linear arrangement of the two PDI chromophores in the macrocycle, as seen in the crystal structure (Figure S1–2). From MMP, the single crystal *g*_abs_ is over an order of magnitude larger than that of thin films (Table 1) and in solution.^[^ ^68^ ^]^ This may be explained by rigidification of the chiral PDI dimer in the solid‐state (versus solution), with single crystals exhibiting superior homogeneity and long‐range ordering of this structure in the chiral single crystal lattice relative to amorphous films.

**Table 1:** The chiroptical properties of the L‐valinol macrocycle and L‐valinol macrocycle–coronene solid‐state materials.

		|*g*_abs_| × 10^−3^ (*λ*)^a)^		
Composition	Material	PDI	Coronene	|*g*_lum_| × 10^−3^ (λ)^b)^
Macrocycle	Crystal	80 (590 nm)	–	80 (> 570 nm)^c)^
	Thin film	1 (502 nm)	–	–
Macrocycle–coronene	Crystal	90 (644 nm)^d)^	20 (406 nm)^d)^	70 (780 nm)^e)^

^a)^Error = ±1 × 10^−4^.

^b)^Error = ±5 × 10^−5^.

^c)^ *g*_EDCC_ value, as determined by CPL‐LSCM.

^d)^Obtained as an average of three single crystals.

^e)^Obtained from a sample of microcrystals (Figure S4–2).

Whilst the L‐valinol PDI dimer is chiral, we hypothesise that off‐resonance coupling^[^ ^82^ ^]^ (also known as “induced CD”) between macrocycles and the chiral environment within the crystal also contribute to the chiroptical properties of the crystals. This is because time dependent‐density functional theory (TD‐DFT) calculations (Supporting Information, section 8) indicate the importance of the intermolecular hierarchical structure of chiral macrocycles in the crystal since the predicted CD of one unit cell containing a single macrocycle (Supporting Table 8–1) does not match the sign of the CD measured by MMP (Figure 2b).^[^ ^83^ ^]^ An understanding of the CD of crystalline materials from first principles is challenging^[^ ^84^ ^]^ because the complex environment of a crystal will generate multiple excitations that are close in energy and so a measured CD signal may not be related to a single transition.^[^ ^85^ ^]^ However, to the best of our knowledge, there are currently no periodic TD‐DFT codes available to calculate CD spectra.

As expected for an anisotropic, crystalline material, MMP revealed that both single crystals **1** and **2** exhibit linear dichroism (LD), the differential absorption between horizontally and vertically polarised light. We found (+) LD for crystal **1** and (−) for crystal **2** (Figure S3–5,7). This is because the LD is dependent on crystal orientation^[^ ^54^ ^]^ and, whilst all crystals were irradiated along their short axis, this could be the +/−*c* axis, whilst their orientation in the two‐dimensional *a*,*b* plane of the slide may also change between samples. Therefore, we assessed how the (−) LD contribution in the crystal **2** impacted its (+) CD by measuring a CD spectrum with the MMP instrument operating in CD mode (akin to a benchtop CD instrument). In this manner, the MMP and the CD were measured at the same coordinates on the crystal (Figure S3–9). The new CD spectrum closely resembled the original obtained by MMP, with a similar *g*_abs_ at the absorption edge (on the order of 10^−^^2^). Therefore, in crystal **2**, it appears that the (−) LD observed with the MMP, as well as the LB and CB, which were both significantly smaller than the LD, do not contaminate the CD to a large degree.

We then investigated the chiroptical homogeneity of the crystals by mapping each crystal in full at 50 µM resolution (i.e., 13 × 14 intervals) at the absorption edge (Figures 2c and S3–10,13). As expected for single crystals, their chiroptical properties are homogeneous, with a consistent (+) CD signal across each crystal. The two‐dimensional maps for LD, as well as CB and LB, are also homogeneous. We note that our thickest single crystals could not be measured due to poor signal‐to‐noise. We also mapped a crystal (**3**) that showed twinning, i.e., a larger crystal with a smaller crystal stuck to it. (Figure S3–14,15). In regions away from the twin, the typical (+) CD signal is observed, yet the regions of twinning exhibited a (−) CD, which may be related to their orientational relationship.

The macrocycle single crystals are visibly luminescent and large enough to enable CPL characterisation by enantioselective differential chiral contrast (EDCC) imaging using CPL‐LSCM (Figures 2d and S4–3,7). Here, left‐ and right‐circularly polarized photons are separated and simultaneously collected to generate independent left‐ and right‐handed CPL images^[^ ^59^ ^]^ of single crystals. To eliminate reflections, crystals were positioned with their surfaces (*a*,*b* plane) perpendicular to the microscope's optical axis (i.e., the same orientation as for MMP). The images of the macrocycle crystals reveal (+) CPL at *λ* > 570 nm, consistent with the (+) CD from MMP (Figure 2b). The ratio of left‐ and right‐handed CPL was 2.7:1 at the crystal surface (Supporting Figure 4–5) and rose to 3.0:1 at the crystal edge (Figure S4–6) due to minimised reflections.

We found that the crystals lost their CPL after being left out of their supernatant for several hours. This is because solvent loss from the crystal lattice causes crystal cracking and hence an increase in internal reflections, which depolarise the emitted circularly polarised light (Figure S4–7).^[^ ^86^ ^]^ Whilst this prevented accurate CPL spectroscopy on single crystals, the CPL inactive crystals enabled us to calculate a bias factor for the EDCC images of the intact macrocycle crystals and hence their EDCC dissymmetry factor (*g*_EDCC_), a metric analogous to the *g*_lum_.^[^ ^45^ ^]^ The *g*_EDCC _= 8 × 10^−2^, which is of the same order of magnitude as the CD and competitive with leading CPL organic materials in the solid‐state (Figure S1–1).^[^ ^18^ ^]^ The relatively large Stokes shift of the PL from macrocycle crystals (Δ*λ* = 46 nm from the absorption edge) is indicative of excimer emission (Figure 2b), which is preorganised by the macrocyclic π–π dimer and known to amplify CPL.^[^ ^45^, ^87^, ^88^ ^]^

**Figure 3:**
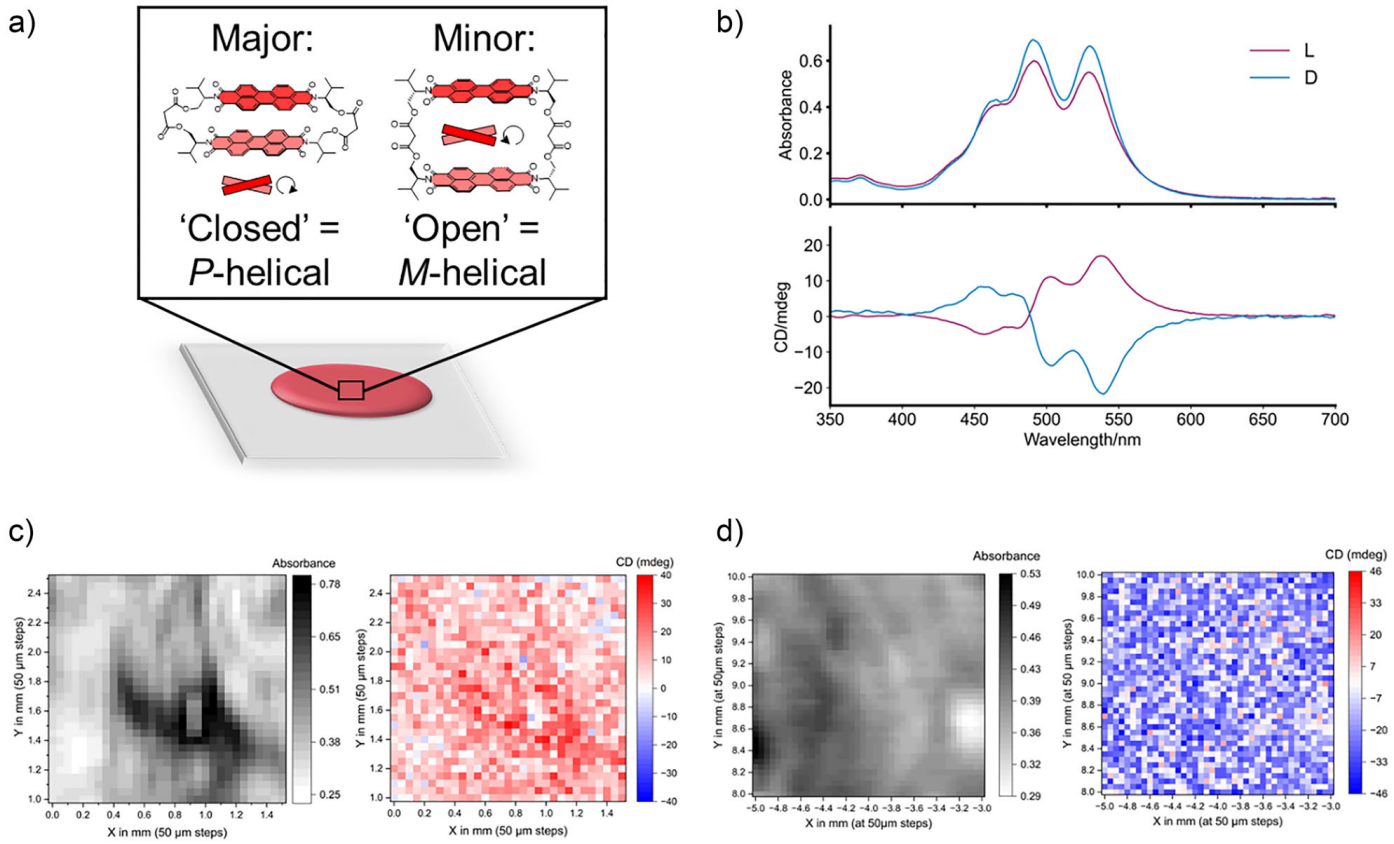
(Chir)optical properties of macrocycle thin films: a) Composition of L‐valinol bis‐PDI macrocycle films, with both *P*‐ and *M*‐helical bis‐PDI macrocycle conformers present. b) Absorption and CD spectra of L‐ and D‐valinol macrocycle films. c) Absorbance and MMP CD map of 1.4 × 1.5 mm area of the L‐valinol macrocycle film (50 µm spatial resolution, *λ* = 546 nm, 16 elements MM and full Analytic Inversion calculation reported in Figure S3–16,17, respectively). d) Absorbance and MMP CD map of 2 × 2 mm area of the D‐valinol macrocycle film (50 µm spatial resolution, *λ* = 546 nm, 16 elements MM and full Analytic Inversion calculation reported in Figure S3–19,20, respectively).

To benchmark the results from single crystals, we also investigated and quantified the L‐valinol macrocycle's CD in thin films (Figure 3 and S7–1), prepared by drop casting from toluene. MMP revealed that the CD is significantly weaker in films^[^ ^89^ ^]^ relative to single crystal materials (Table 1). The CD spectra of nine random locations across the film all showed a (+) bisignate cotton effect for the main PDI absorption band (Figure 3 and S3–16,18), a feature not seen in the crystals. Instead, there is a close agreement between the CD and UV–vis spectra of the macrocycle in films (Figure 3b) with spectra measured in aqueous solution (Figure S2–5).^[^ ^68^ ^]^ This indicates that the macrocycle favours right‐handed chirality in films, specifically a *P*‐helical “closed” intramolecular dimer conformation with co‐facial π–π stacking (H‐type coupling) between the two PDIs (Figure 3a). This assignment of the chiral conformation is also supported by our previous TD‐DFT calculations performed on a L‐valinol bis‐PDI macrocycle structure, obtained after optimisation with implicit solvation.^[^ ^68^ ^]^

Interestingly, however, the ratio between the 0–0 and 0–1 vibronic peak intensities (A_0‐0_/A_0‐1_) in the absorption spectrum of the macrocycle is slightly higher in thin films than in aqueous solution (0.96 versus 0.87), perhaps indicating a small population of the *M*‐helical, “open” conformation (≥ 10%, Figure 3a). Alongside greater disorder in the film, the structural inhomogeneity of the helical π–π dimer itself may explain the poorer chiroptical performance of films compared to crystals; the opposing chirality of the macrocycle conformers will lead to some signal cancelling throughout the material (Figure 3a). Indeed, MMP maps (Figures 3c,d and S3–16,18) revealed uniform CD across the films, meaning that any minor *M*‐helical conformer is randomly distributed amongst the major *P*‐helical dimer.

Whilst we were unfortunately unable to grow single crystals of the D‐valinol bis‐PDI macrocycle, analogous thin films of this opposite enantiomer were prepared to confirm the molecular origin of the chiroptical properties.^[^ ^18^, ^50^ ^]^ Indeed, MMP revealed mirror image CD spectra, with identical *g*_abs_, and CD maps of the opposite sign in comparison to the L‐valinol PDI macrocycle (Figures 3c,d and S3–19,20,23). Racemic thin films were also fabricated by mixing an equimolar ratio of L‐ and D‐valinol macrocycles. These films exhibited negligible CD by MMP (Figure S2–7,21,22), confirming that anisotropic scattering, caused by for example LD and LB, is not contaminating the chiroptical signal.^[^ ^85^ ^]^

### Host–Guest Materials

**Figure 4:**
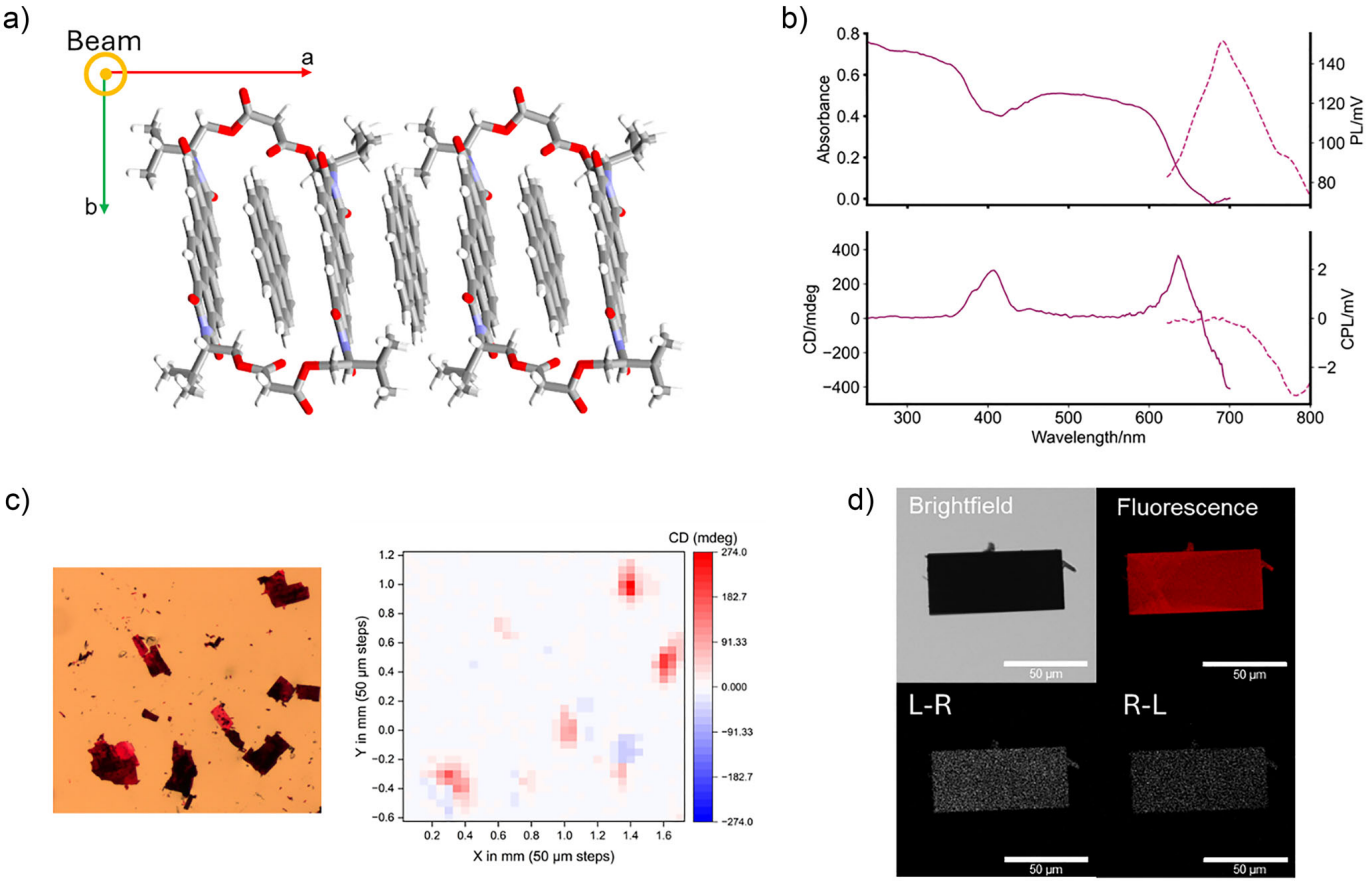
(Chir)optical properties of L‐valinol macrocycle–coronene host–guest co‐crystals. a) XRD single crystal structure as viewed along the short axis (*c*) of the crystal, which is the optical axis for MMP and CPL‐LSCM measurements. b) Absorbance and MMP CD spectra (solid lines, average of single crystals **d**, **e** and **a″**, low energy detector limit = 700 nm); PL and CPL spectra of microcrystals (dashed lines, *λ*_ex_ = 570 nm, low energy detector limit = 800 nm). c) Microscope image and their MMP CD map (*λ* = 404 nm) of single crystals **a–e** (16 elements MM and full Analytic Inversion calculation reported in Figure S3–24,25, respectively). d) (CPL)‐LSCM images of a single crystal (*λ*_ex_ = 570 nm, *λ*_em_ > 715 nm), with the left‐ (L − R) and right‐handed (R − L) CPL images showing the enantioselective differential chiral contrast (bottom).

The macrocycle binds the polycyclic aromatic hydrocarbon coronene (Figure 1), and so we sought to use molecular recognition to tune supramolecular structure and thus chiroptical properties in the solid‐state. L‐valinol macrocycle–coronene host–guest co‐crystals are grown by vapour diffusion,^[^ ^68^ ^]^ yielding air stable and purple, plate‐like single crystals up to 0.1 mm in size (Figure 4d). The crystals belong to the chiral monoclinic space group *P*2_1_, characterised by an additional two‐fold screw axis compared to *P*1. Crystal axes were again identified by comparison of morphology against data obtained by single crystal diffraction (Figure S1–4). Compared to the macrocycle alone, coronene binding changes the macrocycle's conformation and intermolecular packing (Figure 4a). Whilst coronene is encapsulated within the macrocycle's cavity (i.e., endocyclic binding, *d*_π–π _= 3.5 Å), the guest is also bound between macrocycles (i.e., exocyclic binding, *d*_π–π _= 3.5 Å), leading to two distinct coronene environments and a continuous intermolecular π‐stack along the long (*a*) axis (Figure 4a). This 1:2 host–guest stoichiometry is different to that seen in solution,^[^ ^68^ ^]^ where binding is exclusively endocyclic (1:1).

For analysis by MMP (Figure S3–24,35), the crystal plates were mounted on quartz slides using Fomblin Y and, as before, the plates were positioned flat against the slide (Figure 4c, left). This enabled consistent measurement along the short *c* axis of the crystals, caused by an absence of intermolecular π–π or hydrogen bonding interactions in this direction (Figure 4a). Relative to crystals of the macrocycle alone, co‐crystal absorption is red shifted (Δλ = 40 nm, Figure 4b), with an absorption edge at *λ* = ∼ 650 nm (bandgap = ∼ 1.9 eV, Figure S3–36). This redshift is indicative of charge‐transfer^[^ ^40^ ^]^ between the π‐electron poor macrocycle host and π‐electron rich coronene guest.^[^ ^90^ ^]^ Qualitative support was provided by DFT calculations on a host–guest model containing one macrocycle and two coronene units, taken from the co‐crystal structure, which show that the lowest energy transition is red shifted (by 55 nm) relative to the macrocycle alone (Supporting Table 8–1). The calculations also support the attribution of this shift to intermolecular charge‐transfer, due to the distribution of frontier molecular orbitals, with the HOMO and LUMO being mainly located on the coronene and PDI motifs, respectively (Figure 8–2).

Compared to the macrocycle crystals, the PDI's CD signal is red shifted in the co‐crystals, giving a CD at the absorption edge (Figure 4b), with a *g*_abs_ up to 9 × 10^−^^2^ at 644 nm (Table 1). A second CD signal is also observed at higher energy (*g*_abs_ up to 2 × 10^−^^2^ at *λ* = 406 nm, Figure 4b), which is not observed in the macrocycle‐only crystals (Figure 2b). This new signal is attributed to an induced CD from the achiral coronene guest within the chiral environment of the crystal, due to the signal being close to the absorption edge of coronene in the solid‐state (*λ* = ∼ 420 nm).^[^ ^90^ ^]^ It is notable that the higher‐energy CD signal was not observed from endocyclic‐only binding in solution, i.e., the 1:1 macrocycle–coronene complex,^[^ ^68^ ^]^ and so is exclusive to the 1:2 host–guest supramolecular structure obtained in co‐crystals (Figure 4a).

We mapped several of the host–guest single crystals (**a**‐**e**, see labels in Figure S3–25) at both the coronene‐based (Figures 4c and S3–24,25) and PDI‐based (Figure S3–26,27) CD regions, which demonstrated that the chiroptical properties are homogeneous, except in regions of twinning (e.g., crystal **d**). As seen for the macrocycle crystals, the CD is independent of the crystal orientation in the (*a*,*b*) plane since the manual rotation of crystal **a** by 90° did not change the CD sign or magnitude (Figures S3–28,30).^[^ ^91^ ^]^

Whilst the host–guest complex does not form thin films, drop casting a 1:2 macrocycle:coronene stoichiometric solution from toluene leads to the rapid formation of a purple microcrystalline material upon drying (Figure 5–8).^[^ ^92^ ^]^ The powder XRD and morphology of these microcrystals (up to 10 µm in size) shows a good agreement with that predicted for the analogous single crystals (Figure S6–1), thereby indicating a similar π‐stacked assembly, i.e., a coronene–macrocycle–coronene (donor–acceptor–donor) structure. The emergence and growth of these microcrystals was captured by microscopy on a glass slide, with illumination under cross‐polarised light confirming their crystalline nature (see Supporting Video and Figures S5–1,4). The π–π supramolecular assembly must be templated by the exocyclic‐bound coronene since lowering the host:guest stoichiometric ratio to 1:1 leads to distinct regions of the host–guest microcrystals and macrocycle‐only films (Figure S5–5,7).

Alongside emission from the residual “non‐assembled” macrocycle film on the slide (*λ* < 700 nm, Figures 4b and S4–2),^[^ ^93^ ^]^ the fluorescence spectrum of the host–guest microcrystals contains a new red‐shifted shoulder at *λ* = 780 nm (Figure 4b), consistent with PDI–coronene exciplex emission. The large Stokes shift of this emission band (Δ*λ* = 130 nm) is also evidence for the charge transfer nature of this exciplex and is in line with that of the acceptor–donor macrocycle–coronene complex in solution.^[^ ^68^ ^]^ CPL spectroscopy revealed that this exciplex emission yields strong CPL in the near‐infrared, *g*_lum_ = 7 × 10^−^^2^ at *λ* = 780 nm (Figure 4b and Table 1). The persistence of a relatively large *g*_lum_ in the co‐crystals may be explained by the charge transfer emission, which is linked to enhancements in CPL^[^ ^94^, ^95^ ^]^ due to an increase in the magnetic transition dipole moment.^[^ ^9^ ^]^

This strong, low energy CPL was confirmed by CPL microscopy of single‐ and micro‐crystals of the macrocycle–coronene complex (Figure 4d and S4–8,9), with both possessing relatively large (and matching^[^ ^96^ ^]^) *g*_EDCC_ values of 8 × 10^−^^2^ at *λ* > 715 nm, positioning them amongst the most red shifted organic crystalline CPL materials to date (Figure S1–1).^[^ ^18^ ^]^ This demonstrates the value of charge transfer interactions to tuning CPL into the near infrared whilst maintaining a high dissymmetry factor. Furthermore, the co‐crystal's broad and monosignate CPL is distinguished from the narrow and multisignate CPL fingerprint often exhibited by lanthanide materials, e.g., the predominantly magnetic dipole allowed (dJ4) transitions of Eu(III) complexes (*λ* = 680–710 nm).^[^ ^97^ ^]^ The broad and monosignate CPL demonstrated by these organic materials renders them well suited for applications in CPL microscopy^[^ ^59^ ^]^ and security^[^ ^98^ ^]^ as it eliminates the need for narrow bandpass filters due to the omission of CPL signal cancelling within the studied emission manifold, resulting in full recovery of CPL and harnessing maximised circular polarised brightness.^[^ ^87^ ^]^

## Summary and Conclusions

We have quantified the CD and CPL of single crystals composed of a bis‐PDI macrocycle dimer and its host–guest complex with coronene to understand how the chiroptical properties of organic dyes in solution might be translated to, and optimised in, solid‐state materials. Our focus is on single crystal materials since knowledge of chiral (supra)molecular structure can be connected to the sign, strength and energy of the chiroptical signal, establishing important mechanisms for tuning material properties.

This work demonstrates that the CD and CPL of chiral materials may be tuned via the non‐covalent interactions between organic chromophores. Notably, *g*_abs_ is larger (> 10^−^^2^) and the CD is more uniform in single crystals of the bis‐PDI macrocycle relative to thin films, likely due to superior homogeneity of the π–π dimer and its long‐range ordering in the chiral single crystal lattice. For the host–guest co‐crystals, exocyclic coronene binding templates macrocycle self‐assembly via intermolecular donor–acceptor π–π interactions. The significance of this is that charge transfer provides an effective strategy to red shifting chiroptical properties whilst maintaining high dissymmetry factors. Indeed, the macrocycle–coronene co‐crystals are notable for exhibiting strong CPL in the near infrared (*g*_lum_ = 7 × 10^−^^2^ at *λ* = 780 nm), a novelty for organic crystalline materials (Figure S1–1).

Due to their inherent anisotropy, quantitative analyses of solid‐state materials were performed using MMP and CPL‐LSCM techniques. From MMP, we uncovered differences in LD polarisation between crystals, which could limit the accuracy of CD spectra if collected on a conventional spectrometer. Likewise, we showed that CPL microscopy on appropriately sized and positioned single crystals can exclude contamination from reflected circularly polarised light, critical for validating bulk CPL measurements. From these studies, our recommendations for using MMP and CPL‐LSCM to analyse single crystals include: i) positioning crystals in a consistent orientation with respect to the optical axis, ii) avoiding twinned/cracked crystals for chiroptical homogeneity, iii) careful consideration of crystal thickness, due to potential consequences for signal‐to‐noise (MMP) and reflected light (CPL).

Overall, we believe that this work will inform the design of new chiral (supra)molecular structures, and their quantitative CD/CPL analysis in the solid‐state, to realise efficacious, tuneable and functional chiroptical materials.

## Supporting Information

The general methods, additional photophysical spectra, MMP maps, MMP spectra, all methods for processing and analysing the MMP data, CPL microscopy data and the supporting video can be found in the supporting information.

## Conflict of Interests

The authors declare no conflicts of interest.

## Supporting information



Supporting Information

Supporting Information

## Data Availability

The data that support the findings of this study are available in the Supporting Information of this article.
