# Supplementary material for: Tuning Circular Dichroism and Circularly Polarised Luminescence in Single Crystals of a Perylene Diimide Macrocycle
Source: Angew Chem Int Ed Engl. 2026 Feb 3;65(11):e20567. doi: 10.1002/anie.202520567 (PMC12970508; doi:10.1002/anie.202520567)
Supplement: Supplementary file 1 — Supporting Information [file ANIE-65-e20567-s002.pdf]

Supplementary Information

Tuning Circular Dichroism and Circularly Polarised Luminescence in Single Crystals of a Perylene Diimide Macrocycle

Denis Hartmann,[a] Samuel E. Penty,[b] Artemijs Krimovs,[b] Robert Pal,[b*] Tiberiu-M. Gianga,[c] Giuliano Siligardi,[c*] Timothy A. Barendt[a*]

[a]University of Birmingham, School of Chemistry, Edgbaston Campus, Birmingham, B15 2TT
[b]University of Durham, Department of Chemistry, Stockton Road, Durham, DH1 3LE
[c]Diamond Light Source, Harwell Science & Innovation Campus, Didcot, OX11 0DE

Contents

1) Chiroptial materials 2
2) Bulk photophysical properties 4
3) Mueller Matrix Polarimetry (MMP) 8
4) CPL measurements 31
5) Microscope images of macrocycle-coronene co-crystals........ 42
6) Powder X-ray diffraction 47
7) Atomic force microscopy 48
8) Computational Chemistry 49
9) References 51

1) Chiroptial materials

Summary of current organic crystalline material g-factors

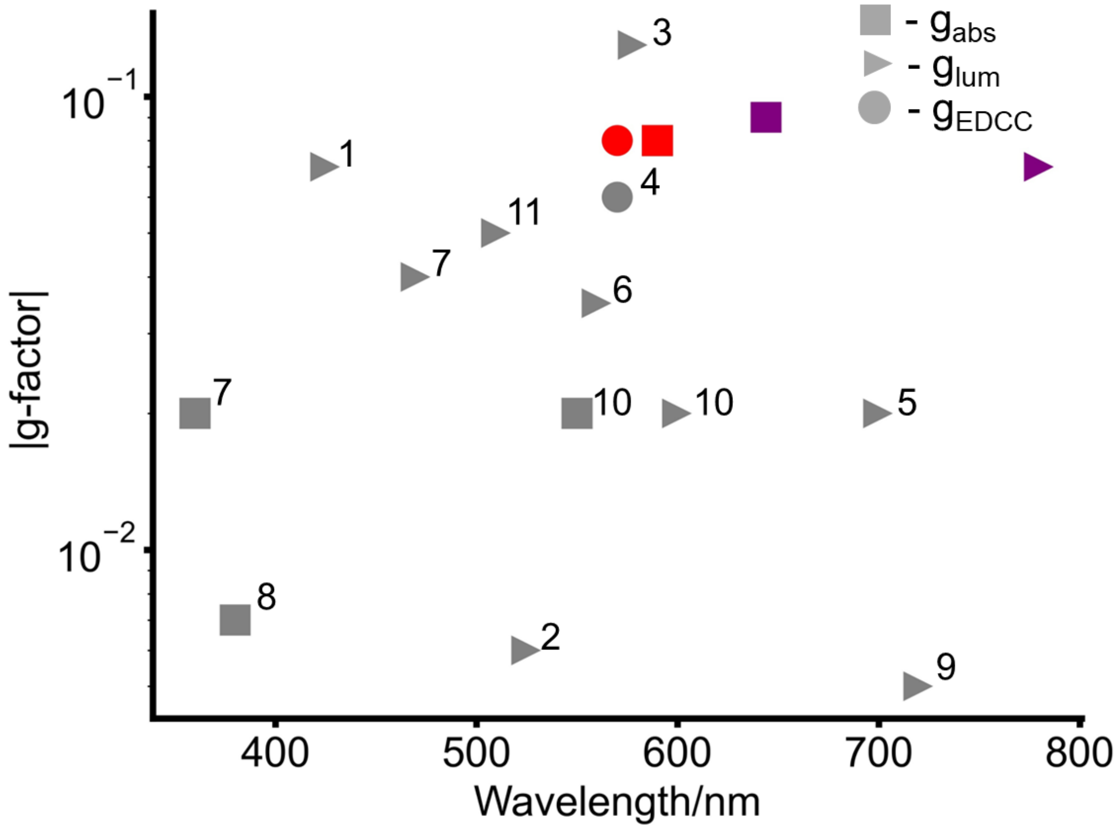

Supplementary Figure 1-1: Organic crystalline material dissymmetry factors (g).[1–11] Red/purple = this work. The g_{EDCC} is analogous to g_{lum}, being the enantioselective differential chiral contrast dissymmetry factor obtained by CPL-laser scanning confocal microscopy.

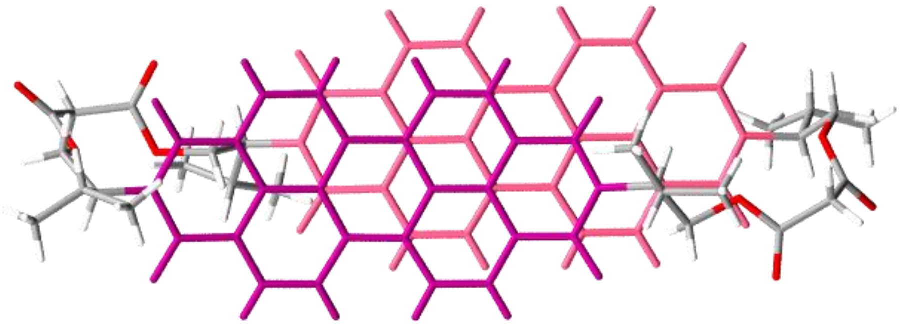

Supplementary Figure 1-2: Co-linearity of the two PDI chromophores (purple and pink) in the single crystal structure of the chiral macrocycle.

Thin film preparation

Thin films of the L or D-chiral macrocycle were prepared through drop casting of a solution in toluene (5 mM) onto quartz glass substrates. The film of L-valinol-based macrocycle chosen for chiroptical measurements was 120 nm thick, as measured by AFM (**Supp. Fig. 7-1**). Racemic films were prepared by mixing solutions of both L- and D-valinol-macrocycle (5 mM in PhMe) in equal ratio before dropcasting.

Determination of crystal axes by single crystal X-ray diffraction

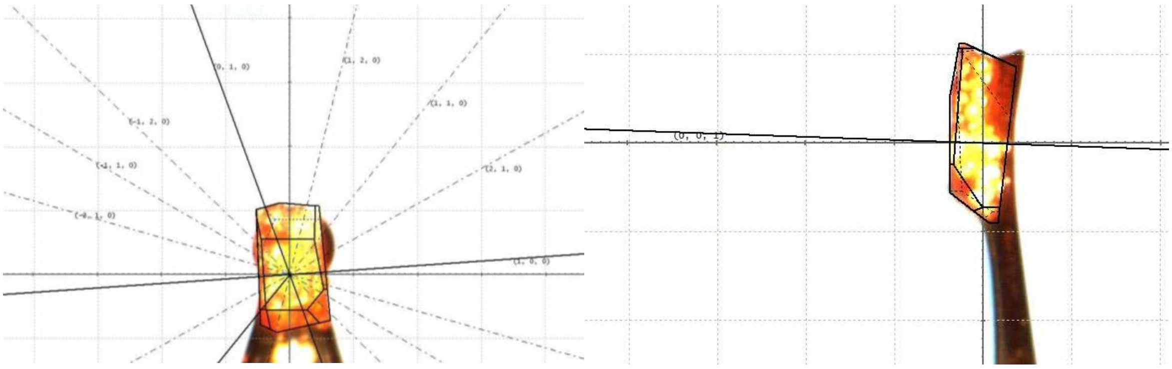

Supplementary Figure 1-3: Single crystal of the macrocycle and its corresponding (h, k, l) values as recorded by the diffractometer (Rigaku XtaLAB Synergy-S) and visualised in CrysAlis Pro.

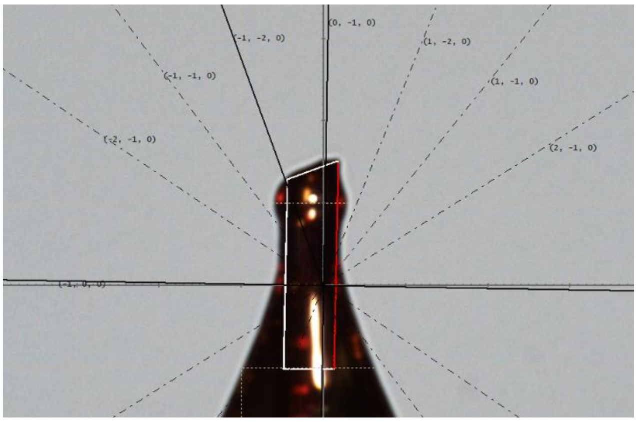

Supplementary Figure 1-4: Single crystal of the host/guest crystal and its corresponding (h, k, l) values as recorded by the diffractometer (Synergy Custom four-circle diffractometer equipped with a HyPix-Arc 100 detector) and visualised in CrysAlis pro.

The a/b/c-axes of both macrocycle crystals and host-guest co-crystals were determined using Mercury software. Axes of measured crystals were assigned by comparison of morphology with the crystals recorded by single crystal x-ray diffraction. In all cases, the short c-axis was easily identifiable. The long a-axis was found to be in the direction of most intermolecular interaction (along the π-stacking direction) and therefore was assigned as the longest axis of an individual crystal.

2) Bulk photophysical properties

Absorbance measurements of thin films were recorded on a Shimadzu UV-3600i Plus Spectrometer equipped with a thin film holder. The Shimadzu UV-3600i plus has a wavelength accuracy of ± 0.2 nm in the visible and UV region, a baseline flatness of ± 0.002 nm (200 – 3000 nm) and a noise level of <0.00008 Abs (900 nm).

Diffuse reflectance spectra were recorded on a Shimadzu UV-3600i Plus Spectrometer equipped with an integrating sphere. Absorbance spectra were then obtained using a Kubelka-Mulk transformation of the total reflection via the built-in software.

Circular Dichroism measurements were taken on a JASCO J-1500 CD Spectrophotometer. Recorded traces were then baseline corrected and smoothed using the provided software.

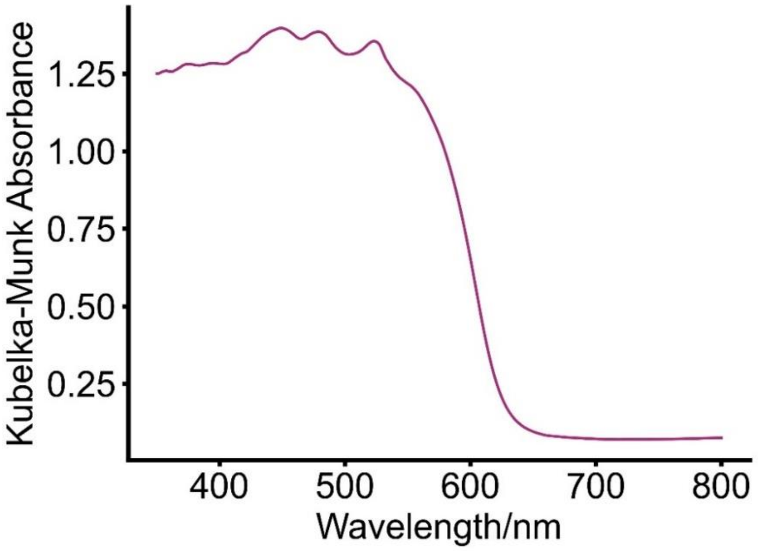

Supplementary Figure 2-1: Reflectance measurement of single crystals of the macrocycle, processed using the Kubelka-Munk transform.

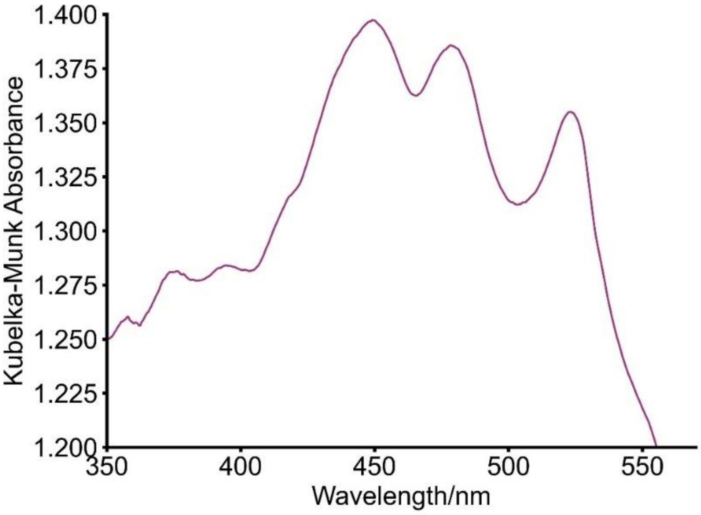

Supplementary Figure 2-2: Zoom of the area from 350-550 nm of **Supplementary Figure 2-1**, showing vibronic bands arising from the PDI.

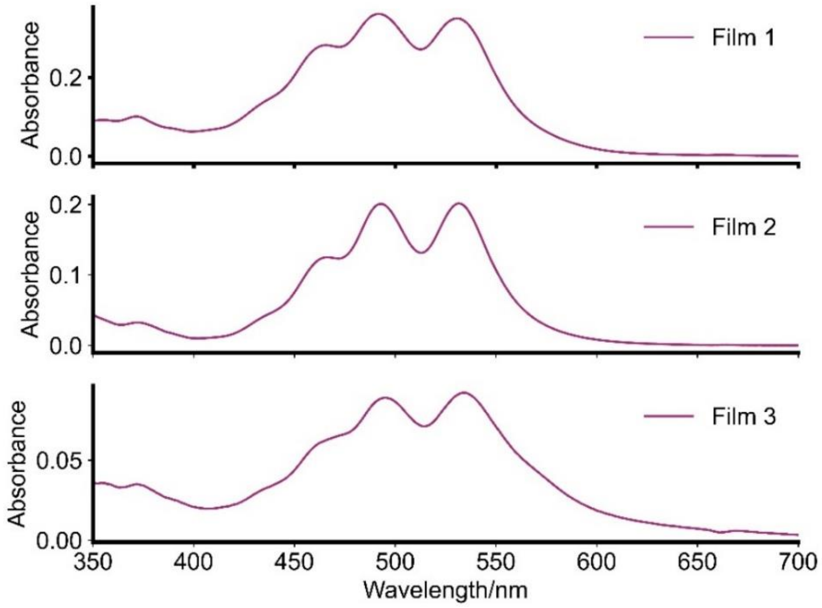

Supplementary Figure 2-3: Absorbance spectra of three macrocycle films (**1-3**). Variations in the 0-0/0-1 ratio are observed, due to different ratios of 'open':'closed' macrocycle conformers in the film. For quantitative comparison against single crystals, film **1** was taken forward for MMP analysis since it gave the strongest CD.

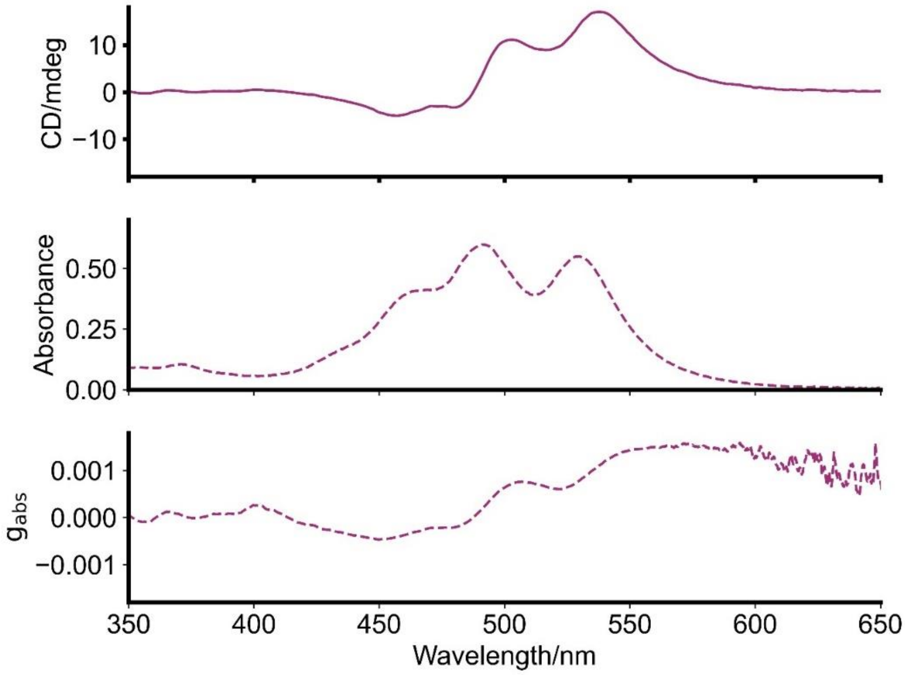

Supplementary Figure 2-4: CD, absorbance and g_{abs} of L-valinol macrocycle thin film **1** (**Supplementary Figure 2-3**) as recorded on a conventional benchtop CD spectrometer.

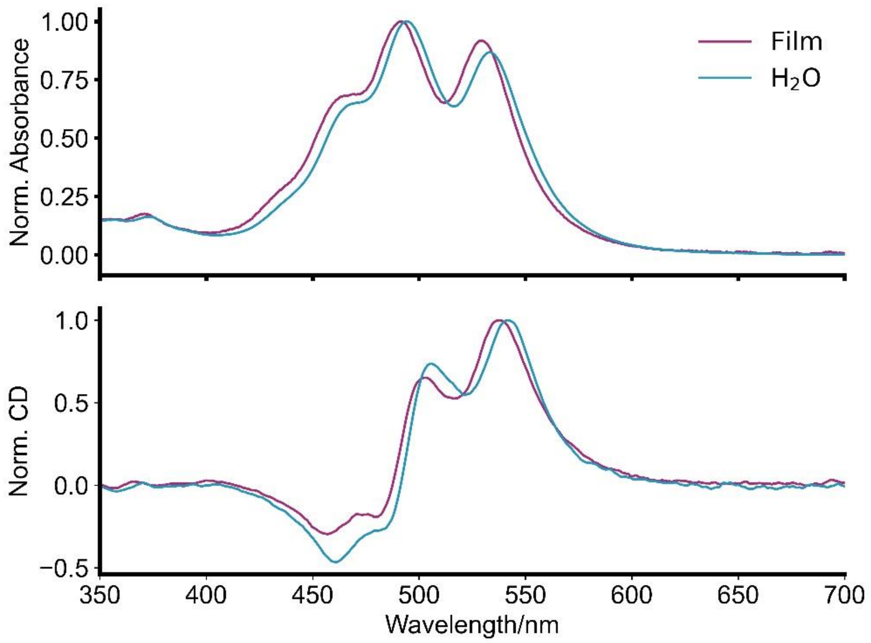

Supplementary Figure 2-5: Comparison of absorbance and CD spectra of thin film **1** with analogous solution spectra of the macrocycle, which is discrete in both solutions, i.e., no intermolecular aggregation.[12] The close agreement between the thin film and H_2O solution spectra indicates a similar situation in films and aqueous media, i.e., the macrocycle exhibits H-type intramolecular aggregation due to (major) population of the 'closed' *P*-helical conformation.

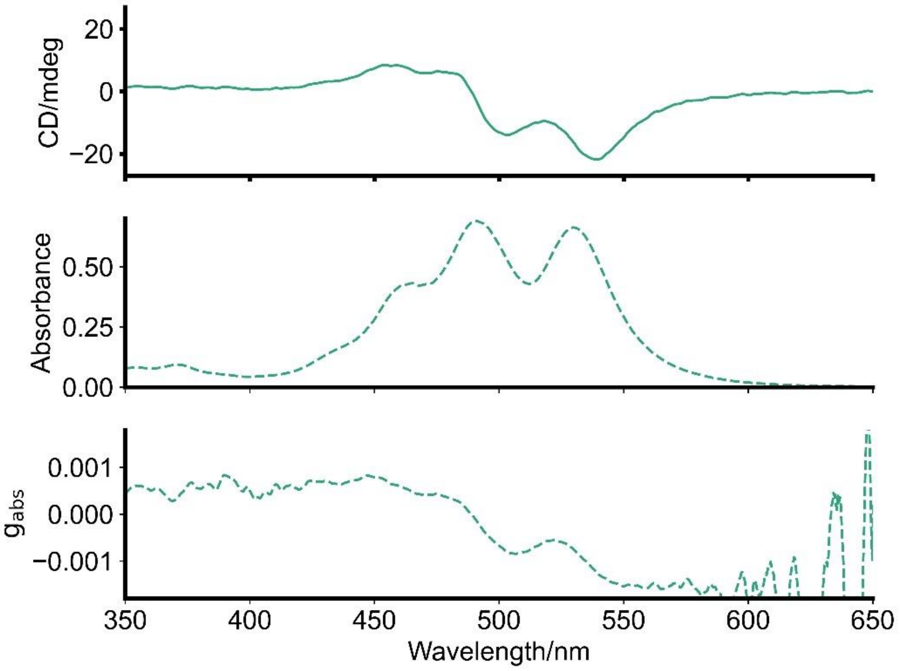

Supplementary Figure 2-6: CD, absorbance and g_{abs} of a D-valinol macrocycle thin film as recorded on a conventional benchtop CD spectrometer.

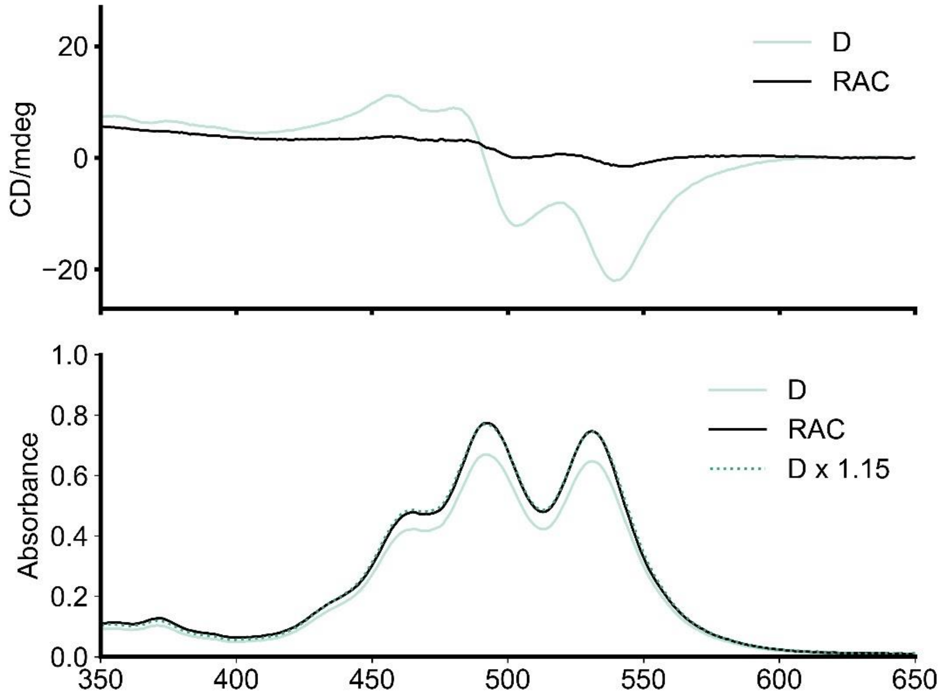

Supplementary Figure 2-7: CD and absorbance spectra of racemic (RAC) macrocycle thin films, with D-valinol macrocycle film (D) overlaid. From scaling the absorbance of the D-valinol macrocycle-based films, we were able to estimate a 5% excess of D- over L-valinol-based macrocycle in the racemic film, arising from the manual mixing of enantiomers.

3) Mueller Matrix Polarimetry (MMP)

Methods for MMP measurements at B23 at Diamond Light Source

Circular dichroism CD is the differential absorption of left and right circularly polarised light by a chiral molecule. Linear dichroism (LD) and linear birefringence (LB) being orders of magnitude greater than CD, and the circular birefringence of the sample, affect substantially the spectral shape, magnitude, intensity and sign of the CD measured with benchtop and synchrotron CD instruments. Also, the residual static strain birefringence of the photoelastic modulator and polarizing prism can generate CD artifacts. In this case it is an apparent CD that is measured. For these reasons, an accurate chiroptical characterisation of solid-state chiral materials is only achievable using MMP. From MMP data each optical property can be decomposed from the others.[13,14]

The custom-built B23 MMP by Hinds Instruments is composed of four photoelastic modulators in transmission operating at different frequencies, two in the polarization state generator and two in the polarization state analyser[15,16] coupled to a double grating subtractive monochromator (Olis Instruments).[17] The wavelength range of B23 MMP is 190-700nm, which has been successfully employed to measure and quantify the CD of a wide range of chiral materials in the solid-state.[18–23] With benchtop CD instruments, the smallest area that can be measured is about 3 mm in diameter giving an average CD compared to 50 µm using the MMP instrument at B23.[15]

The visual inspection of the differential 16 Mueller Matrix elements (for example, **Supplementary Figures 3-1** and **3-7**) reveals whether M30 and M03 (the assigned CD elements) are symmetric or antisymmetric. For the latter, it means the sample is bianisotropic having both linear and circular anisotropy components.[24] An important measurement derived from MMP is the depolarization index[25] (DI) that varies from 1 for non-depolarising materials to zero for depolarising materials.

In general, two methods can be used to calculate the six elementary polarization properties, namely CD (circular dichroism); LD (linear dichroism along the x-y axes); LD' (along the ±45° axes); CB (circular birefringence); LB (linear birefringence along the x-y axes); and LB' (along the ±45° axes). For non-depolarizing materials, the natural logarithm Matrix[26] method is capable of decomposing the polarization elements CD, LD, LD', CB, LB and LB'. For materials with a depolarization index between 1 and 0.5, the Analytic Inversion method[27] can decompose the polarization properties.

The Analytic Inversion is the method used in this study, enabling the CD to be decomposed from the other optical polarizations. Based on the type of materials investigated, some areas or spectral regions may show DI > 1 (due to e.g., light scattering, iridescence and reflections), in which case our interpretation of the data is only carried out in the areas of spectral regions with 0.5 ≥ DI ≤ 1, since only these will have a physical meaning. A DI < 0.5 is indicative of complex materials for which none of the current methods can decompose their optical properties.

Crystals were mounted onto quartz slides through suspension in Fomblin Y (LVAC25/6, average mol. Wt. 3300), preventing the crystals from moving and drying out too quickly. The fomblin background was always subtracted from MMP measurements on crystals. Crystals were located using the moving sample stage by mapping their absorbance. Crystal absorbance spectra were corrected for light scattering by applying a linear baseline correction using the a|e - UV-Vis-IR Spectral Software by FluorTools. Errors in g_{abs} were estimated by fitting gaussian functions to the positive peaks in the region of ~650 nm of co-crystals d, e and a'' and calculating the standard fitting error, giving an estimate of the noise obtained and

therefore of the variability in g_{abs}. The 16 elements of the 4 × 4 Mueller Matrix (spectroscopic and mapping data) are presented for each sample. All datasets were processed with the Analytic Inversion method (for DI between 0.5 and 1) calculating the CD, CB, LD, LD', LB, and LB' optical properties and presented together with the absorbance (Abs), the dissymmetric factor (g_{abs} = ΔA/A), and the Depolarization Index (DI) to allow for comparison between different samples. The Fomblin Y background (**Supplementary Figures 3-2 and 3-3**) has been subtracted for all MMP data sets of single crystals. We note that crystal absorption at the wavelength of maximum CD is non-zero (e.g., **Supplementary Figure 3-7**), indicating that differential light scattering is not an issue in MMP measurements.

M_{00} T	M_{01} -LD	M_{02} -LD'	M_{03} CD
M_{10} -LD	M_{11} T	M_{12} CB	M_{13} LB'
M_{20} -LD'	M_{21} -CB	M_{22} T	M_{23} -LB
M_{30} CD	M_{31} -LB'	M_{32} LB	M_{33} T

Supplementary Figure 3-1: Optical properties assigned to the 16 elements of the 4x4 Mueller matrix (MM). The principal diagonal represents the Transmission of the investigated thin film. The green elements CD, LD (along 0° and 90°axes) and LD' (along ±45° axes) are symmetric whereas the red elements CB, LB and LB', and blue elements -CB, -LB and -LB' are antisymmetric.

MMP Spectra of Fomblin Y

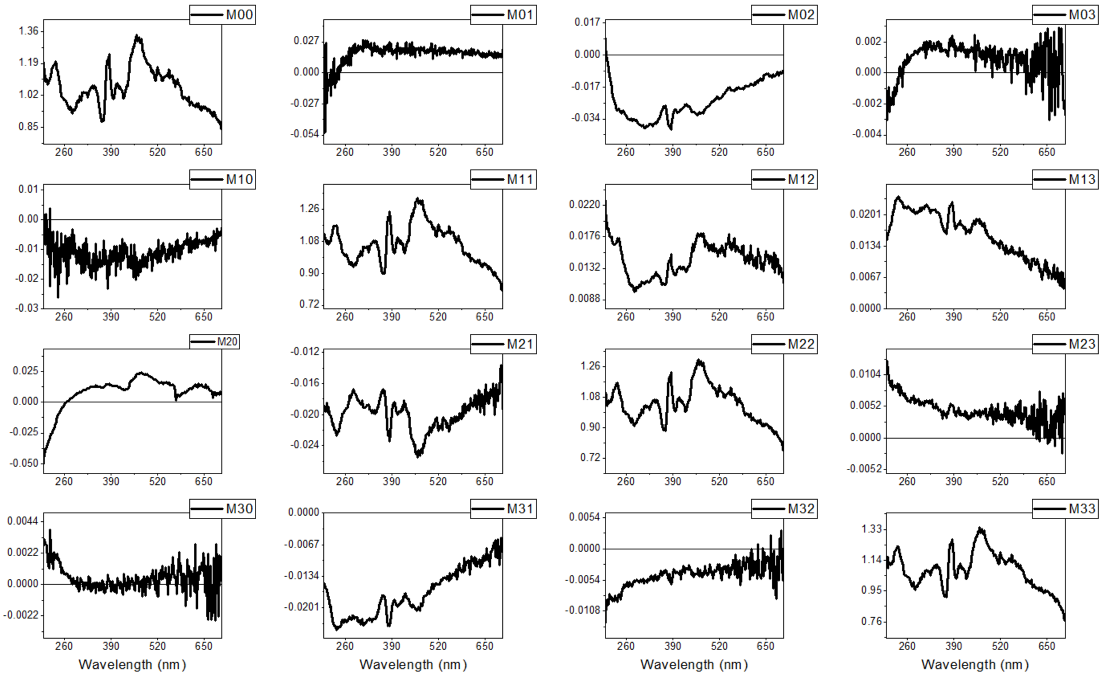

Supplementary Figure 3-2: Raw data of 4x4 Mueller-Matrix of Fomblin Y recorded by the MMP over the range of 250-700 nm.

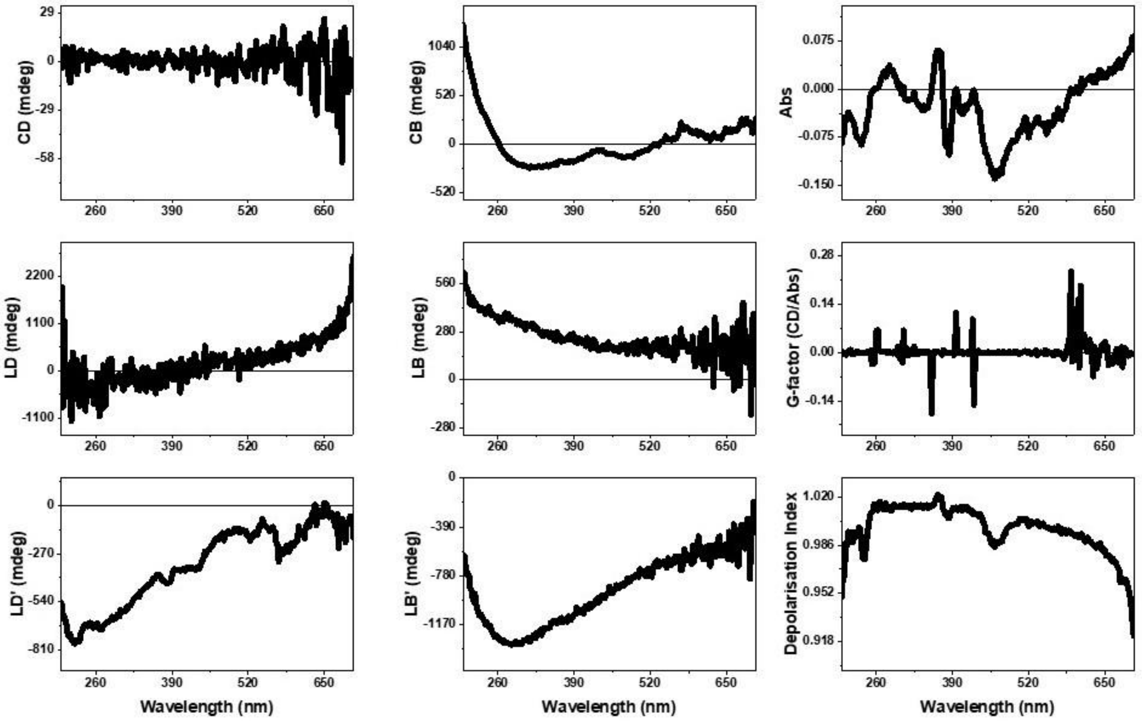

Supplementary Figure 3-3: Chiroptical properties of Fomblin Y used to hold crystals in place as recorded by MMP and processed using the analytic inversion method.

Macrocycle single crystal measurements
Measurement of single crystal 1 by MMP

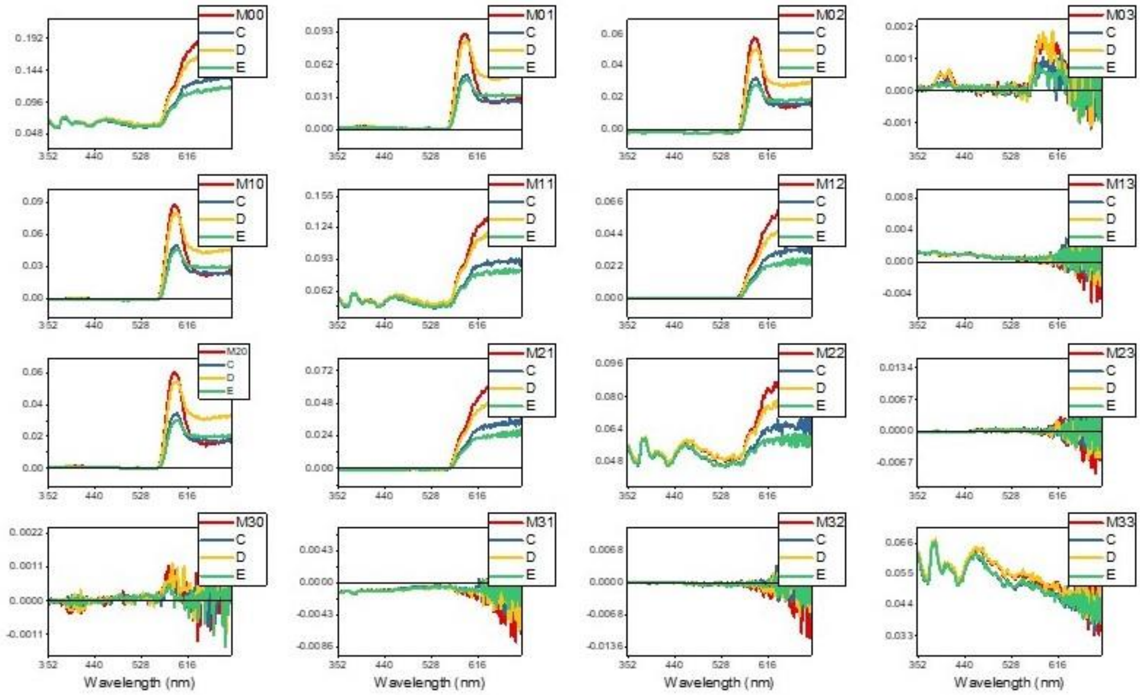

Supplementary Figure 3-4: 4x4 Mueller-Matrix of macrocycle single crystal **1** at 4 positions recorded by MMP.

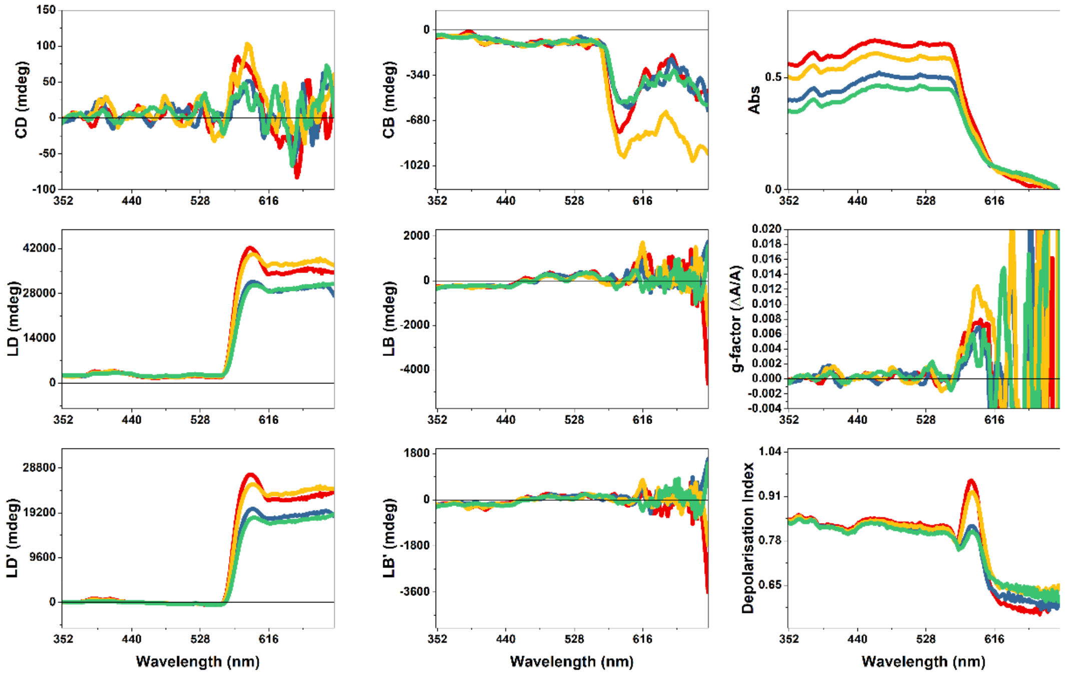

Supplementary Figure 3-5: CD, CB, Abs, LD, LB, g-factor, LD', LB' and DI spectra at 4 positions of macrocycle single crystal **1** with subtracted spectrum of fomblin (**Supp. Fig. 3-3**), as recorded by MMP and processed with the Analytic Inversion method. The g_{abs} values at wavelengths > 616 nm are neglected due to noise.

Measurement of Single Crystal 2 by MMP

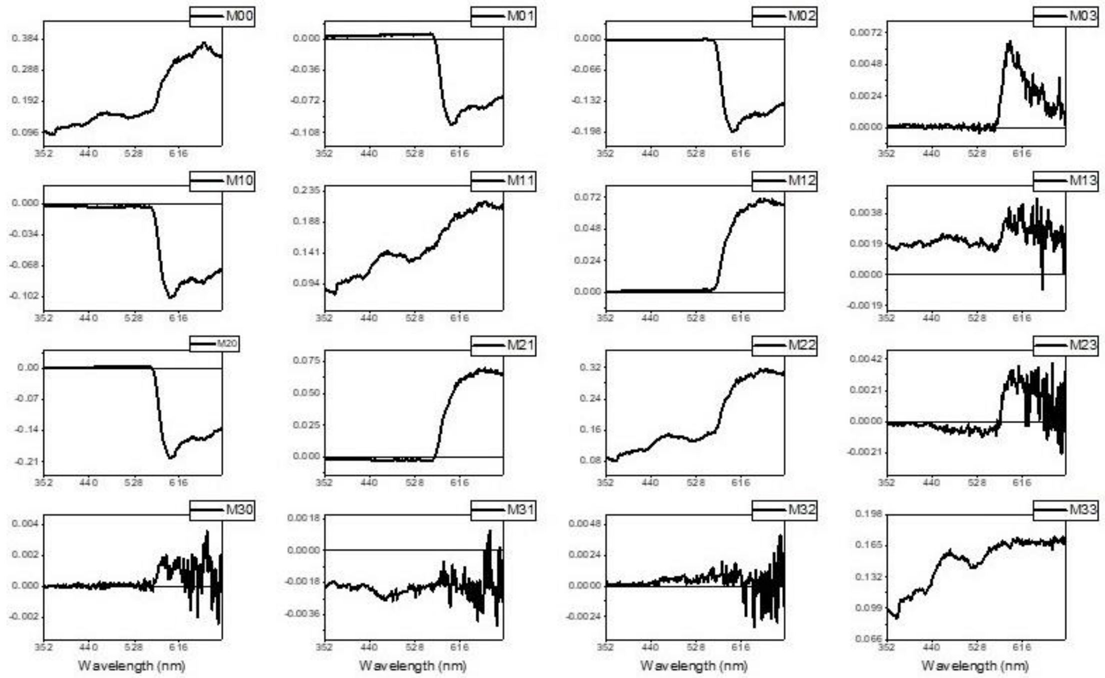

Supplementary Figure 3-6: Raw data of 4x4 Mueller-Matrix of macrocycle single crystal **2** with subtracted MMP spectrum of fomblin on fused silica (12x12x1.5mm window) (**Supp. Fig. 3-3**).

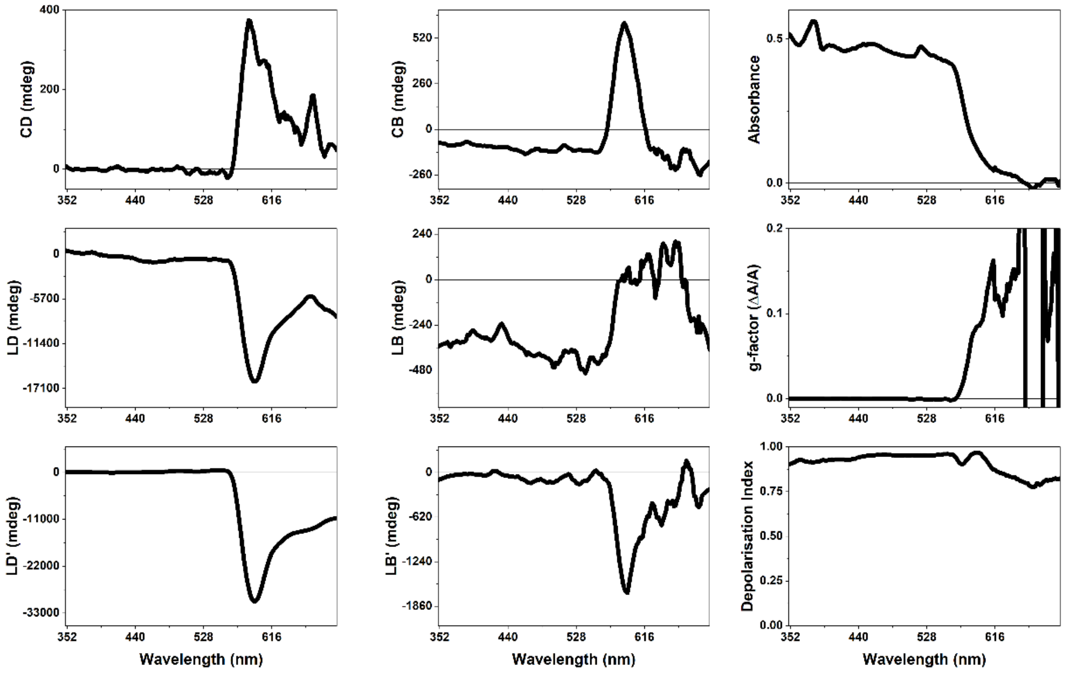

Supplementary Figure 3-7: CD, CB, Abs, LD, LB, g-factor, LD', LB' and DI spectra of macrocycle single crystal **2** from MMP data with subtracted MMP spectrum of fomblin on fused silica (12x12x1.5mm window) (**Supp. Fig. 3-3**) and processed with the Analytic Inversion method. The g_{abs} at wavelengths > 616 nm are neglected due to noise.

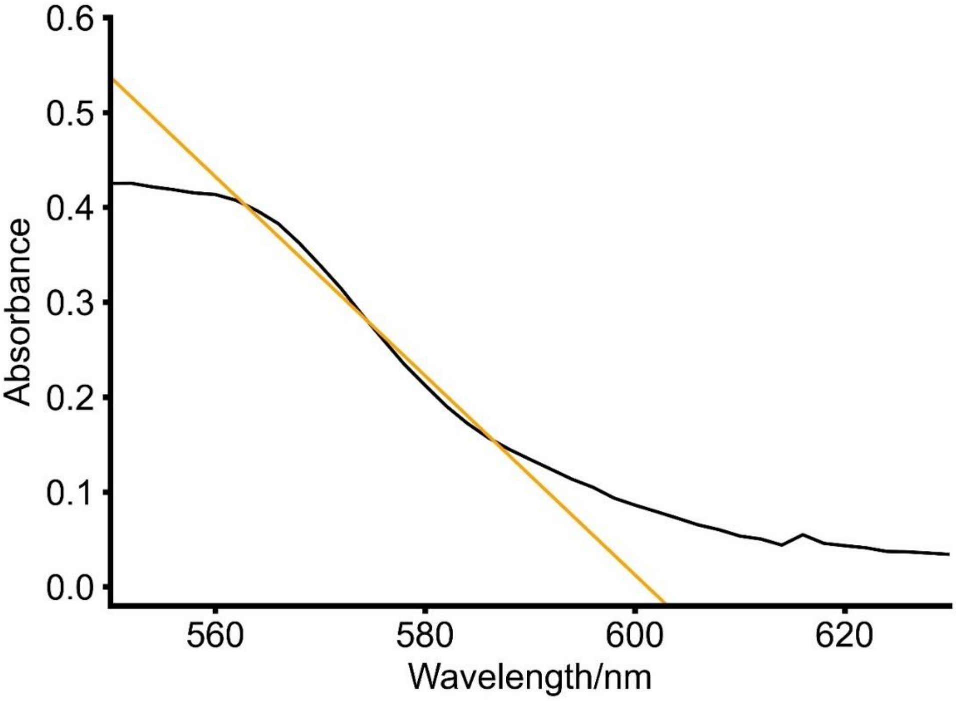

Supplementary Figure 3-8: Determination of bandgap of macrocycle crystal 2 from its absorbance spectrum recorded by MMP. A trendline was fitted for the datapoints from 570 nm to 590 nm, resulting in an x-intercept at ~600 nm, which corresponds to a photon energy of 2.07 eV.

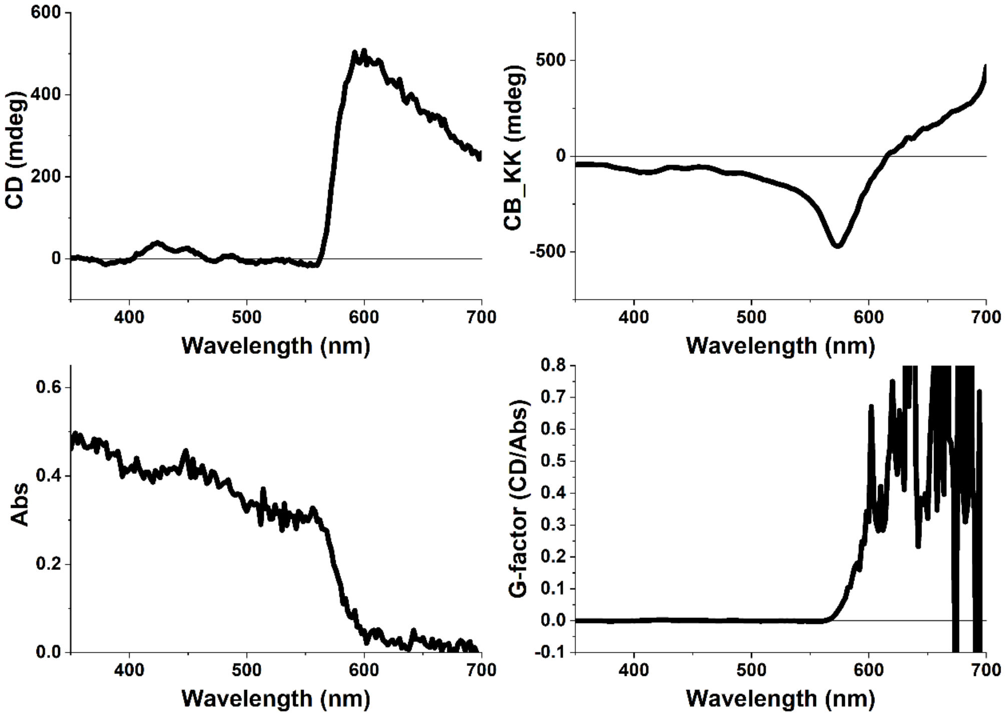

Supplementary Figure 3-9: Spectra of macrocycle single crystal **2** measured with the MMP instrument operating in CD mode (akin to a benchtop CD instrument) and baseline corrected by subtraction of the fomblin background on fused silica recorded in CD mode. The g_{abs} at wavelengths > 600 nm are neglected due to noise.

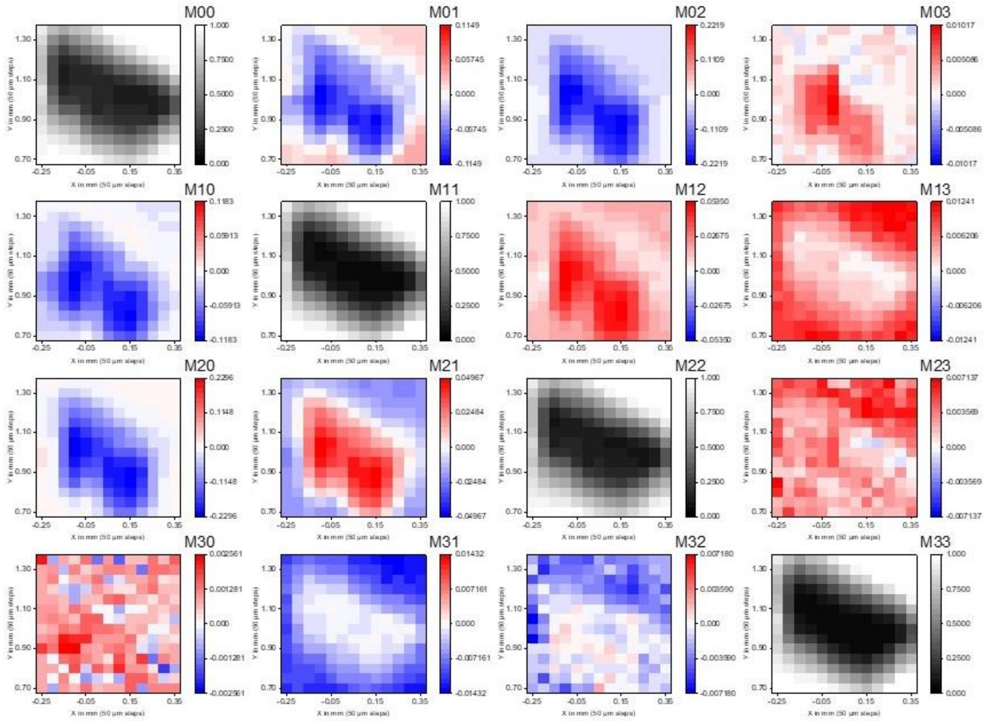

Supplementary Figure 3-10: Differential 4x4 Mueller-Matrix at 590 nm of macrocycle single crystal **1**.

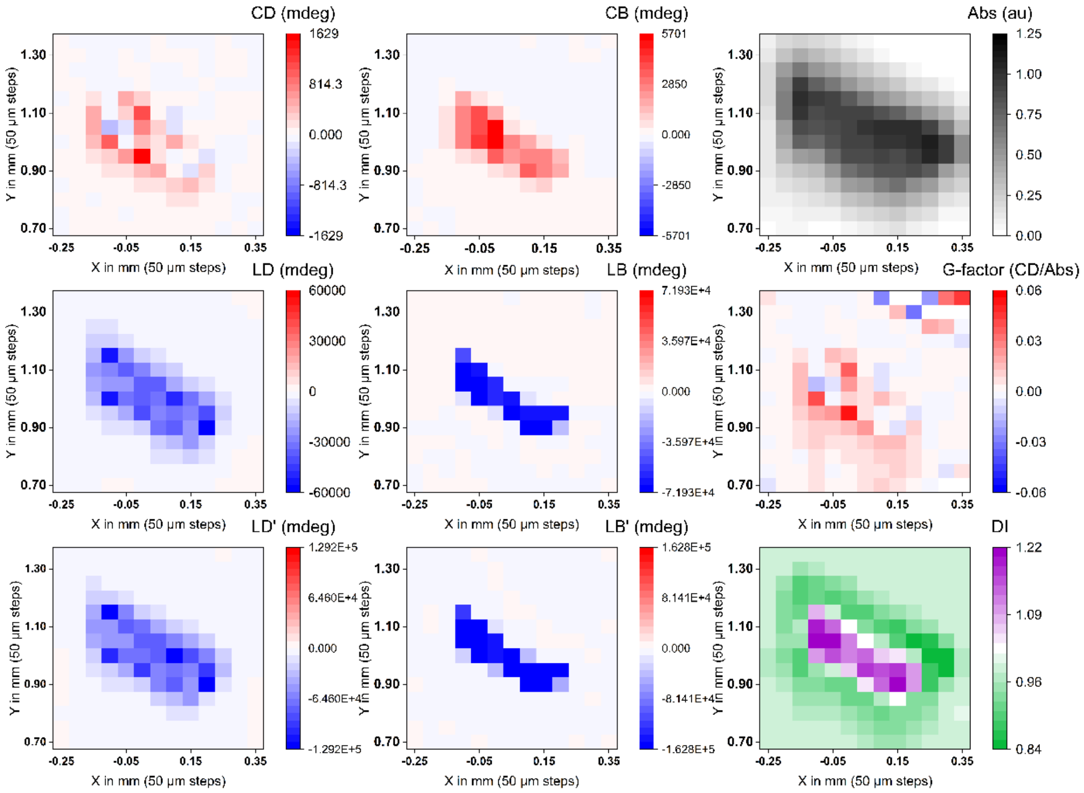

Supplementary Figure 3-11: CD, CB, Abs, LD, LB, g-factor, LD', LB' and DI Map recorded at 590 nm of macrocycle single crystal **1** with fomblin background subtracted and processed with the Analytic Inversion method.

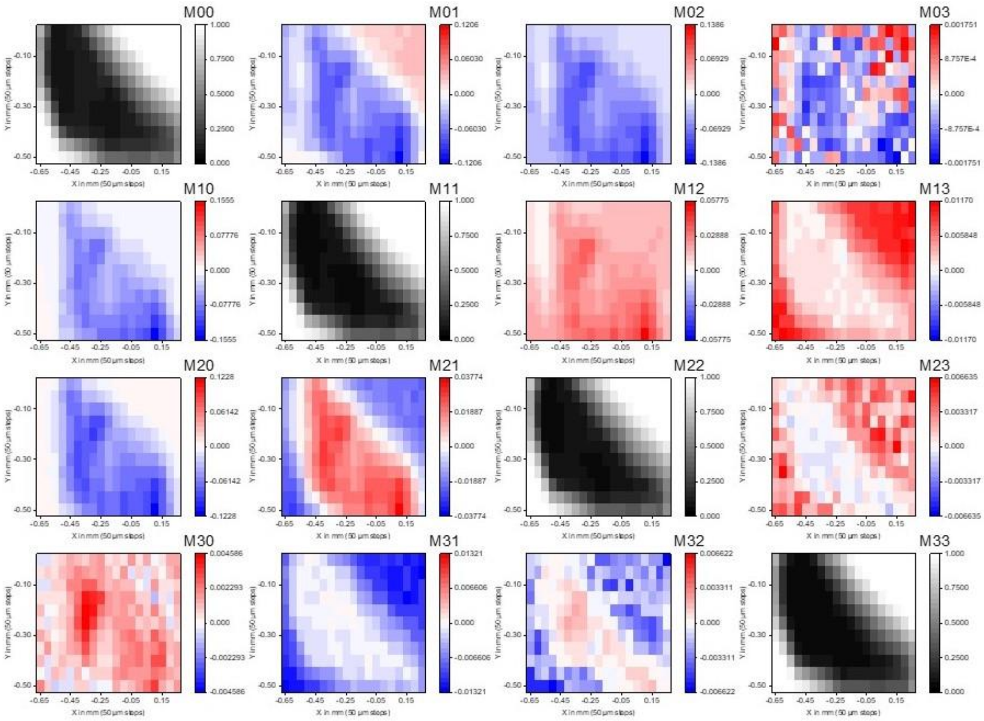

Supplementary Figure 3-12: Raw differential 4x4 Mueller-Matrix at 590 nm of macrocycle single crystal **2**.

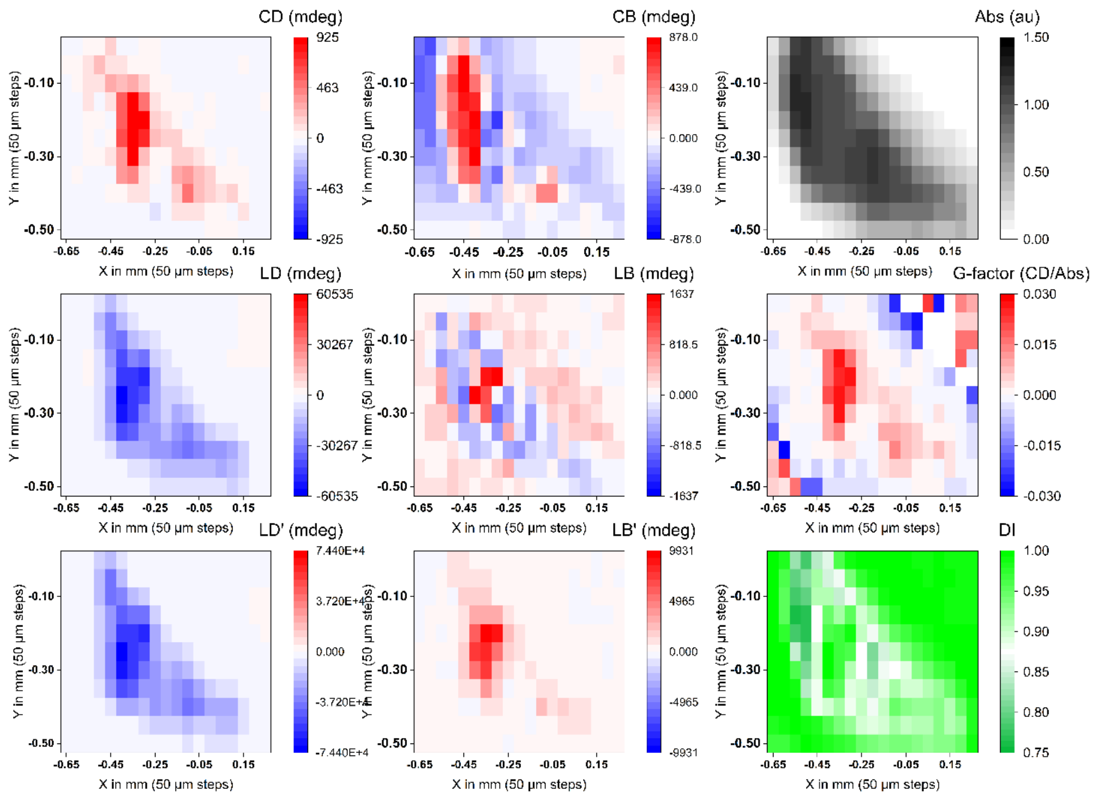

Supplementary Figure 3-13: CD, CB, Abs, LD, LB, g-factor, LD', LB' and DI Map recorded at 590 nm of macrocycle single crystal **2** with fomblin background subtracted and processed with the Analytic Inversion method.

Measurement of Single Crystal 3 by MMP

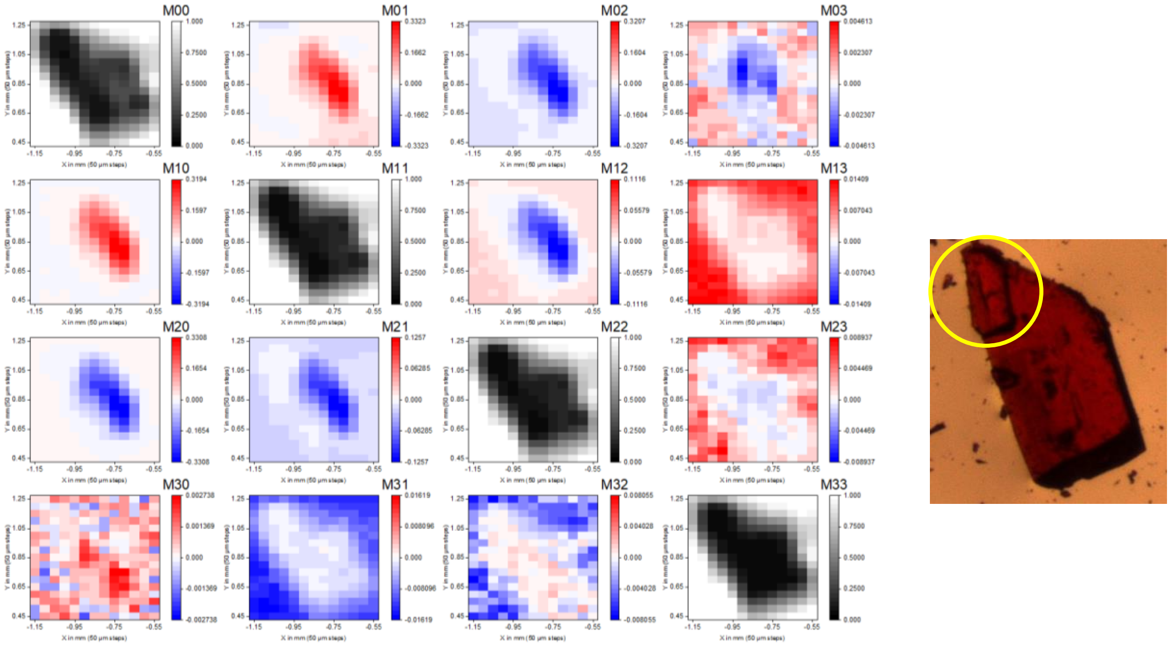

Supplementary Figure 3-14: Raw data of differential 4x4 Mueller matrix for macrocycle single crystal **3** containing a twinned region (highlighted with yellow circle) measured at 590 nm.

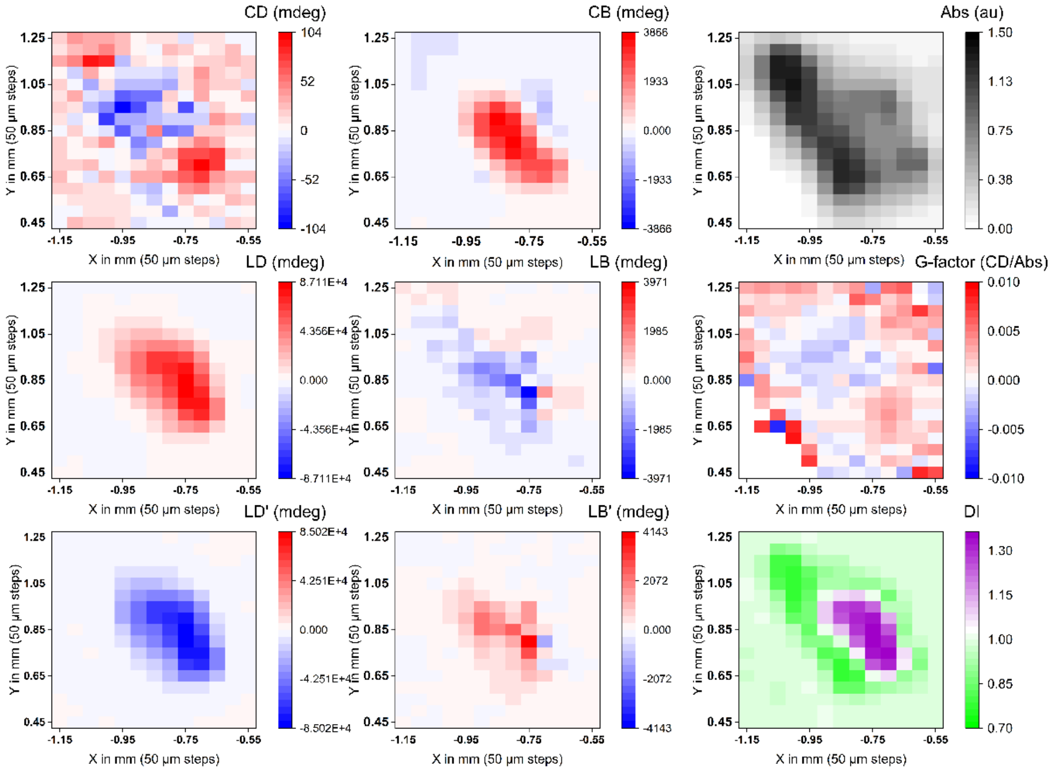

Supplementary Figure 3-15: CD, CB, Abs, LD, LB, g-factor, LD’, LB’ and DI Map recorded at 590 nm of macrocycle single crystal **3** (including twinned region) with fomblin background subtracted and processed with the Analytic Inversion method.

Macrocycle thin film measurements

MMP mapping was performed at λ = 546 nm, chosen because this is the wavelength at which the *M*- and *P*-helical conformers of the bis-PDI macrocycle have opposite CD.[12]

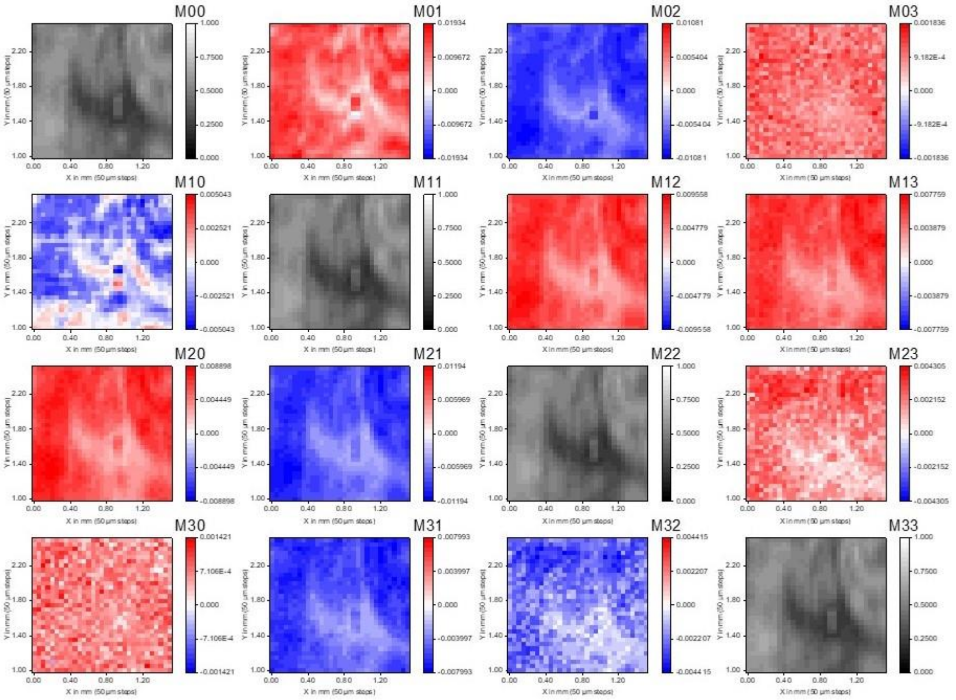

Supplementary Figure 3-16: Raw data of differential 4x4 Mueller-Matrix of L-valinol macrocycle thin film **1**.

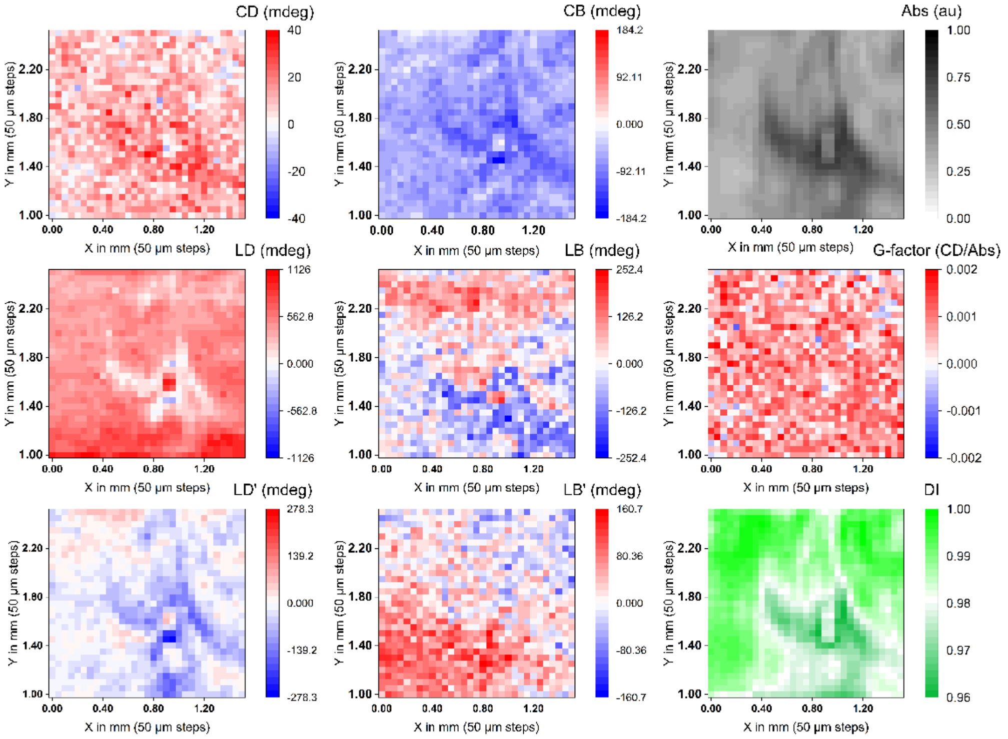

Supplementary Figure 3-17: MMP map of L-valinol macrocycle thin film **1** at 31 x 31 steps of 50 µM recorded at 546 nm, baseline corrected for the glass substrate and processed using the Analytic Inversion method.

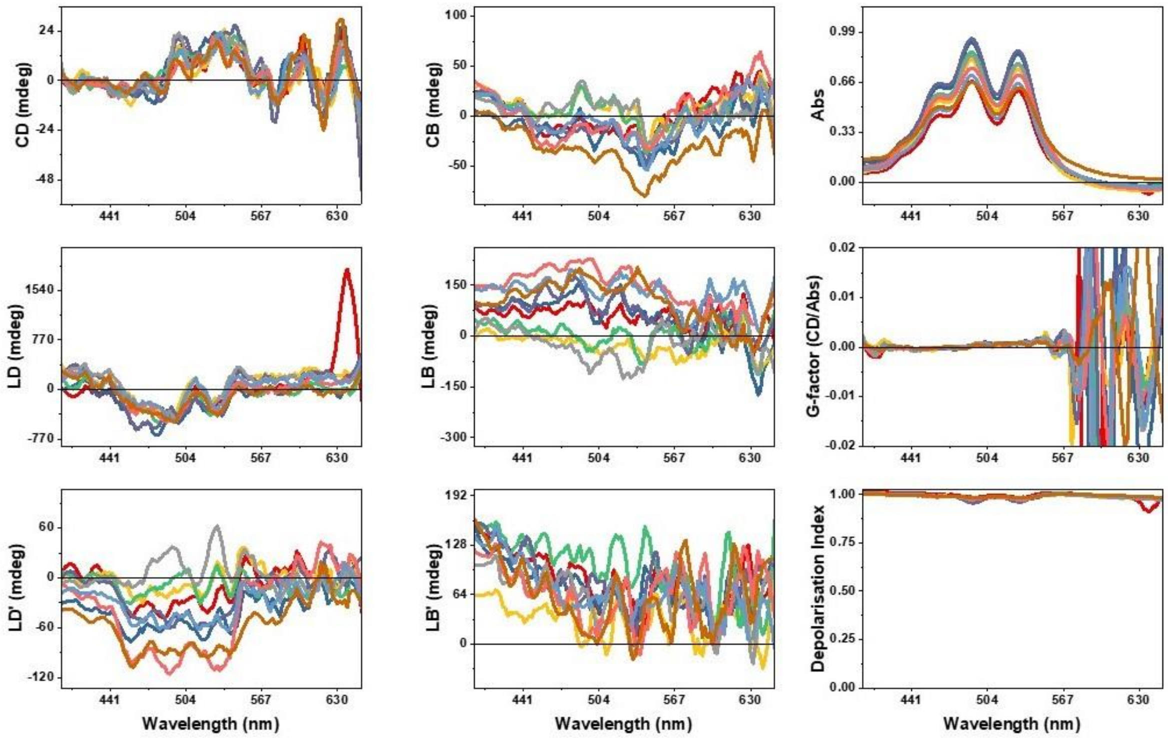

Supplementary Figure 3-18: CD, CB, Abs, LD, LB, g-factor, LD', LB' and DI spectra of L-valinol macrocycle thin film **1** recorded at 9 different positions on the film processed with Analytic Inversion method. The g_{abs} at wavelengths > 580 nm are neglected due to noise.

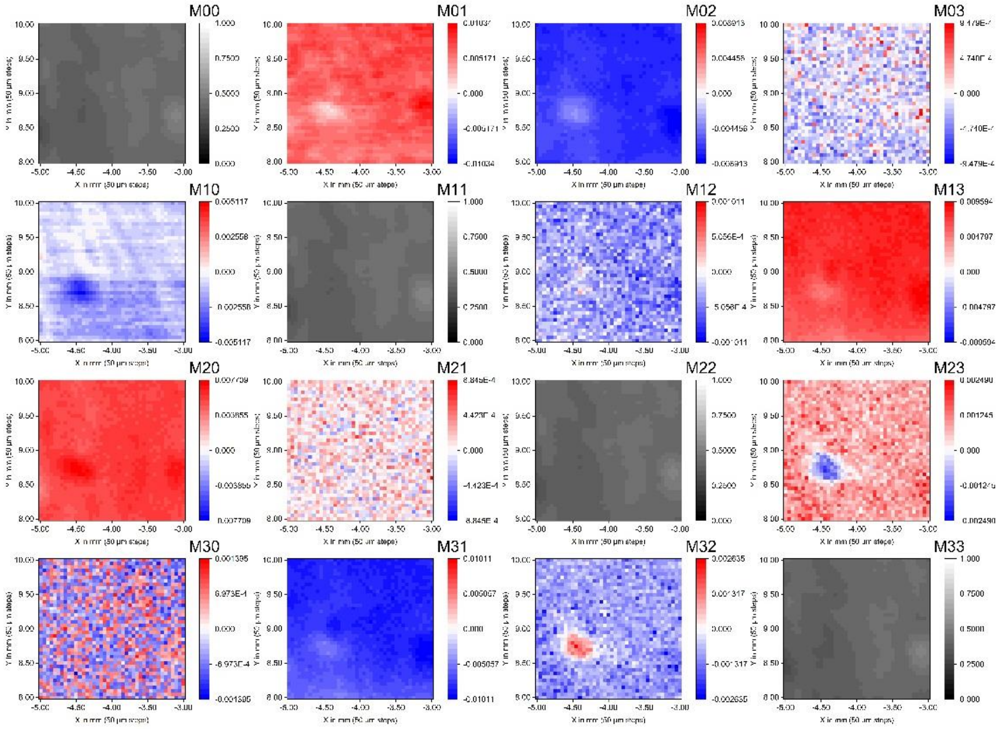

Supplementary Figure 3-19: Raw data of differential 4x4 Mueller-Matrix of D-valinol macrocycle thin film.

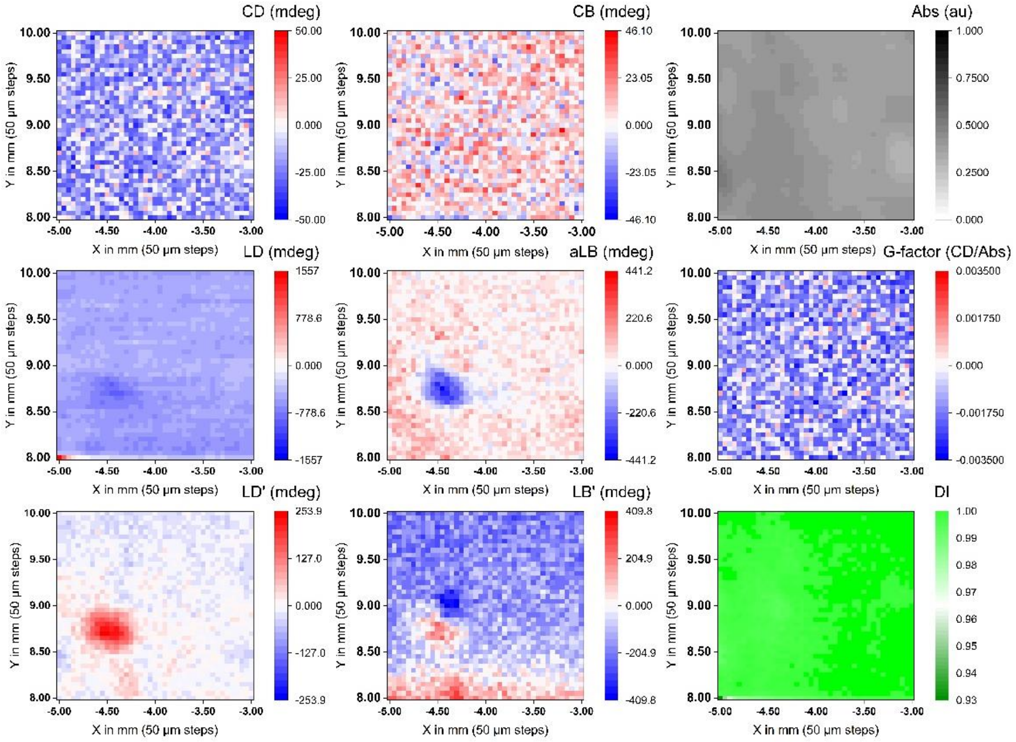

Supplementary Figure 3-20: MMP map of D-valinol macrocycle thin film **1** at 41 x 41 steps of 50 µM recorded at 546 nm, baseline corrected for the glass substrate and processed using the Analytic Inversion method.

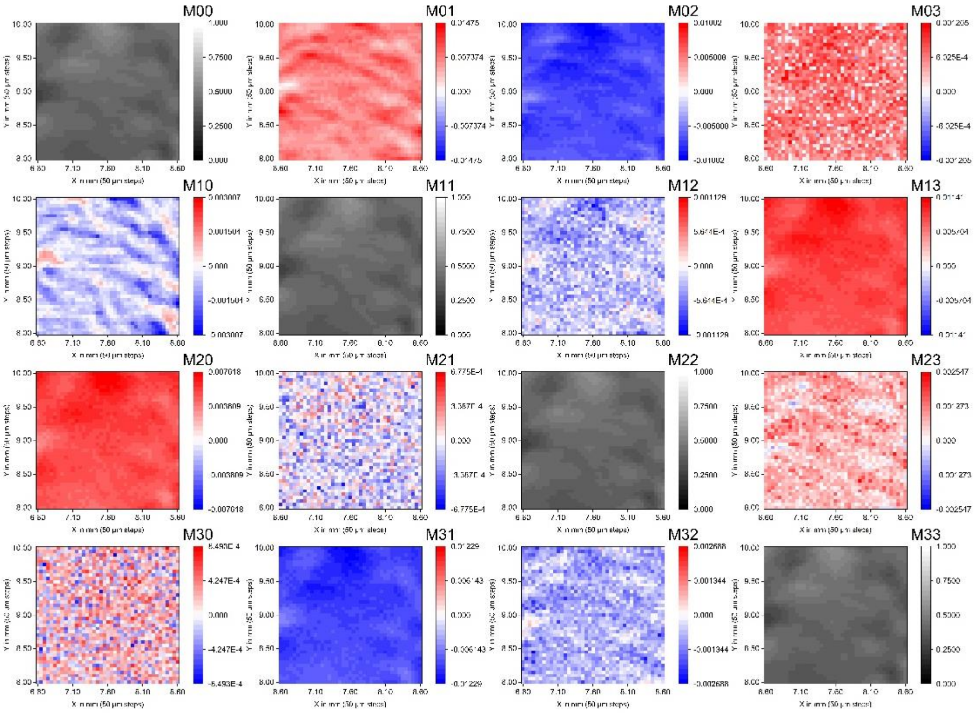

Supplementary Figure 3-21: Raw data of differential 4x4 Mueller-Matrix of racemic valinol macrocycle thin film.

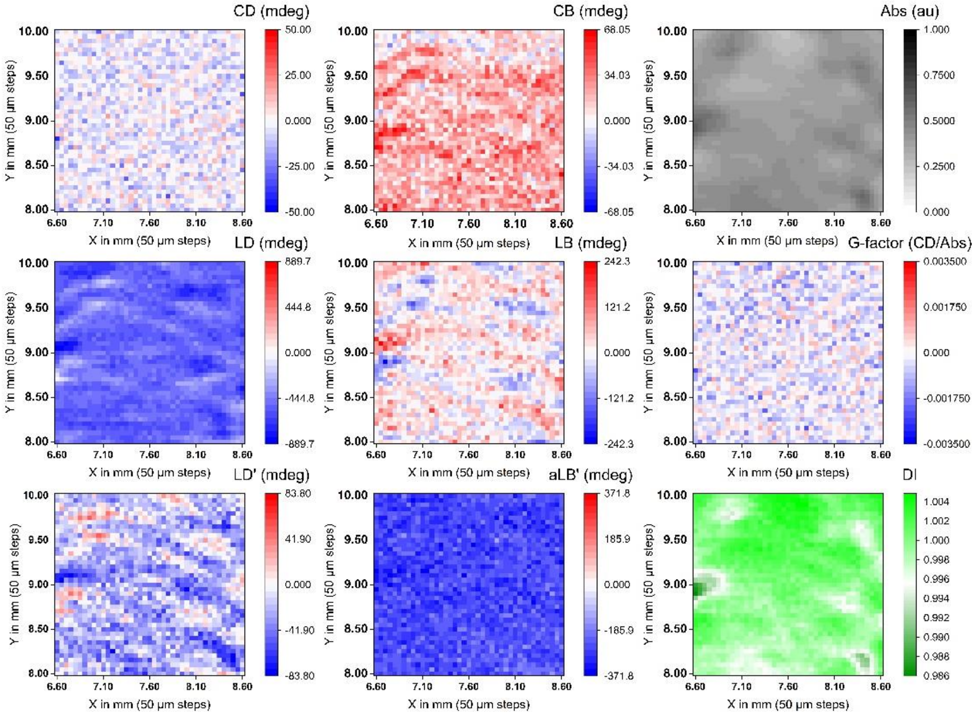

Supplementary Figure 3-22: MMP map of racemic valinol macrocycle thin film at 41 x 41 steps of 50 µM recorded at 546 nm, baseline corrected for the glass substrate and processed using the Analytic Inversion method.

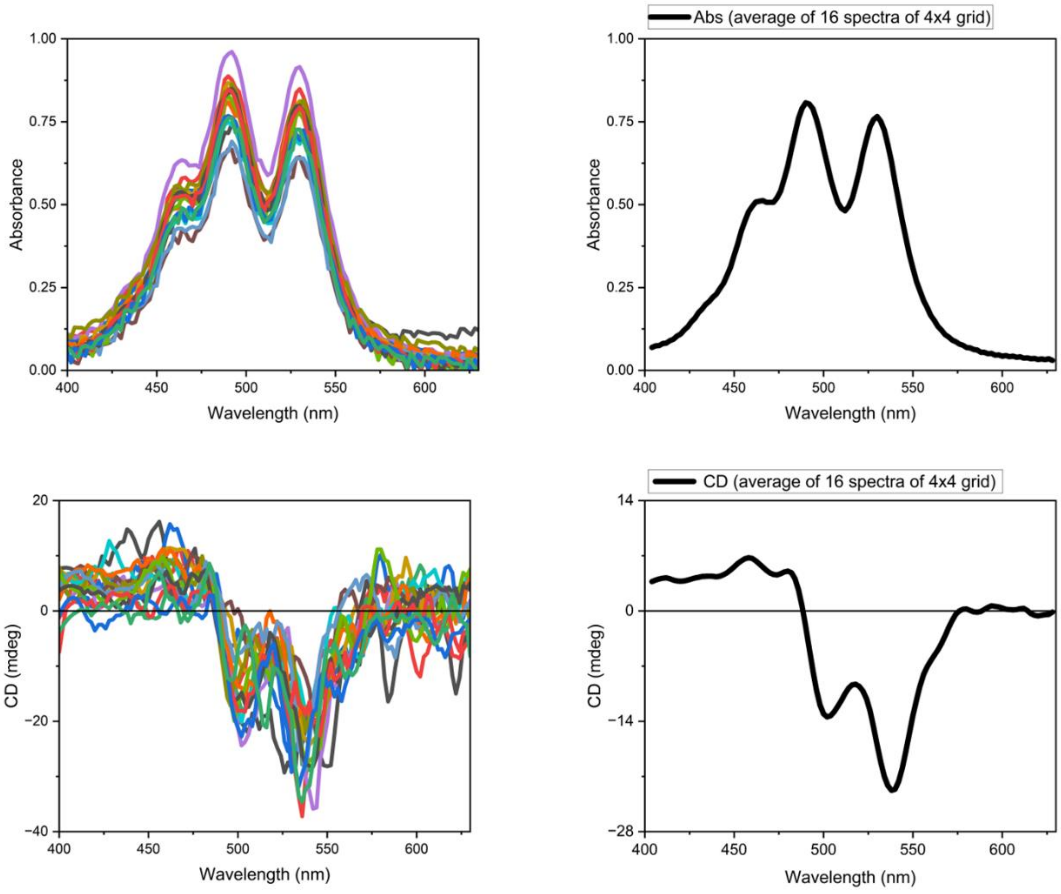

Supplementary Figure 3-23: CD and absorbance spectra of D-valinol macrocycle thin film recorded at 16 different positions on the film over an area of 2 mm^2 (666 um steps) measured by MMP and processed with the Analytic Inversion method. The g_{abs} at 502 nm was calculated to be $-1x10^{-3}$.

Macrocycle–coronene host–guest single crystal measurements in fomblin

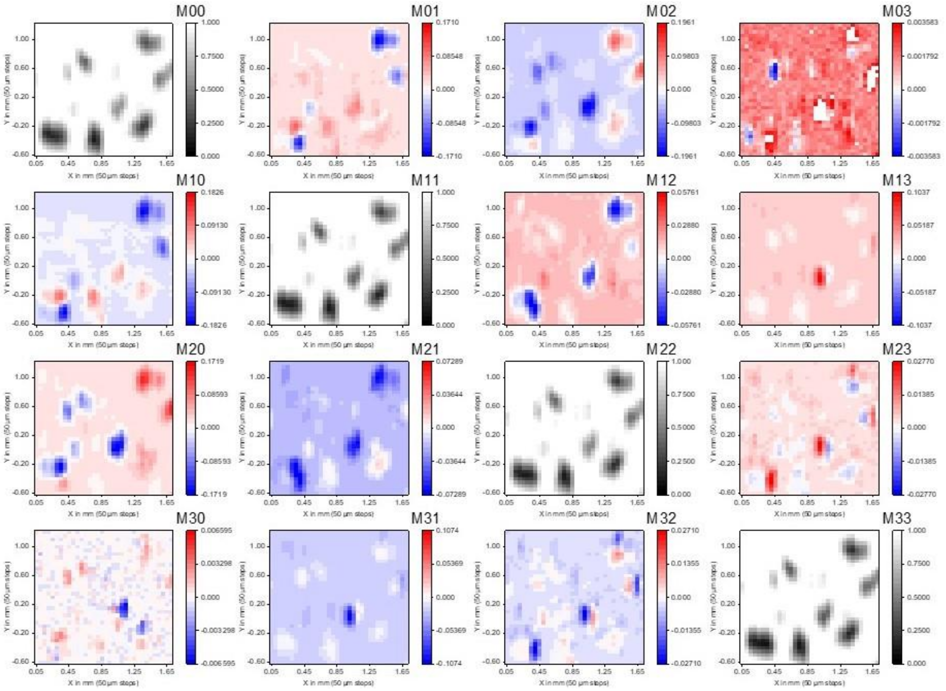

Supplementary Figure 3-24: 4x4 Mueller-Matrix of macrocycle-coronene host-guest crystals, recorded at 404 nm by MMP.

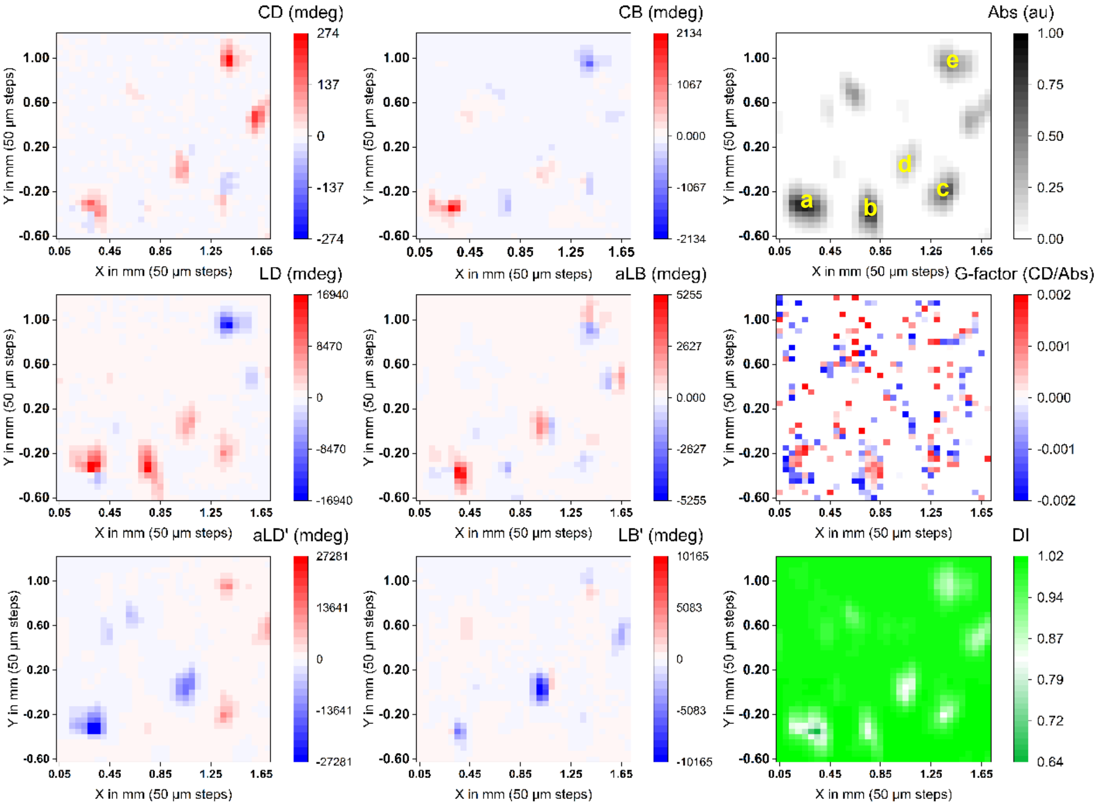

Supplementary Figure 3-25: MMP map of macrocycle-coronene host-guest single crystals, mapped at 404 nm, baseline corrected against fomblin and processed using the Analytical Inversion method. Crystals are marked with letters **a-e** for identification in **Supp. Figs. 3-23** to **3-28**.

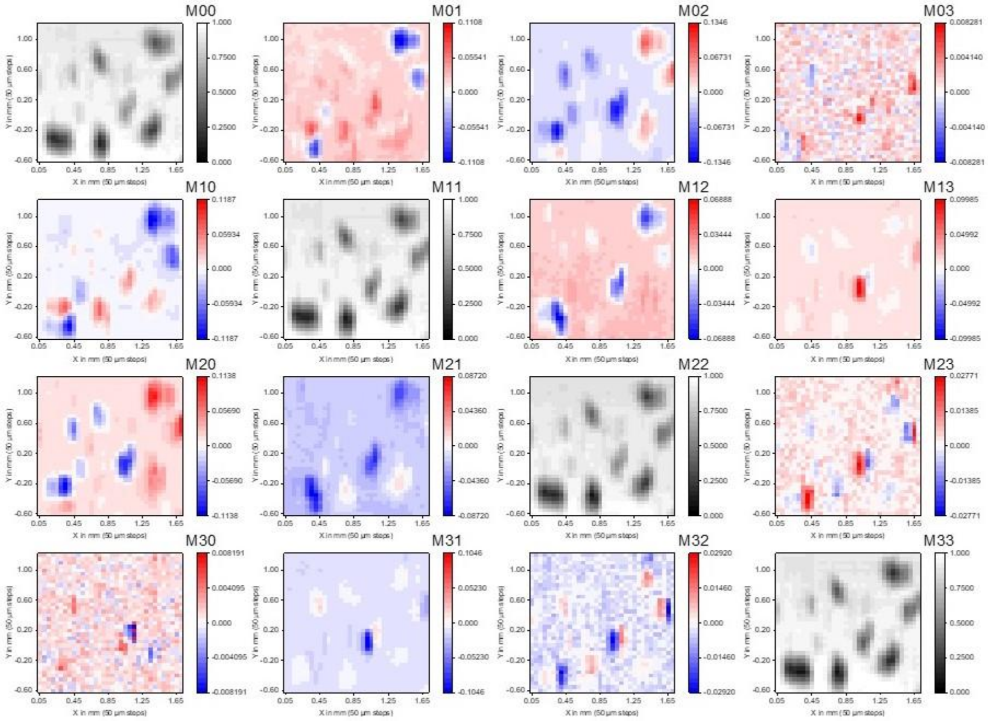

Supplementary Figure 3-26: 4x4 Mueller-Matrix of macrocycle-coronene host-guest crystals, recorded at 625 nm by MMP.

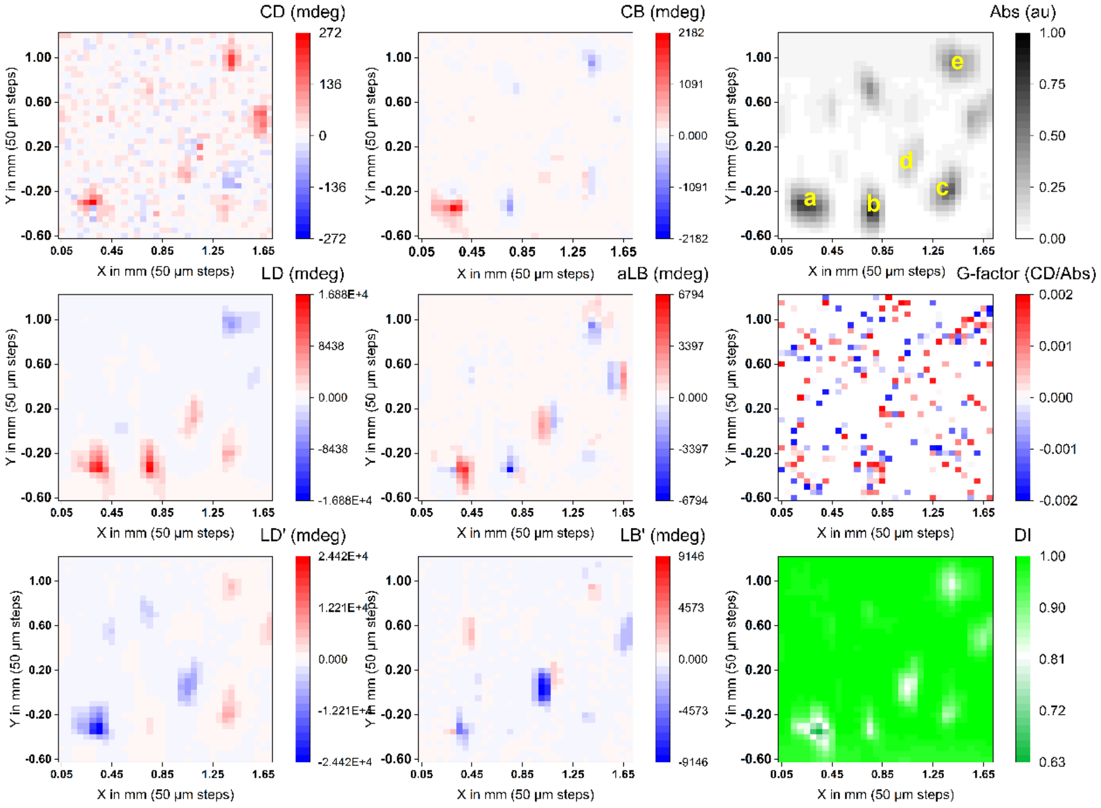

Supplementary Figure 3-27: MMP map of macrocycle-coronene host-guest single crystals, mapped at 625 nm, baseline corrected against fomblin and processed using the Analytical Inversion method. Crystals are marked with letters **a-e** for identification in **Supp. Figs. 3-23** to **3-28**.

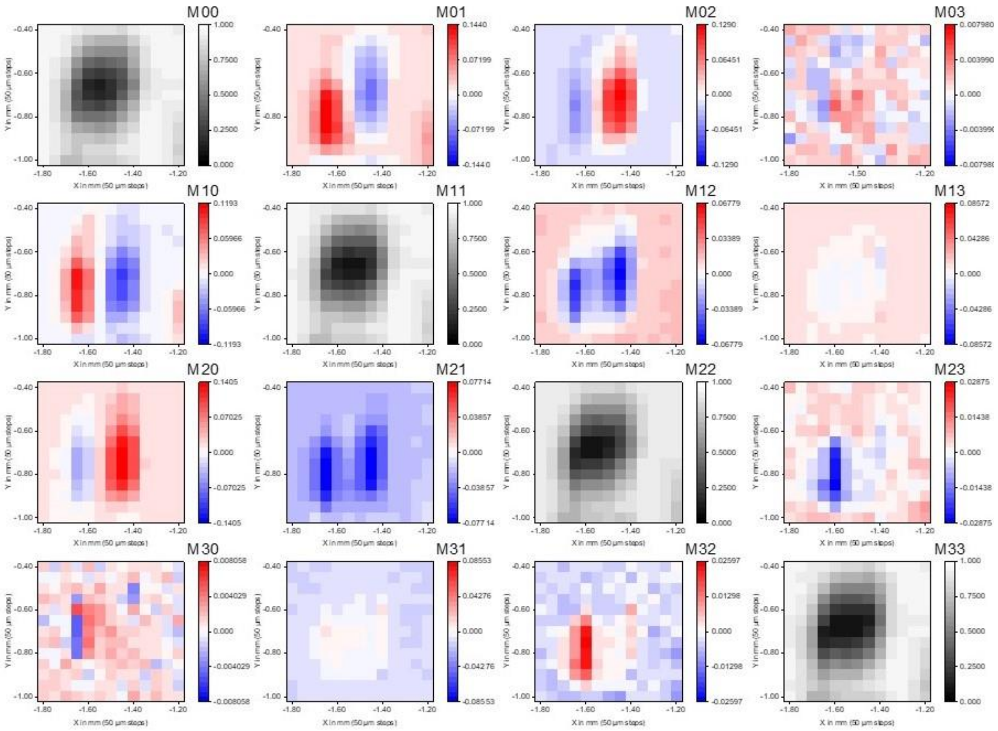

Supplementary Figure 3-28: 4x4 Mueller-Matrix of macrocycle-coronene host-guest crystal **a**, recorded at 625 nm by MMP, after rotating by 90 degrees.

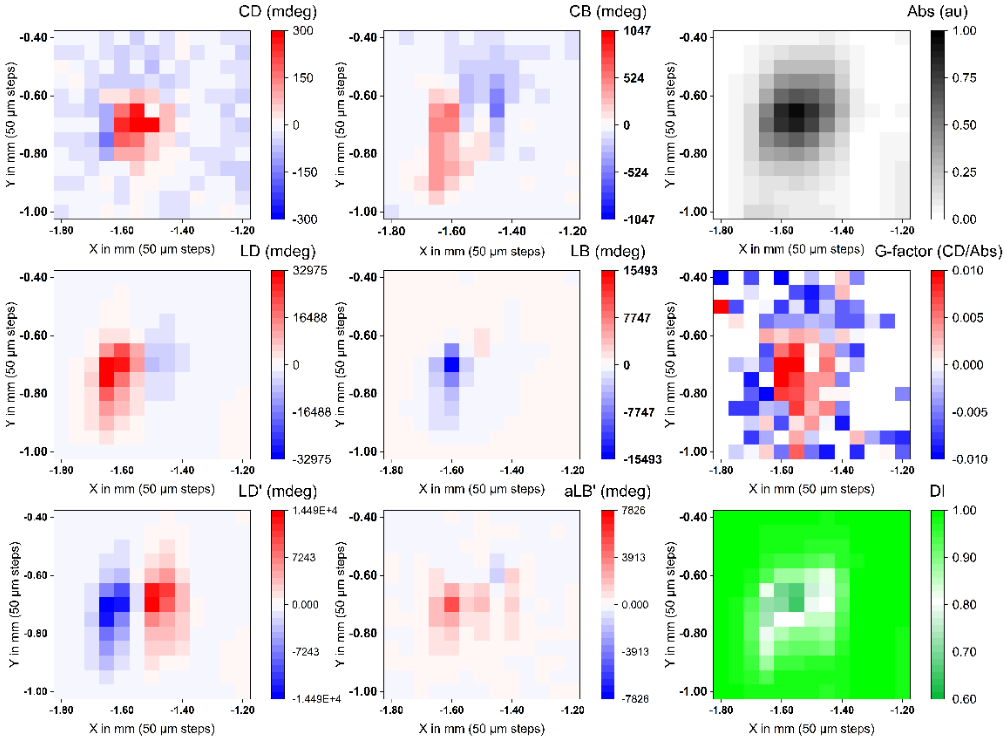

Supplementary Figure 3-29: MMP map of macrocycle-coronene host-guest crystal **a**, rotated by 90°and mapped at 625 nm, baseline corrected against fomblin and processed using the Analytical Inversion method.

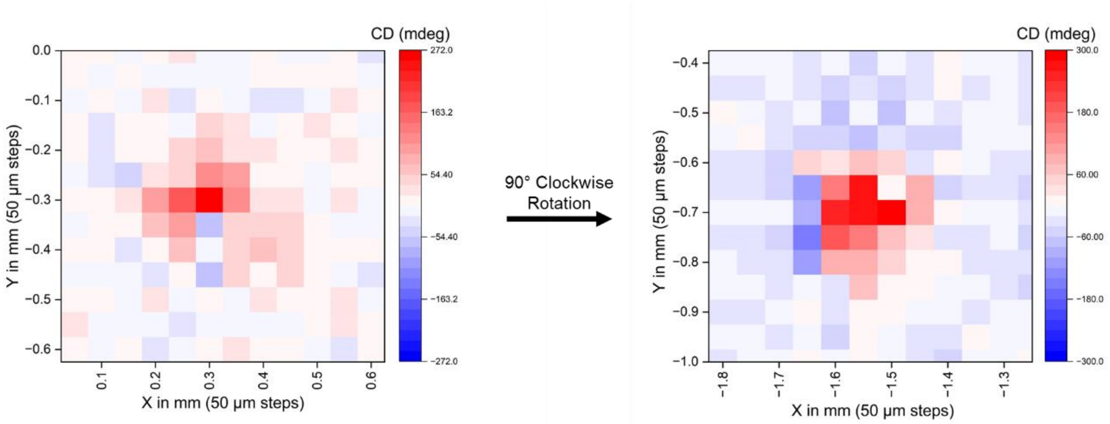

Supplementary Figure 3-30: Close-up of the MMP maps of macrocycle-coronene host-guest crystal **a**, before and after rotation by 90° mapped at 625 nm, baseline corrected against fomblin and processed using the Analytical Inversion method.

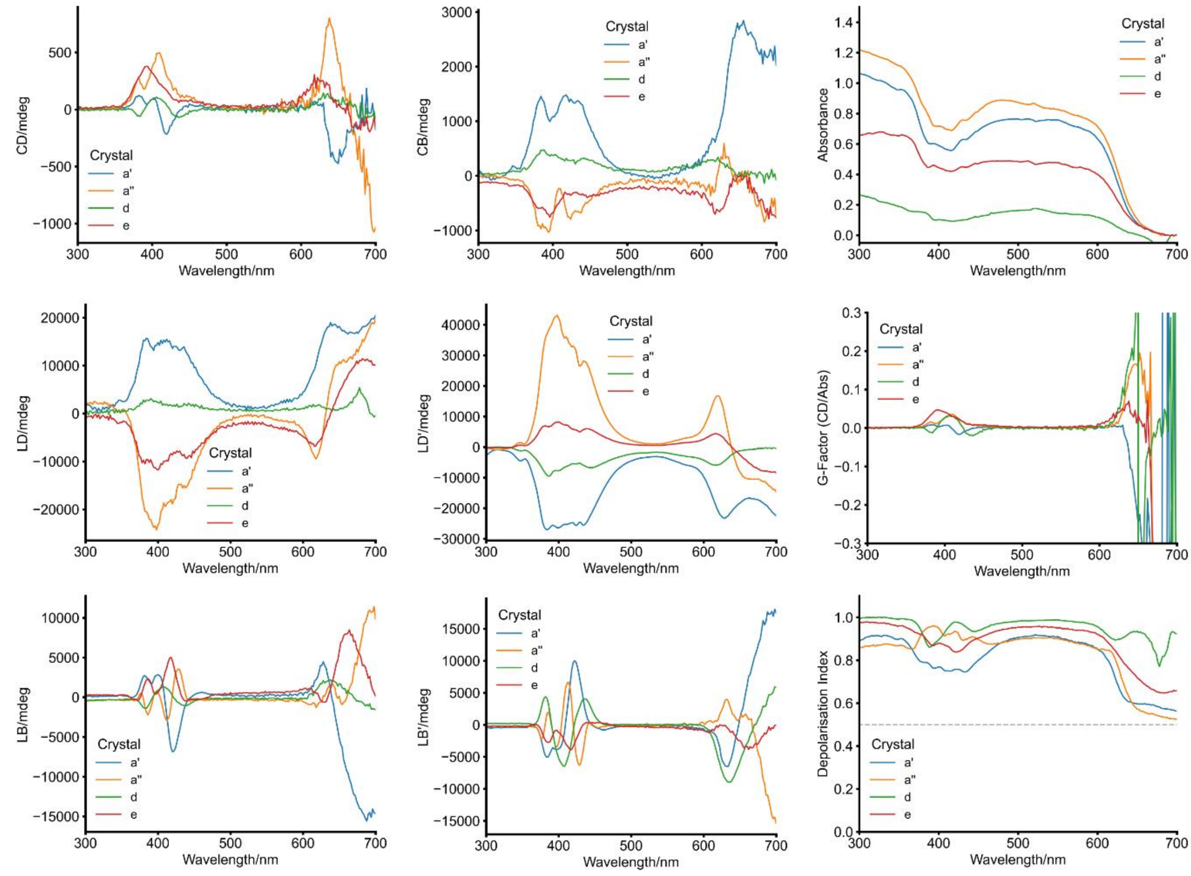

Supplementary Figure 3-31: CD, CB, Abs, LD, LB, g-factor, LD', LB' and DI spectra of crystals **a** (at two locations, **a'** and **a''**), **d and e**, processed using the Analytic Inversion method with the fomblin MMP spectrum subtracted as baseline. The g_{abs} at wavelengths > 675 nm are neglected due to noise.

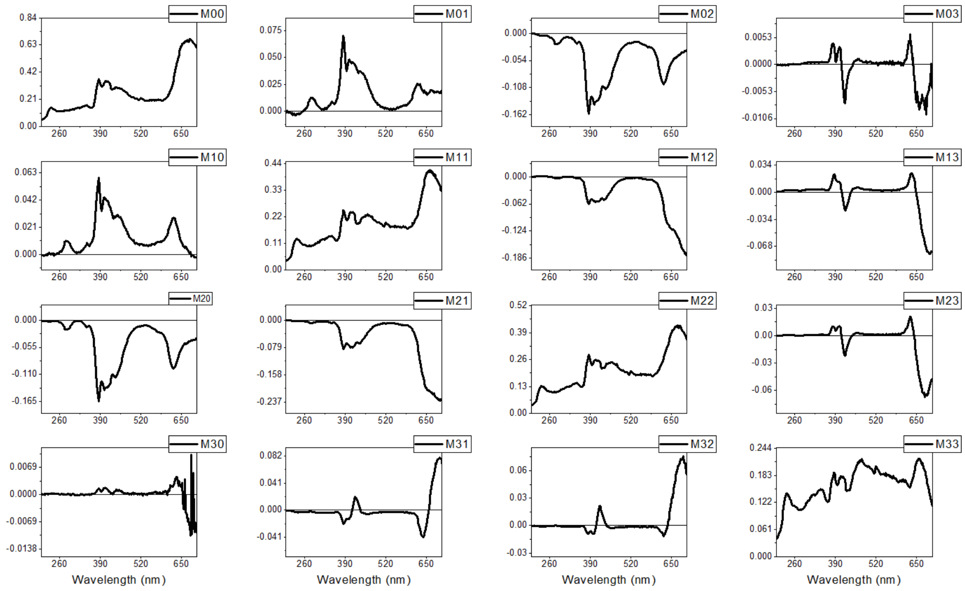

Supplementary Figure 3-32: 4x4 Mueller Matrix elements of crystal **a** at location 1 (**a'**) from **Supplementary Figure 3-22** as measured by MMP.

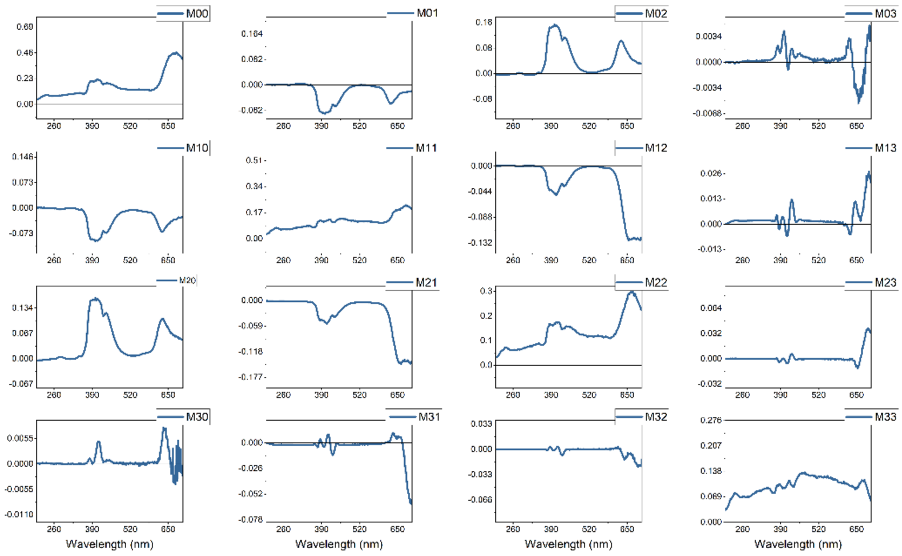

Supplementary Figure 3-33: 4x4 Mueller Matrix elements of crystal **a** at location 2 (**a''**) from **Supplementary Figure 3-22** as measured by MMP.

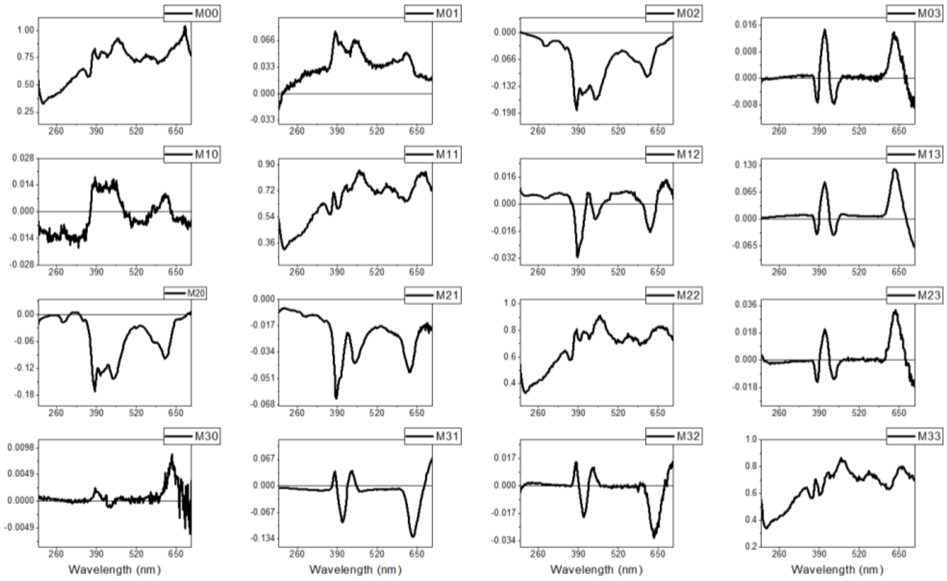

Supplementary Figure 3-34: 4x4 Mueller Matrix elements of crystal **d** from **Supplementary Figure 3-22** as measured by MMP.

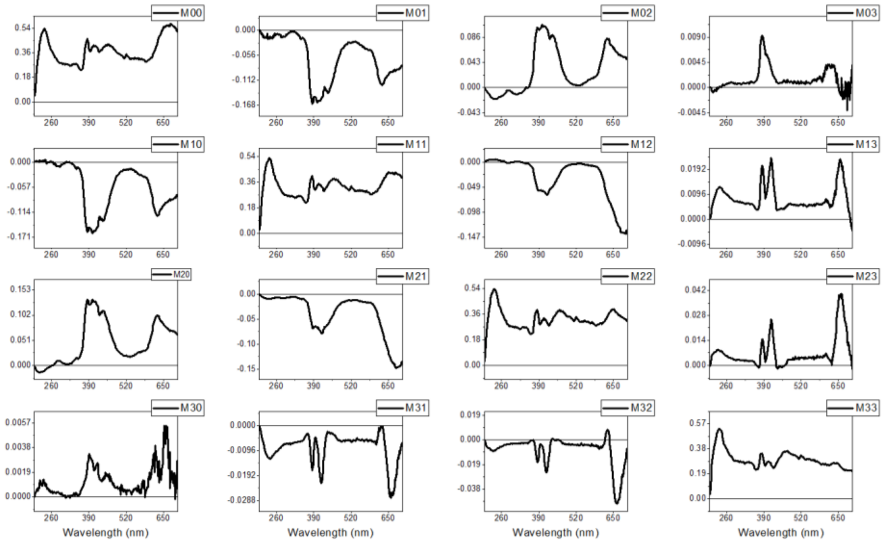

Supplementary Figure 3-35: 4x4 Mueller Matrix elements of crystal **e** from **Supplementary Figure 3-22** as measured by MMP.

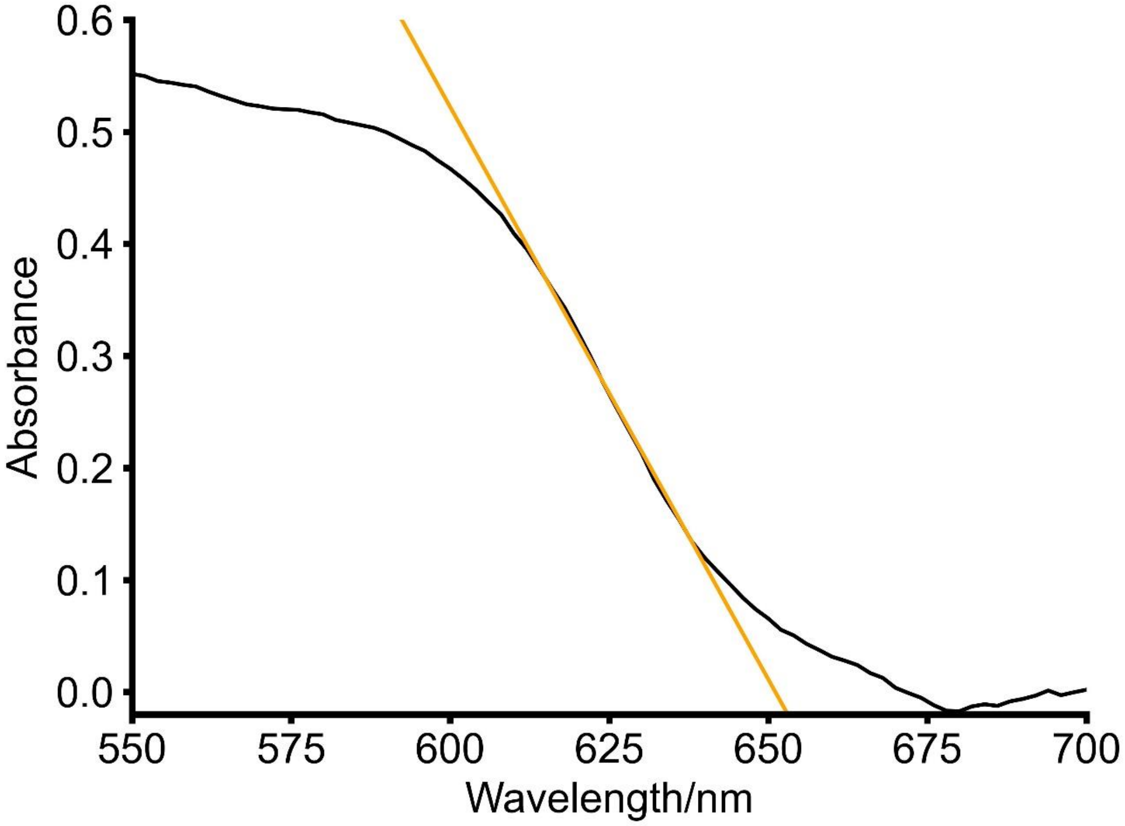

Supplementary Figure 3-36: Determination of the bandgap from an average absorbance spectrum of macrocycle-coronene host-guest co-crystals recorded by MMP. A trendline was fitted for the datapoints from 612 nm to 640 nm, resulting in an x-intercept at ~650 nm, which corresponds to a photon energy of 1.91 eV.

4) CPL measurements

Spectroscopy:

CPL measurements were taken on a (modular) CPL spectrometer.[28] Emission spectra were recorded with 2 nm step size and the slits of the detection monochromator were set to a slit width corresponding to a spectral resolution of 7 nm. Instrument errors are inherently minimised below the detection level due to a good signal to noise ratio of the photon detector, resulting in errors in g_{lum} of ± 5 × 10^{-5}. NB: The lower energy detection limit of the CPL detector is 800 nm.

Microcrystals were mounted in a 45°/45° setup to avoid light-guiding and reflections from the sample (**Supplementary Figure 4-1**). The microcrystal samples were excited at 570 nm at maximum detector gain.

Microscopy:

Circular Polarisation Luminescence Laser Scanning Confocal Microscope (CPL-LSCM) was performed on an adapted commercial LSCM (SP5 II, Leica Microsystems) as described.[4] Macrocycle only samples were excited at 458 nm and emission recorded from 500-700 nm, with a 570 nm long pass (LP) filter used for EDCC imaging, whereas co-crystals were excited at 543 nm with a LP 715 nm filter for EDCC.

For the macrocycle crystals, the total internal reflection was minimised by imaging crystals with perpendicular surfaces to the optical axis of imaging (i.e., optical axis is always aligned with the c axis of the crystal, as for MMP) and ii) elimination of scattered reflected light by imaging crystals that are larger the applied LSCM pinhole determined axial resolution (z direction), which at 458 nm excitation is 785 nm, i.e. much less than the 100 µm-sized macrocycle crystals being imaged.

Due to their thin nature and to avoid multiple reflections, the co-crystal CPL-LSCM measurements were taken with the microscope focussed on the glass support, thereby recording the reflected light, inverting the sign of the CPL recorded for the crystals.

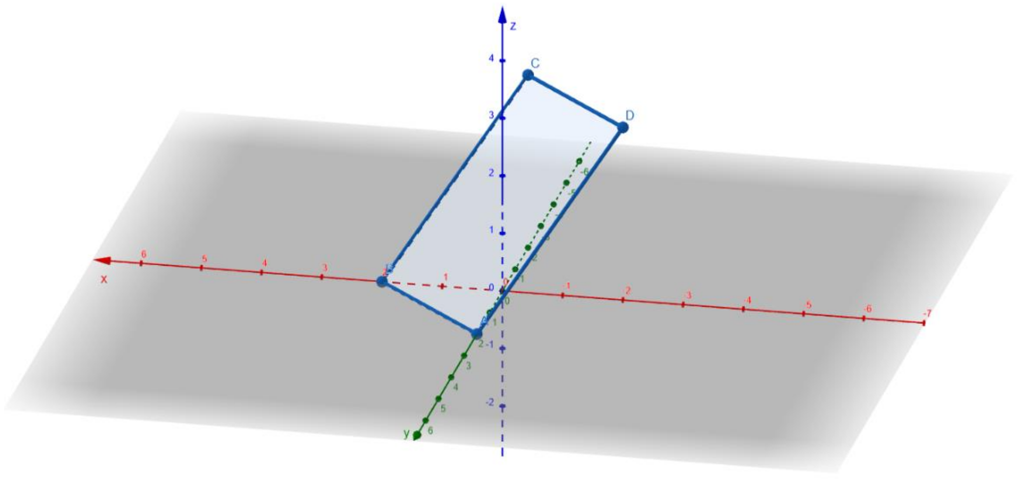

Supplementary Figure 4-1: Schematic of sample setup for CPL measurement of macrocycle-coronene microcrystals (from drop casting onto a slide). The sample (spanned by the points ABCD) is irradiated from the y-axis and detected along the x-axis (i.e. sample at 45° to light source and detector), with a sample tilt to the z-axis of 45°. This figure was created in GeoGebra.

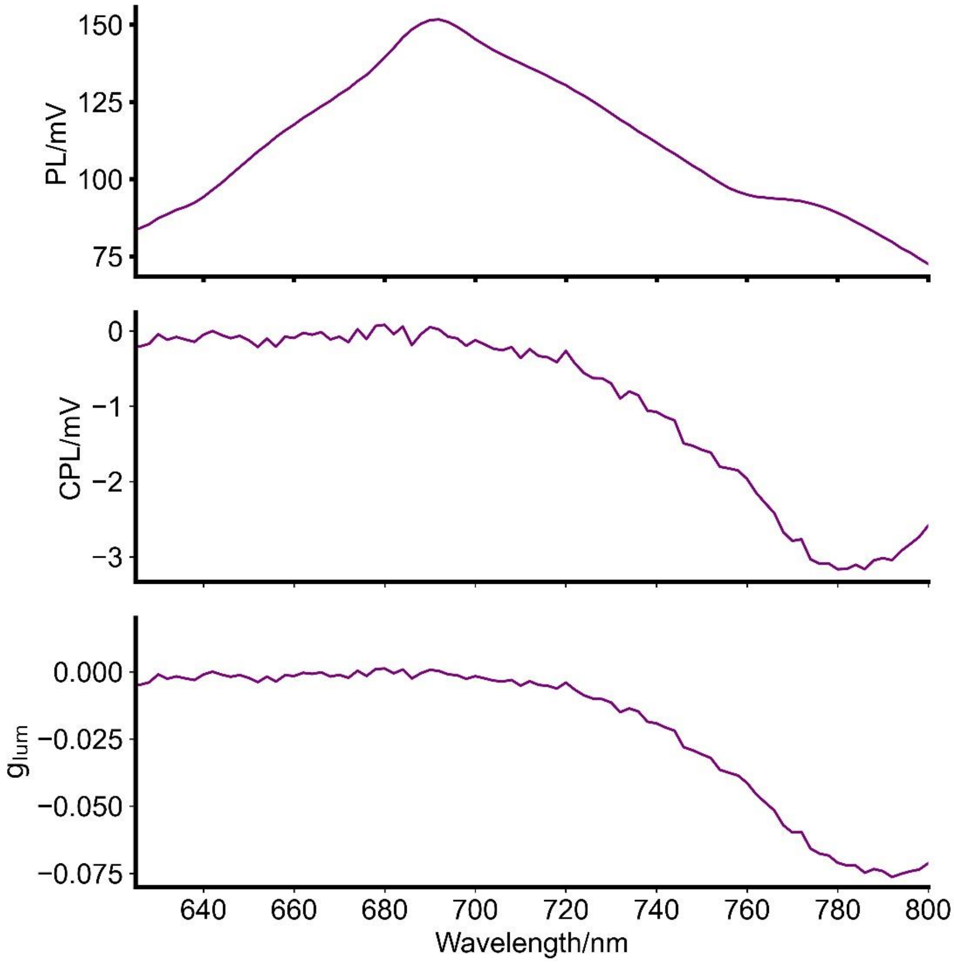

Supplementary Figure 4-2: PL and CPL spectra of the macrocycle-coronene microcrystals (**Supp. Fig. 5-8**) and corresponding calculated g_{lum}.

Single crystal CPL-LSCM measurements focussed on crystal edge

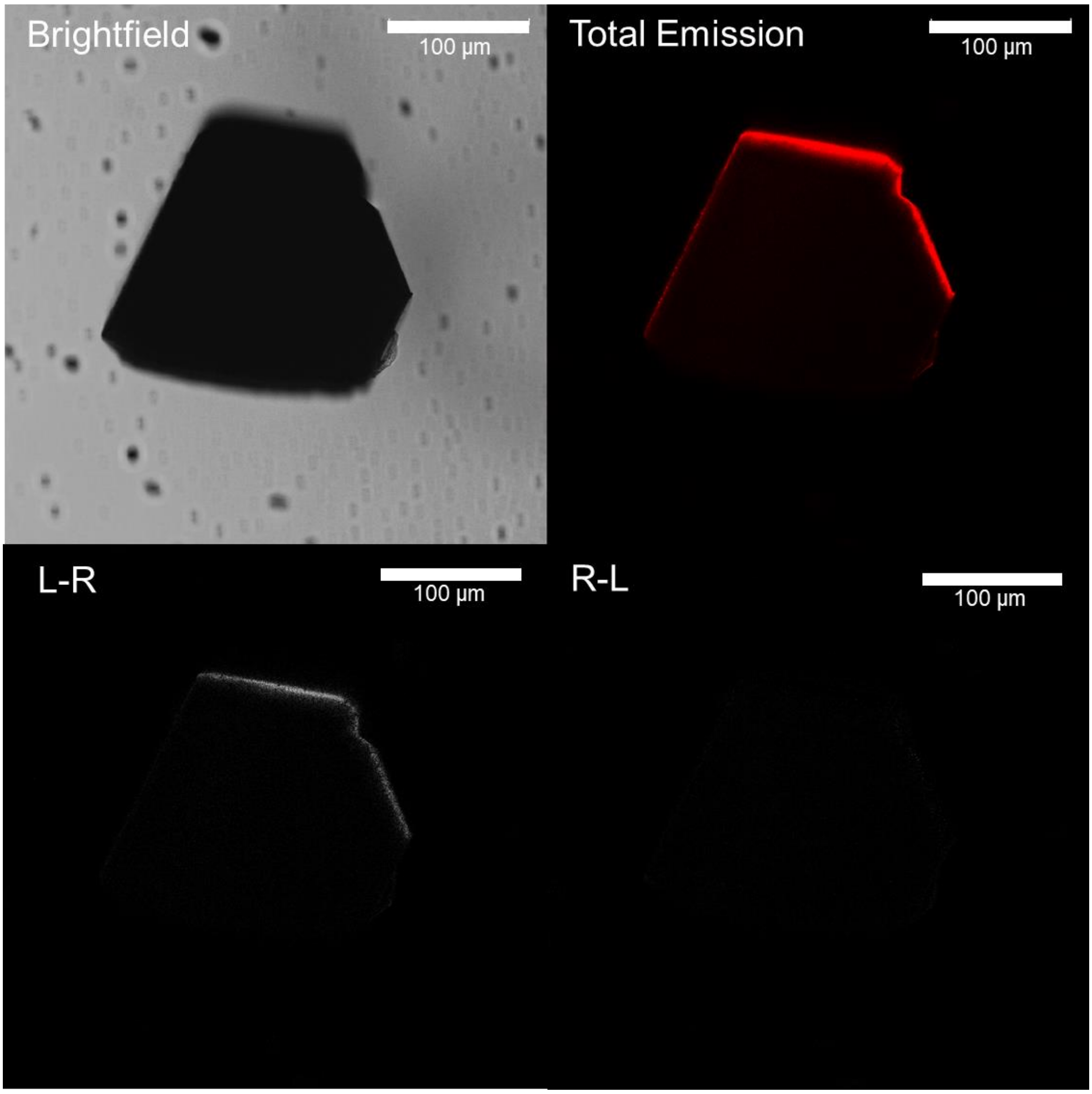

Supplementary Figure 4-3: CPL-LSCM measurements of a macrocycle single crystal (λ_{ex} = 458 nm, λ_{em} > 570 nm) focussed on the crystal edge, with left- (L−R) and right-handed (R−L) CPL images showing the enantioselective differential chiral contrast (bottom).

Differential images of macrocycle single crystals at face

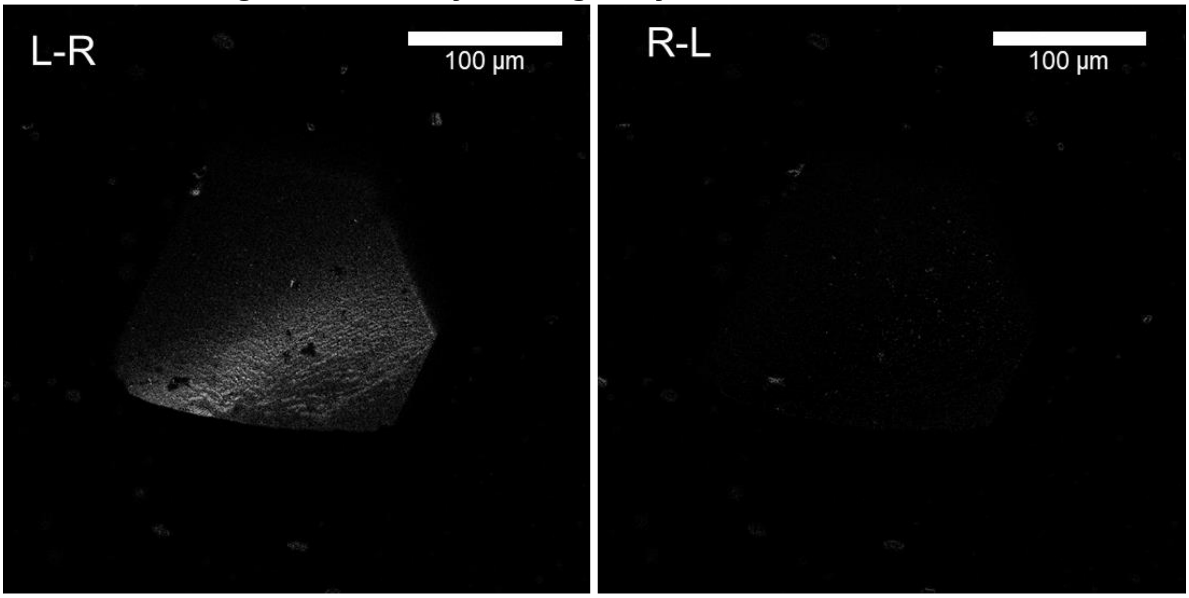

Supplementary Figure 4-4: Differential images of macrocycle single crystal CPL measured by CPL-LSCM on the crystal face (λ_{ex} = 458 nm, λ_{em} > 570 nm), with left- (L−R) and right-handed (R−L) CPL images showing the enantioselective differential chiral contrast.

Measurement of the face of a macrocycle single crystal

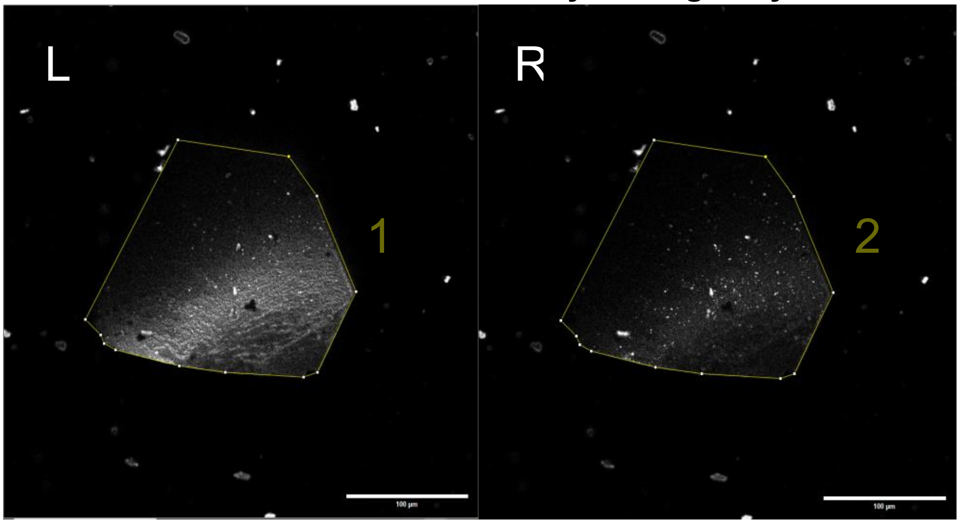

Supplementary Figure 4-5: Quantification of single crystal CPL microscope Images on the face.

**Area**	**Average 8-Bit Pixel Value**
Left Channel (1)	46.697
Right Channel (2)	19.474
Ratio	2.67:1

Measurement of macrocycle single crystal edge

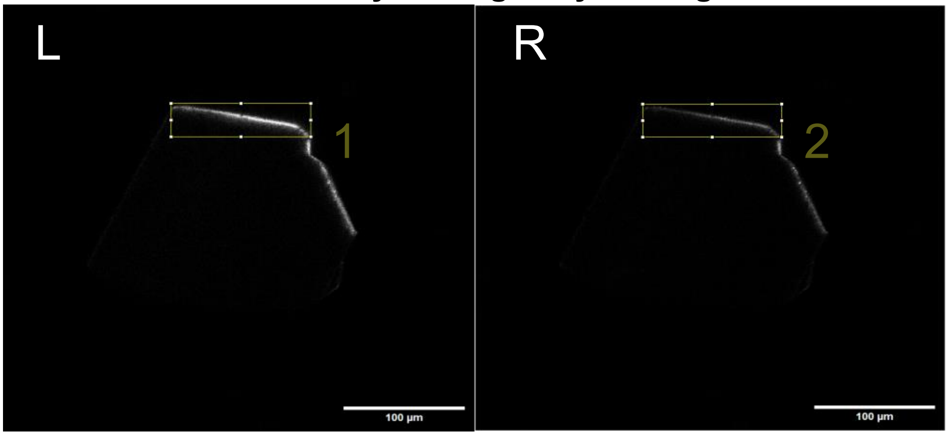

Supplementary Figure 4-6: Quantification of crystal edge. Reduced internal reflection at the crystal edge leads to a larger CPL bias.

Area	Average 8-Bit Pixel Value
Left Channel (1)	30.078
Right Channel (2)	10.070
Ratio	2.99:1

Measurement of crystal face of cracked crystals

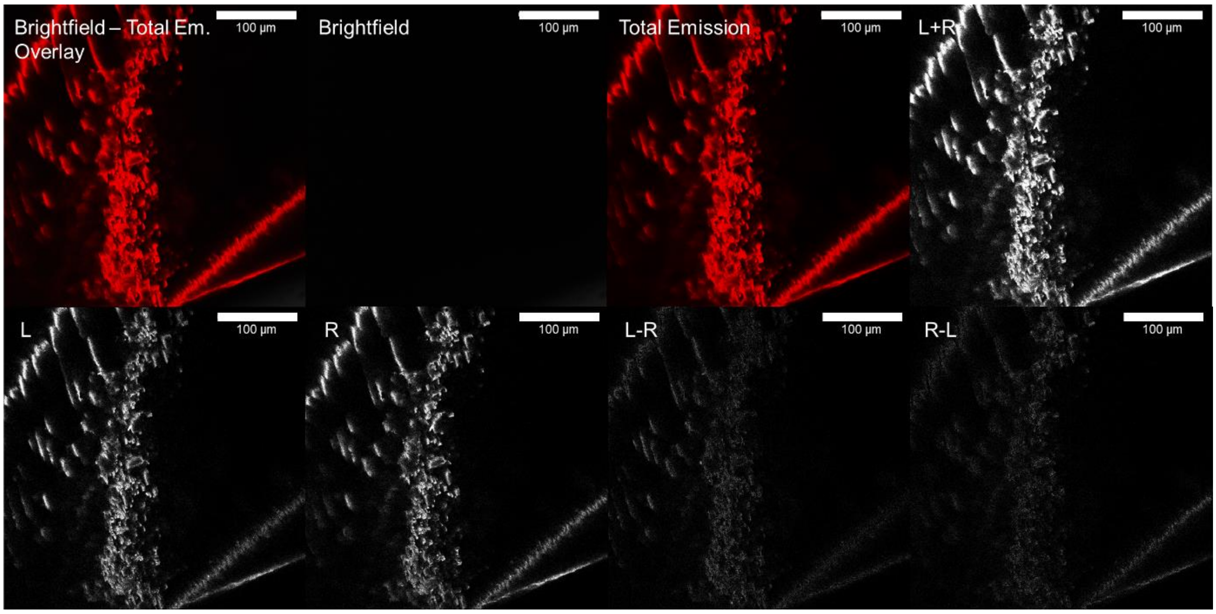

Supplementary Figure 4-7: CPL Microscopy image of cracked crystals as a result of solvent loss from the crystal lattice (λ_{ex} = 458 nm, λ_{em} > 570 nm) with left- (L−R) and right-handed (R−L) CPL images showing the enantioselective differential chiral contrast. The cracking drastically increases the total number of internal reflections, racemising the emitted light, resulting in loss of CPL.

**Area**	**Average 8-Bit Pixel Value (Full Image)**
Left Channel	16.398
Right Channel	15.934

Determination of the enantioselective differential chiral contrast dissymmetry factor (g_{EDCC}) for macrocycle single crystals

We calculated an EDCC dissymmetry factor (g_{EDCC}) for crystals following published methods.[4] In the absence of a racemic crystal sample, we calculated the Bias factor (B) using the cracked crystals, which show a loss of CPL under microscopy (**Supplementary Figure 4-8**).

From the EDCC images of the cracked crystals we calculated a (left-handed) contrast transfer function (CTF):

CTF = 0.232

From this, the bias factor B is half of the CTF as the bias is present equally in the Left and Right channels,

B = CTF / 2 = 0.116.

g_{EDCC} is then calculated using the total image 8-bit pixel values of the crystals.[4] This yields a g_{EDCC} of 0.079.

CPL-LSCM images of macrocycle-coronene single crystal and determination of g_{EDCC}

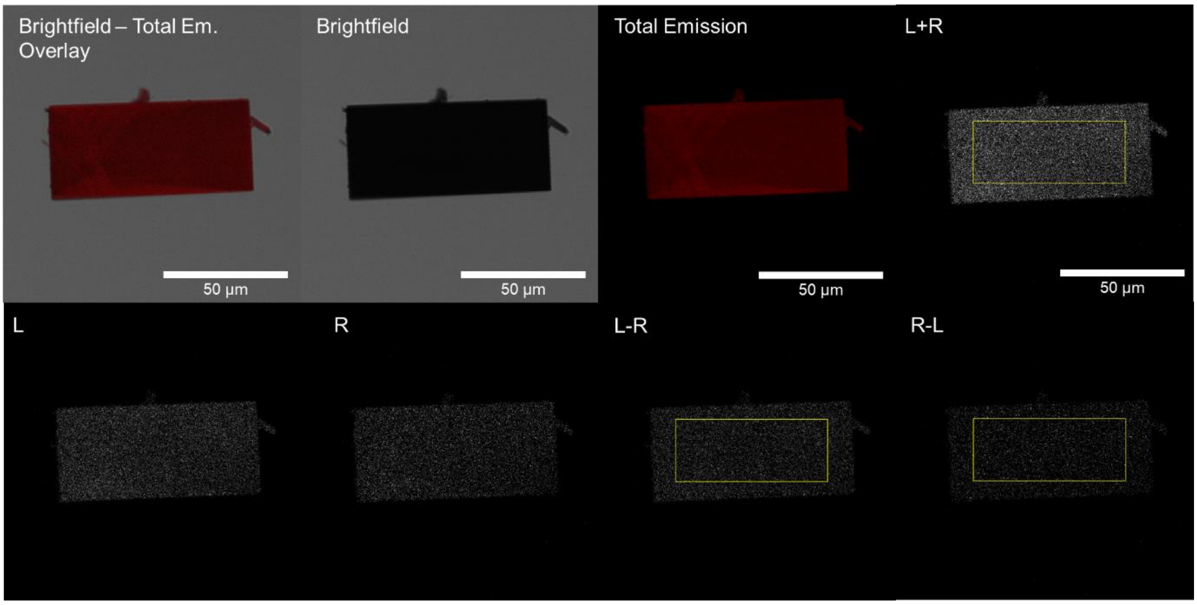

Supplementary Figure 4-8: CPL-LSCM images of a macrocycle-coronene host-guest single crystal (λ_{ex} = 543 nm, λ_{em} > 715 nm) with left- (L−R) and right-handed (R−L) CPL images showing the enantioselective differential chiral contrast. Yellow rectangle indicates the area of measurement for g_{EDCC}.

Area	Average 8-Bit Pixel Value
L+R	51.391
L-R	20.668
R-L	12.611

From this, a g_{EDCC} = 0.078 was calculated.

CPL-LSCM images of macrocycle-coronene microcrystals and determination of g_{EDCC}

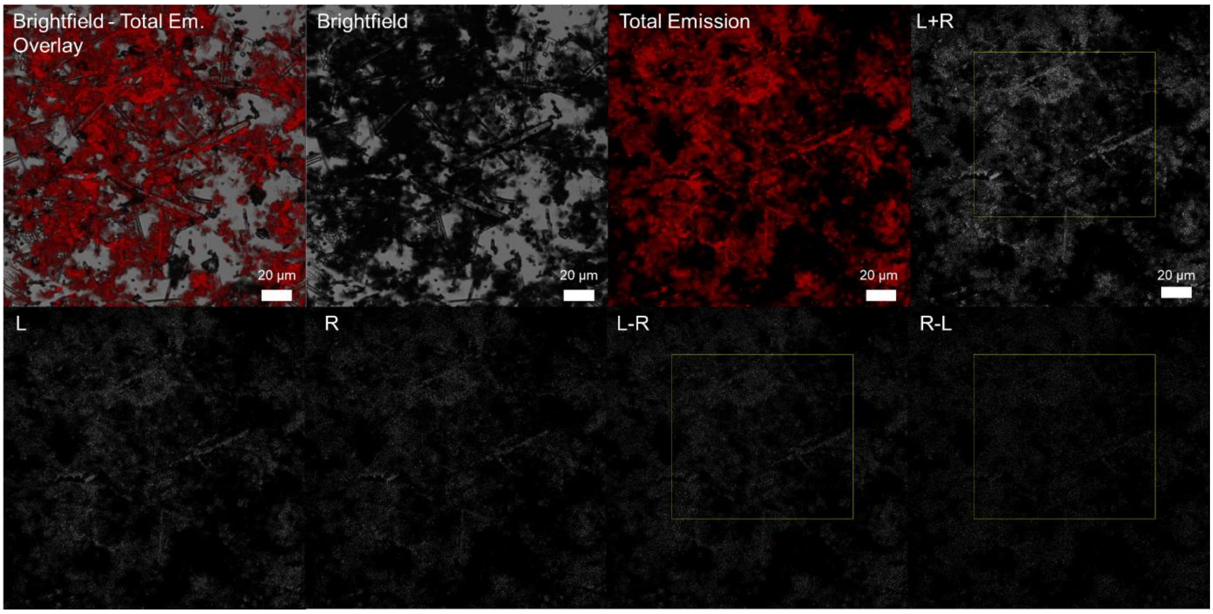

Supplementary Figure 4-9: Microscope and differential images of microcrystalline macrocycle-coronene host-guest complex using a slight excess of coronene (λ_{ex} = 543 nm, λ_{em} > 715 nm) with left- (L−R) and right-handed (R−L) CPL images showing the enantioselective differential chiral contrast. Yellow rectangles indicate area of measurement for g_{EDCC}. Note the non-emissive needles observed in the images correspond to excess coronene guest which crystallised.

Area	Average 8-Bit Pixel Value
L+R	21.351
L-R	9.419
R-L	6.238

From this, a g_{EDCC} = 0.078 was calculated.

5) Microscope images of macrocycle-coronene co-crystals

General
Brightfield and polarised microscope images were taken on a Zeiss Discovery.V8 Microscope equipped with an Axiocam 105 Color Camera at up to 8x zoom. Confocal microscope images were taken on a Leica SP5 II LSCM.

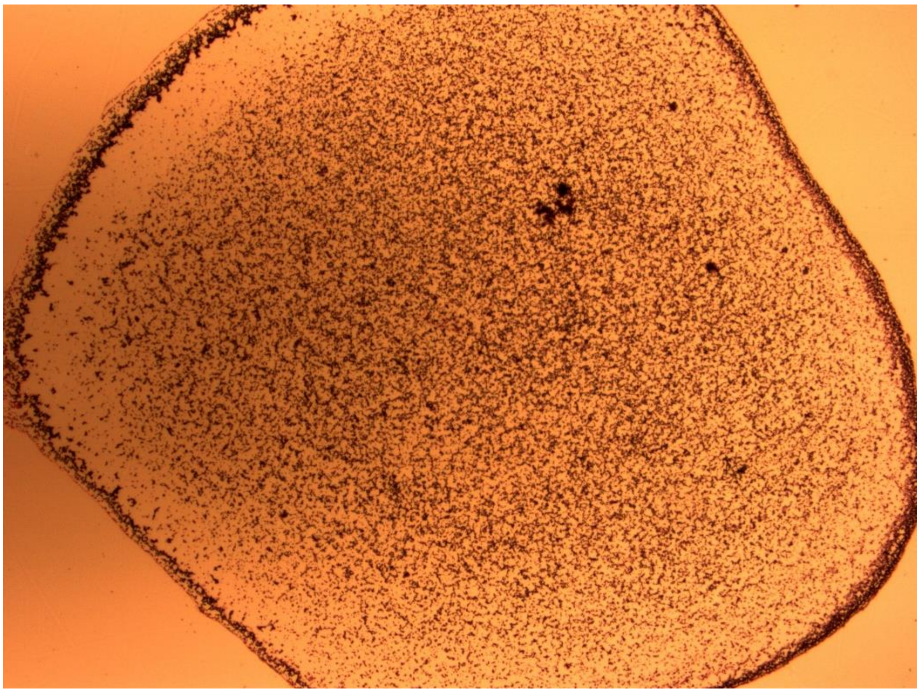

Supplementary Figure 5-1: Brightfield image of the microcrystals at lowest zoom.

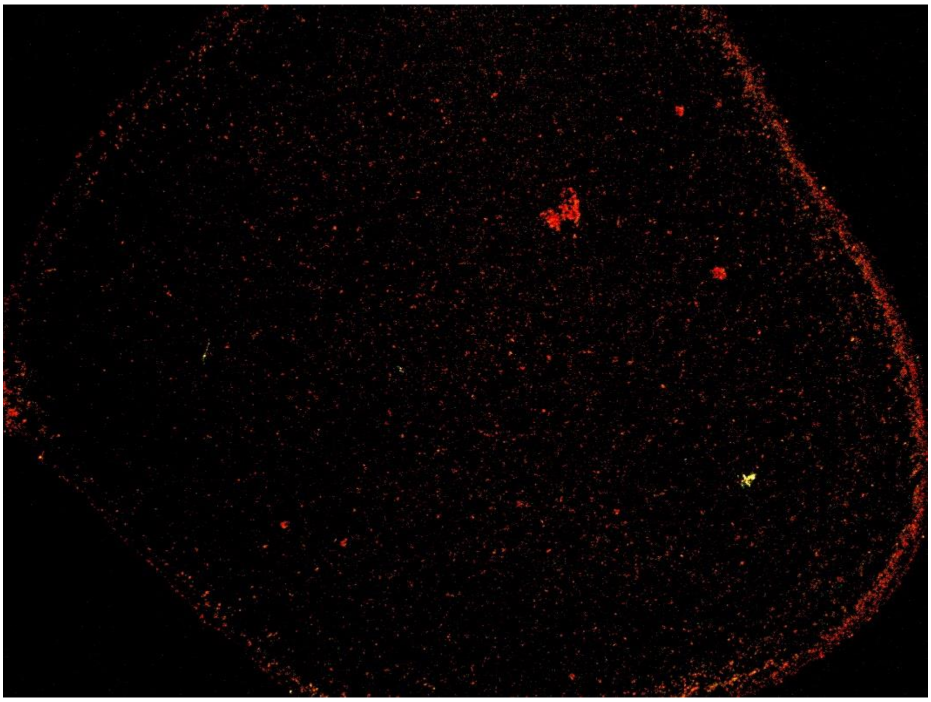

Supplementary Figure 5-2: Image of microcrystals from **Supplementary Figure 5-1** under cross-polarisation.

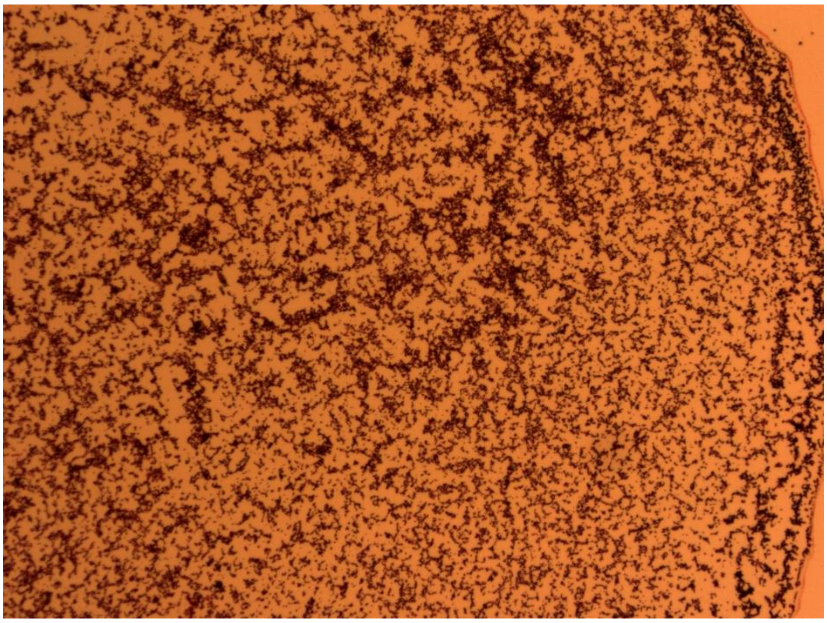

Supplementary Figure 5-3: Close-up microscopy image of the microcrystals under brightfield illumination.

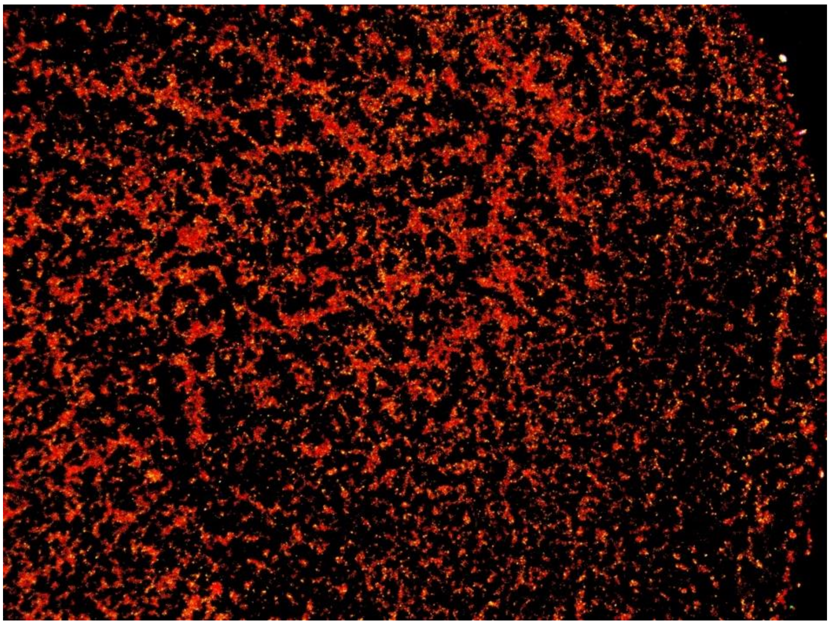

Supplementary Figure 5-4: Close-up microscopy image of the microcrystals under cross-polarisation.

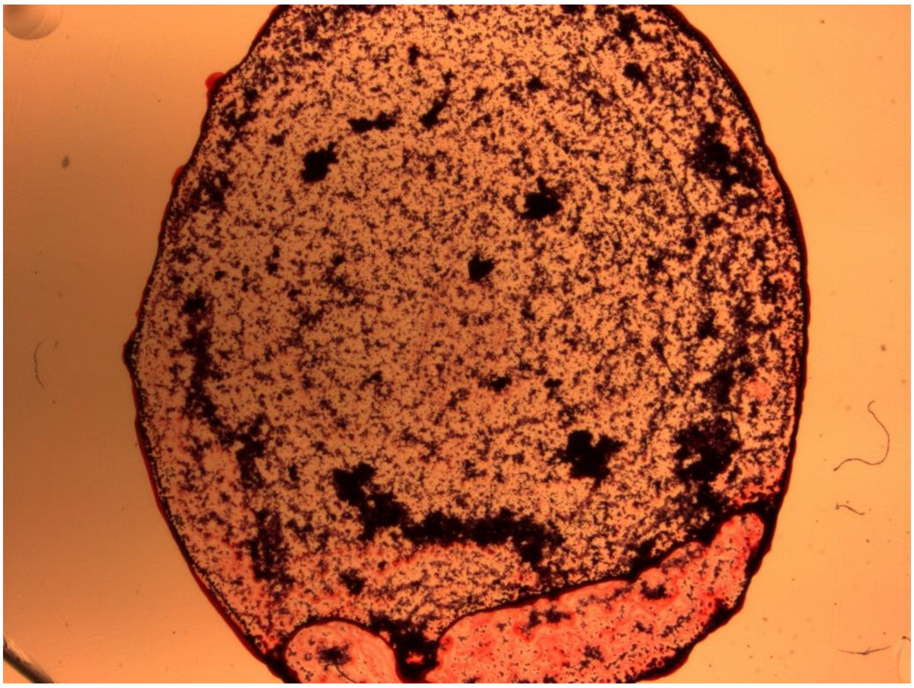

Supplementary Figure 5-5: Brightfield microscopy image of co-crystals and macrocycle film formed by drop casting a 1:1 macrocycle:coronene stoichiometric solution from toluene.

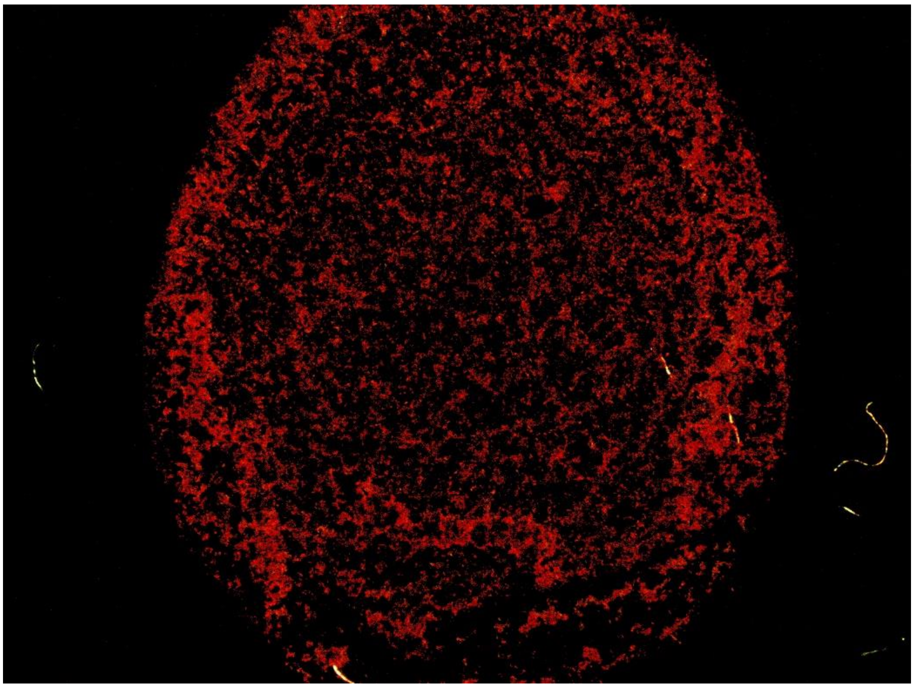

Supplementary Figure 5-6: Microscopy image of host-guest microcrystals from **Supplementary Figure 5-5** under cross-polarisation.

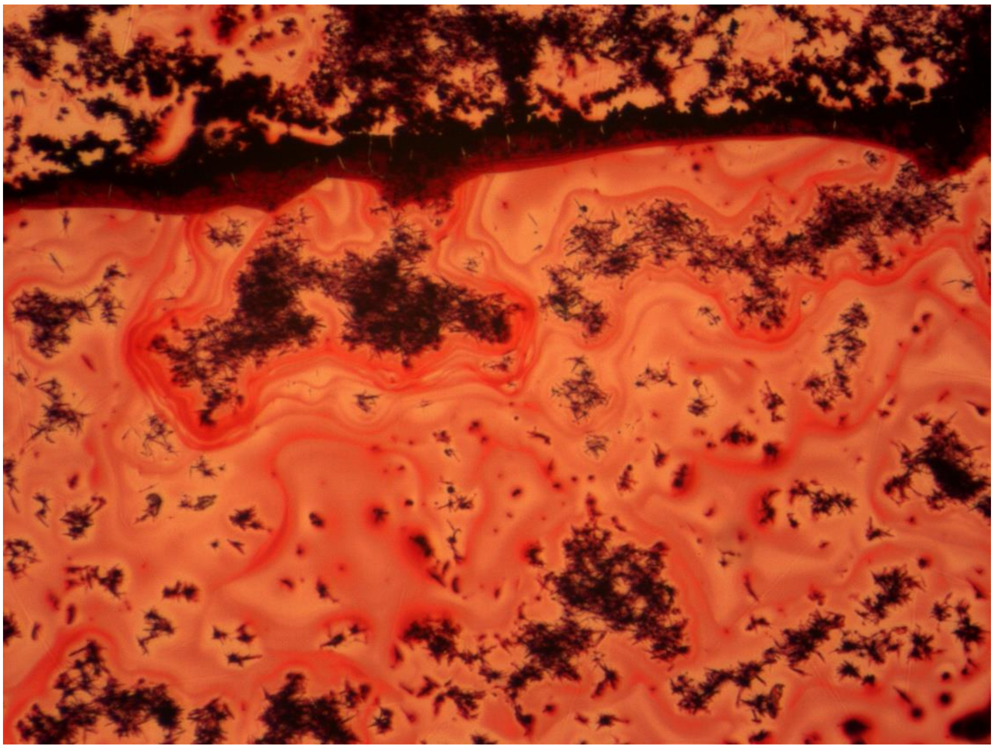

Supplementary Figure 5-7: Close-up of the boundary between host-guest microcrystals and macrocycle thin film regions of **Supplementary Figure 5-5**.

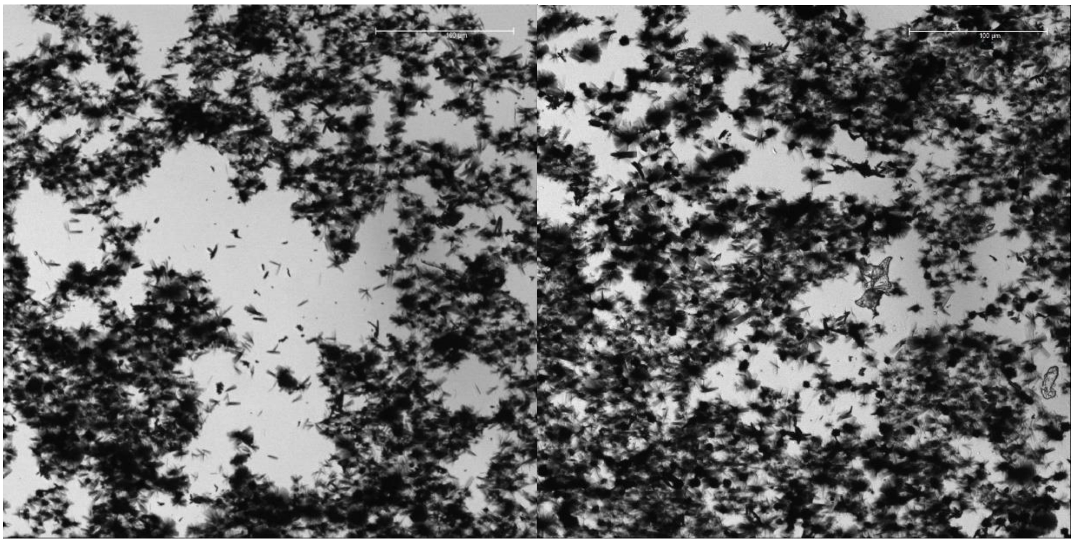

Supplementary Figure 5-8: Confocal microscopy images of macrocycle-coronene host-guest complex microcrystals. Scale bar = 100 µm.

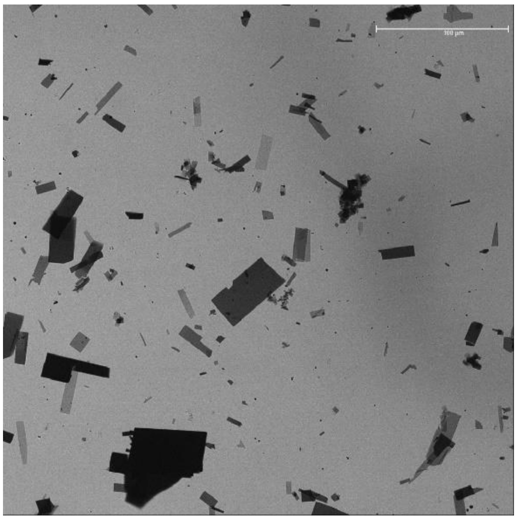

Supplementary Figure 5-9: Confocal microscopy image of macrocycle-coronene host-guest complex single crystals. Scale bar = 100 µm.

6) Powder X-ray diffraction

Powder X-ray diffraction experiments were performed on a Malvern PANalytical Empyrean X-ray diffractometer. Microcrystal samples of the complex were prepared by drop casting a concentrated solution of macrocycle (~5 mM) and excess coronene (~2.1 eq.) in toluene onto a zero-background silicon sample holder. The sample was then analysed using Copper Kα radiation from 2θ = 2° to 40°. The powder patterns for the host-guest complex crystals and coronene were predicted using 'Mercury' software from published single crystal data (CCDC: 1129883 for Coronene, CCDC: 2379186 for the complex). Indicative peaks of the complex found in the powder pattern are highlighted (*).

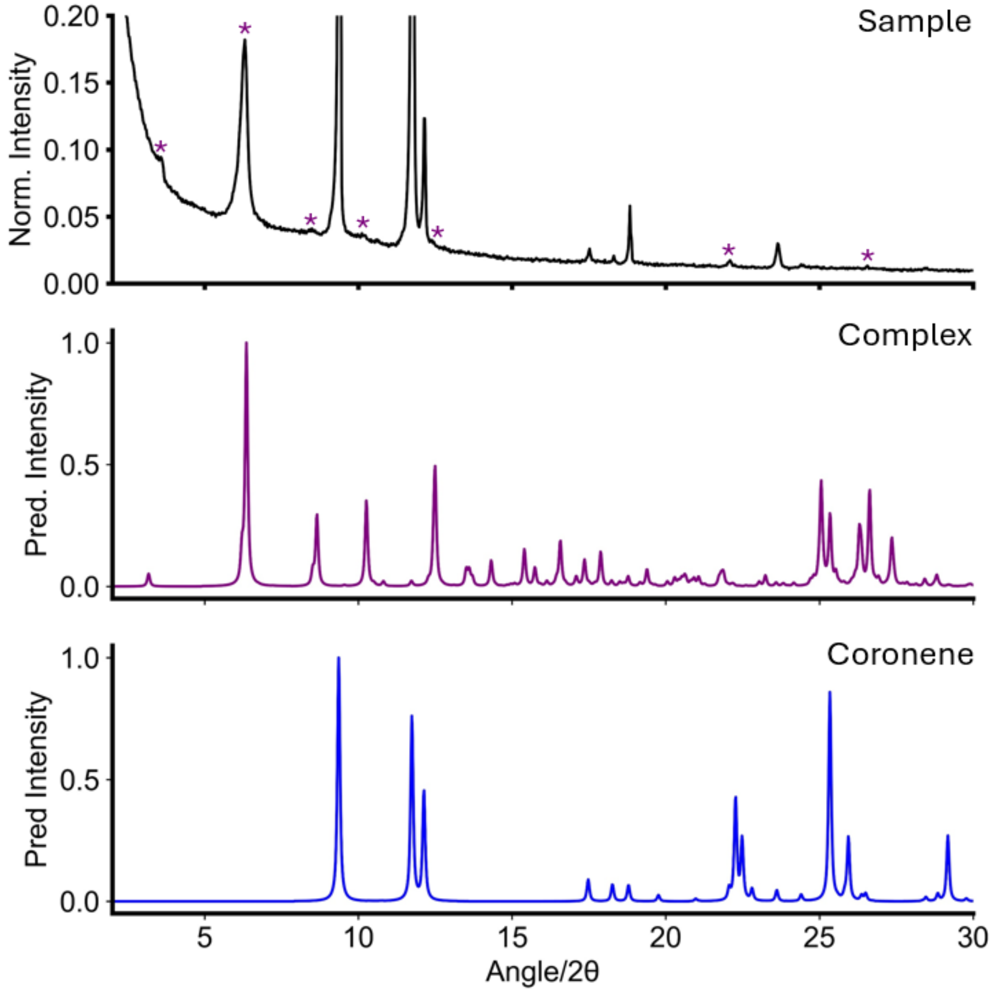

Supplementary Figure 6-1: Powder XRD pattern of the self-assembled material upon mixing of macrocycle with excess coronene. Besides excess coronene peaks, several characteristic peaks of the complex are observed (*). The peak at ~6° is particularly indicative, and contains the hkl = (1,0,0) and (0,0,2) reflections.

7) Atomic force microscopy

Atomic force microscopy measurements were performed on an AIST-NT SmartSPM 1000 scanning probe microscope. Thin film sample thickness was determined by measuring the depth of a thin scalpel scratch through the film to the glass substrate.

Film **1** thickness: 120 nm

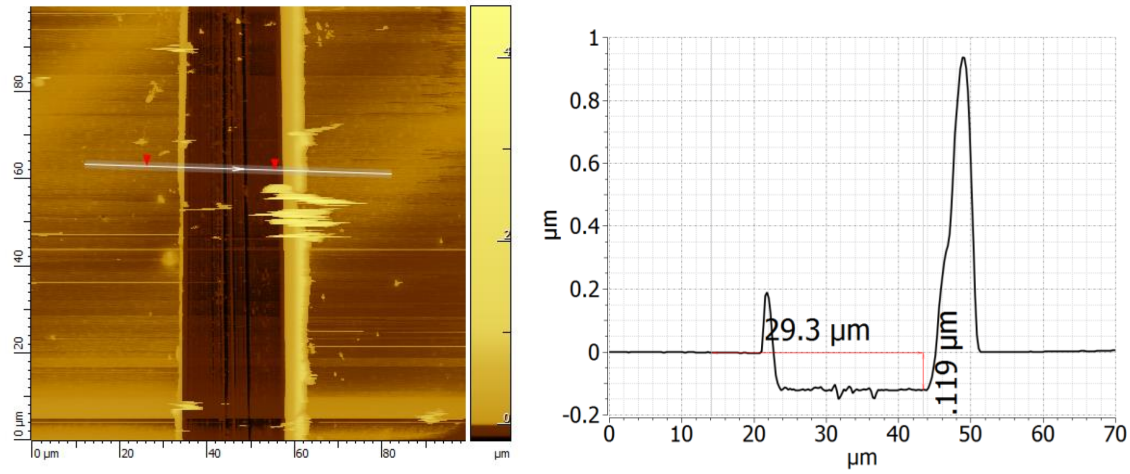

Supplementary Figure 7-1: AFM image and depth measurement of macrocycle thin film **1**.

8) Computational Chemistry

All calculations were performed in ORCA 6.0. TD-DFT calculations were performed using the PBE0 functional[29] with a def2-SVP basis set[30] on geometries obtained from single crystal X-ray diffraction structures. For the macrocycle crystals, we used one unit cell containing a single macrocycle (**Supp. Fig. 8-1**) and chloroform molecules found in the structure were removed for simplicity. For the host–guest crystals, we used one macrocycle and two coronene molecules taken from the co-crystal structure (**Supp. Fig. 8-2**). We focussed on the lowest energy transition since CD is observed at the absorption edge of the crystals by MMP. The frontier molecular orbital distribution of the major contribution to the lowest energy transition (HOMO-LUMO transition) are shown in **Supp. Figs. 8-1** and **8-2**. The |g| values were calculated from the resulting electric ($\boldsymbol{\mu}$) and magnetic ($\boldsymbol{m}$) dipole transition moment vectors following the relationship $|g| = 4(|\boldsymbol{m}|/|\boldsymbol{\mu}|)\cos(\theta)$.[31,32]

Supplementary Table 8-1: Predicted first vertical transition for the macrocycle crystal unit cell and the macrocycle-coronene complex asymmetric unit. 'au' = atomic units.

	**Macrocycle**	**Macrocycle-coronene**
**λ** (nm)	570	625
$\boldsymbol{\mu_X}$ (au)	-0.198	0.206
$\boldsymbol{\mu_Y}$ (au)	0.189	-0.143
$\boldsymbol{\mu_Z}$ (au)	-0.065	-0.180
$\boldsymbol{\mu^2}$ (au^2)	0.079	0.095
$\boldsymbol{|\mu|}$ (au)	0.281	0.310
$\boldsymbol{f_{osc}}$	0.004	0.005
$\boldsymbol{m_X}$ (au)	-0.026	-0.060
$\boldsymbol{m_Y}$ (au)	-0.047	-0.018
$\boldsymbol{m_Z}$ (au)	-0.061	0.065
$\boldsymbol{m^2}$ (au^2)	0.007	0.008
$\boldsymbol{|m|}$ (au)	0.081	0.090
**θ** (°)	89	140
**Rotatory strength** (1e40*cgs)	-0.098	10.082
**|g|**	0.011	0.899

Orbital Diagrams

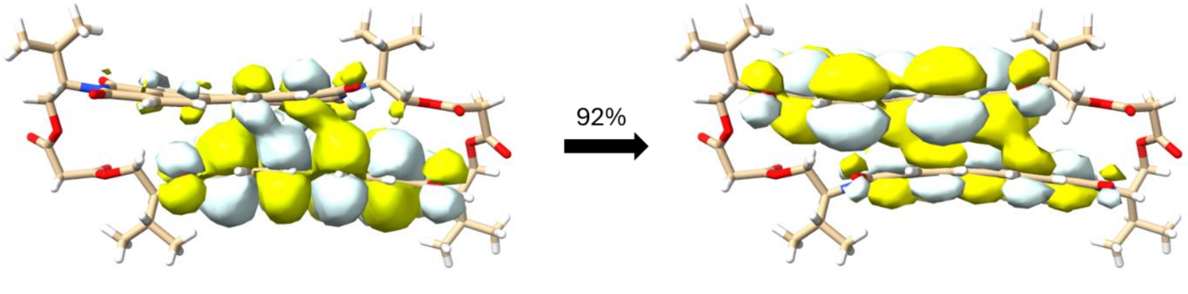

Supplementary Figure 8-1: Orbital diagram of major contribution to the lowest energy transition (92%) for the macrocycle taken from the single crystal. Transition corresponds to the HOMO-LUMO transition.

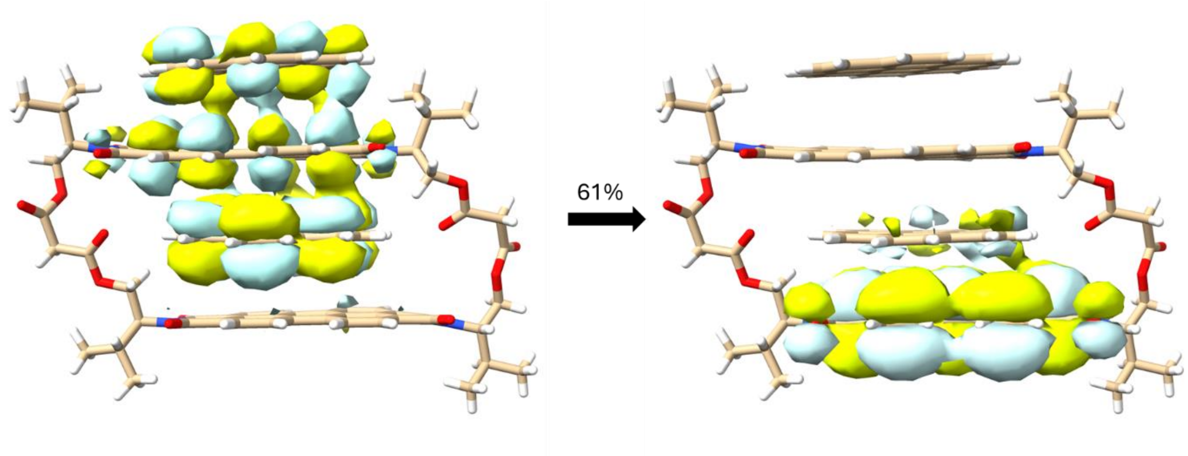

Supplementary Figure 8-2: Orbital diagram of the main contribution towards the first vertical transition (62%) for the asymmetric unit of the macrocycle-coronene complex co-crystal.

9) References

[1] Y.-C. Tao, Z.-Q. Li, J. Yao, Y.-W. Zhong, “Coassembled Ionic Organic Microcrystals with Bright Circularly Polarized Luminescence” *Crystal Growth & Design* **2024**, *24*, 9126–9132.

[2] X. Pan, A. Zheng, X. Yu, Q. Di, L. Li, P. Duan, K. Ye, P. Naumov, H. Zhang, “A Low-Temperature-Resistant Flexible Organic Crystal with Circularly Polarized Luminescence” *Angewandte Chemie International Edition* **2022**, *61*, e202203938.

[3] J. Li, C. Hou, C. Huang, S. Xu, X. Peng, Q. Qi, W.-Y. Lai, W. Huang, “Boosting Circularly Polarized Luminescence of Organic Conjugated Systems via Twisted Intramolecular Charge Transfer” *Research* **2020**, *2020*, DOI 10.34133/2020/3839160.

[4] S. E. Penty, G. R. F. Orton, D. J. Black, R. Pal, M. A. Zwijnenburg, T. A. Barendt, “A Chirally Locked Bis-perylene Diimide Macrocycle: Consequences for Chiral Self-Assembly and Circularly Polarized Luminescence” *J. Am. Chem. Soc.* **2024**, *146*, 5470–5479.

[5] M. Hu, F.-Y. Ye, C. Du, W. Wang, T.-T. Zhou, M.-L. Gao, M. Liu, Y.-S. Zheng, “Tunable Circularly Polarized Luminescence from Single Crystal and Powder of the Simplest Tetraphenylethylene Helicate” *ACS Nano* **2021**, *15*, 16673–16682.

[6] Z.-Q. Li, L. Meng, Z. Chen, Y.-W. Zhong, “Endowing single-crystal polymers with circularly polarized luminescence” *Nat Commun* **2025**, *16*, 234.

[7] C. Du, X. Zhu, C. Yang, M. Liu, “Stacked Reticular Frame Boosted Circularly Polarized Luminescence of Chiral Covalent Organic Frameworks” *Angewandte Chemie International Edition* **2022**, *61*, e202113979.

[8] Z. Zhou, G. Cai, Z. Zhang, G. Li, D. Lou, S. Qu, Y. Li, M. Huang, W. Liu, Z. Zheng, J. Sun, “Conformational Chirality of Single-Crystal Covalent Organic Frameworks” *J. Am. Chem. Soc.* **2024**, *146*, 34064–34069.

[9] J. Cui, H. Wang, H. Liu, H. Yu, W. Wang, Y. Wang, Y. Zhao, “Single-crystal chiral two-dimensional supramolecular organic frameworks for tunable circularly polarized luminescence” *Chem. Sci.* **2025**, *16*, 7513–7522.

[10] Z. Wang, Y. Gai, A. Hao, P. Xing, “Superhelical Self-Assembly of Microcrystals from Cyclodipeptides” *Angewandte Chemie International Edition* **2025**, *64*, e202501832.

[11] X. Pan, L. Lan, L. Li, P. Naumov, H. Zhang, “Flexible Organic Chiral Crystals with Thermal and Excitation Modulation of the Emission for Information Transmission, Writing, and Storage” *Angewandte Chemie International Edition* **2024**, *63*, e202320173.

[12] D. Hartmann, S. E. Penty, M. A. Zwijnenburg, R. Pal, T. A. Barendt, “A Bis-Perylene Diimide Macrocycle Chiroptical Switch” *Angewandte Chemie International Edition* **2025**, e202501122.

[13] T. J. Ugras, Y. Yao, R. D. Robinson, “Can we still measure circular dichroism with circular dichroism spectrometers: The dangers of anisotropic artifacts” *Chirality* **2023**, *35*, 846–855.

[14] T. Harada, H. Moriyama in *Encyclopedia of Polymer Science and Technology*, John Wiley & Sons, Ltd, **2013**.

[15] R. Hussain, T. Jávorfi, G. Siligardi, “CD Imaging at High Spatial Resolution at Diamond B23 Beamline: Evolution and Applications” *Front. Chem.* **2021**, *9*, DOI 10.3389/fchem.2021.616928.

[16] A. T. Martin, S. M. Nichols, V. L. Murphy, B. Kahr, “Chiroptical anisotropy of crystals and molecules” *Chem. Commun.* **2021**, *57*, 8107–8120.

[17] R. Hussain, N. Krumpa, J. Strachan, D. Clarke, U. Wagner, B. Macdonald, D. Reading, T. Cobb, I. Gillingham, A. Price, M. Smith, G. Siligardi, “Design of b23 circular dichroism beamline at diamond light source” *Adv. Syn. Rad.* **2008**, *01*, 265–270.

[18] C. Gedeon, N. Del Rio, F. Furlan, A. Taddeucci, N. Vanthuyne, V. G. Gregoriou, M. J. Fuchter, G. Siligardi, N. Gasparini, J. Crassous, C. L. Chochos, “Rational Design of New Conjugated Polymers with Main Chain Chirality for Efficient Optoelectronic Devices: Carbo[6]Helicene and Indacenodithiophene Copolymers as Model Compounds” *Advanced Materials* **2024**, *36*, 2314337.

[19] R. Chowdhury, M. D. Preuss, H.-H. Cho, J. J. P. Thompson, S. Sen, T. K. Baikie, P. Ghosh, Y. Boeije, X. W. Chua, K.-W. Chang, E. Guo, J. van der Tol, B. W. L. van den Bersselaar, A. Taddeucci, N. Daub, D. M. Dekker, S. T. Keene, G. Vantomme, B. Ehrler, S. C. J. Meskers, A. Rao, B. Monserrat, E. W. Meijer, R. H. Friend, "Circularly polarized electroluminescence from chiral supramolecular semiconductor thin films" *Science* **2025**, *387*, 1175–1181.
[20] G. Pancotti, C. E. Killalea, T. W. Rees, L. Liirò-Peluso, S. Riera-Galindo, P. H. Beton, M. Campoy-Quiles, G. Siligardi, D. B. Amabilino, "Film thickness dependence of nanoscale arrangement of a chiral electron donor in its blends with an achiral electron acceptor" *Nanoscale* **2025**, *17*, 3133–3144.
[21] M. Wasiluk, C. Goldmann, M. Bagiński, M. Pawlak, PawełW. Majewski, J. Abramowicz, P. Roszkowski, L. Rebholz, C. Rockstuhl, C. Hamon, W. Lewandowski, "From Low Symmetry to High Dissymmetry: Chiral Plasmonic Films of Binary and Nanobipyramid Assemblies" *Advanced Functional Materials* **n.d.**, *n/a*, 2500933.
[22] T. J. Ugras, R. B. Carson, R. P. Lynch, H. Li, Y. Yao, L. Cupellini, K. A. Page, D. Wang, A. Arbe, S. Bals, L. Smieska, A. R. Woll, O. Arteaga, T. Jávorfi, G. Siligardi, G. Pescitelli, S. J. Weinstein, R. D. Robinson, "Transforming achiral semiconductors into chiral domains with exceptional circular dichroism" *Science* **2025**, *387*, eado7201.
[23] G. Albano, M. Bertuolo, F. Zinna, A. Taddeucci, T. Jávorfi, R. Hussain, G. M. Farinola, G. Pescitelli, A. Punzi, G. Siligardi, L. D. Bari, "Unravelling the origin of strong non-reciprocal chiroptical features in thin films of a chiral diketopyrrolo[3,4-c]pyrrole dye" *Nanoscale* **2025**, *17*, 5128–5140.
[24] John Freudenthal **2018**.
[25] J. J. Gil, E. and Bernabeu, "Depolarization and Polarization Indices of an Optical System" *Optica Acta: International Journal of Optics* **1986**, *33*, 185–189.
[26] R. M. A. Azzam, "Propagation of partially polarized light through anisotropic media with or without depolarization: A differential 4 × 4 matrix calculus" *J. Opt. Soc. Am., JOSA* **1978**, *68*, 1756–1767.
[27] O. Arteaga, A. Canillas, "Analytic inversion of the Mueller-Jones polarization matrices for homogeneous media" *Opt. Lett., OL* **2010**, *35*, 559–561.
[28] R. Carr, R. Puckrin, B. K. McMahon, R. Pal, D. Parker, L.-O. Pålsson, "Induced circularly polarized luminescence arising from anion or protein binding to racemic emissive lanthanide complexes" *Methods Appl. Fluoresc.* **2014**, *2*, 024007.
[29] C. Adamo, V. Barone, "Toward reliable density functional methods without adjustable parameters: The PBE0 model" *J. Chem. Phys.* **1999**, *110*, 6158–6170.
[30] D. Rappoport, F. Furche, "Property-optimized Gaussian basis sets for molecular response calculations" *J. Chem. Phys.* **2010**, *133*, 134105.
[31] Y. Nagata, T. Mori, "Irreverent Nature of Dissymmetry Factor and Quantum Yield in Circularly Polarized Luminescence of Small Organic Molecules" *Front. Chem.* **2020**, *8*, DOI 10.3389/fchem.2020.00448.
[32] R. G. Uceda, C. M. Cruz, S. Míguez-Lago, L. Á. de Cienfuegos, G. Longhi, D. A. Pelta, P. Novoa, A. J. Mota, J. M. Cuerva, D. Miguel, "Can Magnetic Dipole Transition Moment Be Engineered?" *Angewandte Chemie International Edition* **2024**, *63*, e202316696.
